# Efficacy and harms of tocilizumab for the treatment of COVID-19 patients: A systematic review and meta-analysis

**DOI:** 10.1371/journal.pone.0269368

**Published:** 2022-06-03

**Authors:** Alejandro Piscoya, Angela Parra del Riego, Renato Cerna-Viacava, Jonathon Rocco, Yuani M. Roman, Angel A. Escobedo, Vinay Pasupuleti, C. Michael White, Adrian V. Hernandez

**Affiliations:** 1 Unidad de Revisiones Sistemáticas y Meta-análisis (URSIGET), Universidad San Ignacio de Loyola (USIL), Lima, Peru; 2 Hospital Guillermo Kaelin de La Fuente, Lima, Peru; 3 Escuela de Medicina, Universidad Peruana de Ciencias Aplicadas (UPC), Lima, Peru; 4 Department of Medicine, Henry Ford Hospital, Detroit, Michigan, United States of America; 5 Health Outcomes, Policy, and Evidence Synthesis (HOPES) Group, University of Connecticut School of Pharmacy, Storrs, Connecticut, United States of America; 6 Epidemiology Unit, National Institute of Gastroenterology, La Habana, Cuba; 7 Cello Health, Yardley, Pennsylvania, United States of America; James Cook University, AUSTRALIA

## Abstract

**Introduction:**

We systematically assessed benefits and harms of tocilizumab (TCZ), which is an antibody blocking IL-6 receptors, in hospitalized COVID-19 patients.

**Methods:**

Five electronic databases and two preprint webpages were searched until March 4, 2021. Randomized controlled trials (RCTs) and inverse probability treatment weighting (IPTW) cohorts assessing TCZ effects in hospitalized, COVID-19 adult patients were included. Primary outcomes were all-cause mortality, clinical worsening, clinical improvement, need for mechanical ventilation, and adverse events (AE). Inverse variance random-effects meta-analyses were performed with quality of evidence (QoE) evaluated using GRADE methodology.

**Results:**

Nine RCTs (n = 7,021) and nine IPTW cohorts (n = 7,796) were included. TCZ significantly reduced all-cause mortality in RCTs (RR 0.89, 95%CI 0.81–0.98, p = 0.03; moderate QoE) and non-significantly in cohorts (RR 0.67, 95%CI 0.44–1.02, p = 0.08; very low QoE) vs. control (standard of care [SOC] or placebo). TCZ significantly reduced the need for mechanical ventilation (RR 0.80, 95%CI 0.71–0.90, p = 0.001; moderate QoE) and length of stay (MD -1.92 days, 95%CI -3.46 to -0.38, p = 0.01; low QoE) vs. control in RCTs. There was no significant difference in clinical improvement or worsening between treatments. AEs, severe AEs, bleeding and thrombotic events were similar between arms in RCTs, but there was higher neutropenia risk with TCZ (very low QoE). Subgroup analyses by disease severity or risk of bias (RoB) were consistent with main analyses. Quality of evidence was moderate to very low in both RCTs and cohorts.

**Conclusions:**

In comparison to SOC or placebo, TCZ reduced all-cause mortality in all studies and reduced mechanical ventilation and length of stay in RCTs in hospitalized COVID-19 patients. Other clinical outcomes were not significantly impacted. TCZ did not have effect on AEs, except a significant increased neutropenia risk in RCTs. TCZ has a potential role in the treatment of hospitalized COVID-19 patients.

## Introduction

Over 500 million people have contracted COVID-19, contributing to the ~6.2 million total deaths worldwide [1]. Those with comorbidities such as obesity, asthma, diabetes mellitus, hypertension, and chronic kidney disease are at higher risk of death [2]. Treatment options for COVID-19 patients are limited, including corticosteroids in hospitalized patients requiring supplemental oxygen or remdesivir in those hospitalized patients requiring supplemental oxygen but not mechanically ventilated [3].

Tocilizumab (TCZ) is an intravenous monoclonal antibody that works by blocking the interleukin-6 receptor, which activates this prominent inflammatory cytokine [4]. TCZ was regarded as a potential treatment in hospitalized, severe COVID-19 patients. One limitation of TCZ use is that it costs between $2,700-$5,400 for a single 400 mg to 800 mg doses, which can be used up to two doses in the setting of COVID-19. [5].

Published guidelines of good quality that use Grading of Recommendations Assessment, Development and Evaluation (GRADE) methodology recommend the use of TCZ in COVID-19. The Infectious Diseases Society of America (IDSA) guidelines, as of August 31, 2021, suggested the use of TCZ in hospitalized patients with progressive severe or critical COVID-19 who have elevated markers of inflammation such as C-reactive protein, serum ferritin, LDH, and IL-6. [6]. The Australian COVID-19 guidelines of April 12, 2022 recommended considering TCZ for the treatment of COVID-19 in adults who require supplemental oxygen, particularly in those with evidence of systemic inflammation [7].

The National Institute for Health and Care Excellence (NICE) of the United Kingdom recommended on April 12, 2022 to offer TCZ to hospitalized adults with COVID-19 who are having or have completed a course of corticosteroids, did not receive another IL-6 inhibitor, and had no evidence of a bacterial or other viral infection. Also, patients need supplemental oxygen and a C-reactive protein > = 75mg/L, or are within 48h of starting high-flow nasal oxygen, non-invasive ventilation or invasive mechanical ventilation [8]. The Pan-American Health Organization of the World Health Organization (PAHO/WHO), as of April 12, 2022, described that TCZ reduced mortality and mechanical ventilation requirements with high quality of evidence, without significantly increasing severe adverse events [9].

We systematically assessed randomized controlled trials (RCTs) and higher quality cohort studies to determine the effects of TCZ on clinical outcomes and adverse events in hospitalized COVID-19 patients.

## Methods

### Data sources and searches

Two investigators (V.P., and A.V.H.) developed the search strategy, which was revised and approved by the other investigators. We searched the following databases until March 4, 2021: PubMed-MEDLINE, EMBASE-OVID, Scopus, Web of Science, the Cochrane Library, medRxiv.org (www.medrxiv.org) and Preprints (www.preprints.org). The PubMed search strategy is shown in the S1 File. There was no language limitation.

### Study selection

We included controlled studies (RCTs and cohort studies using inverse probability treatment weighting [IPTW]) in any language reporting benefit or harm outcomes of TCZ as treatment in hospitalized COVID-19 patients. IPTW cohorts allow for balancing treatment groups for patient characteristics, thus making cohorts the closest to a target RCT. IPTW achieves this by using patient weighting and propensity score matching to best estimate the population average treatment effect [10, 11]. We excluded studies in non-hospitalized COVID-19 patients. Four investigators (A.P.dR., R.C-V., A.V.H., V.P.) independently screened each record title and abstract for potential inclusion. Three investigators (A.P.dR., R.C-V., A.V.H.,) then assessed full texts of selected abstracts. Discrepancies were resolved through discussion or by another investigator (A.P.).

### Outcomes

Primary outcomes were all-cause mortality, clinical worsening and improvement, need for mechanical ventilation, and adverse events. Secondary outcomes were length of hospital stay, and severe adverse events as stated by individual authors. Adverse events included bacteremia/infection, abnormal liver function, bleeding events, neutropenia, and thrombotic events. Clinical worsening and improvement were extracted as defined by authors. All outcomes were measured by authors at a timeframe of 14 to 28 days.

### Data extraction

Three investigators (A.P., A.P.dR., R.C-V.) independently extracted the following variables from studies: study design (RCT vs. IPTW cohort), study setting, country(ies), mean age, proportion of male participants, co-morbidities such as hypertension, diabetes mellitus and heart disease, inflammatory and thrombotic biomarkers, severity of COVID-19 disease according to WHO classification [12], TCZ dose and duration, type of control and description, additional drug interventions, primary and secondary outcomes, and time of follow up. Discrepancies were resolved through discussion or by another investigator (A.V.H.).

### Risk of bias assessment

Three investigators (A.P., A.P.dR., R.C-V.) in pairs, independently assessed risk of bias (RoB) by using the ROBINS-I (Risk Of Bias In Non-Randomized Studies of Interventions) tool for cohorts [13] and the Cochrane RoB 2.0 tool for RCTs [14]; disagreements were resolved by discussion with a third investigator (A.V.H.). RoB per domain and per study were described as low, moderate, serious, critical and no information for IPTW cohort studies, and as low, some concerns and high for RCTs. RoB 2.0 evaluates five bias domains: randomization process, deviations from intended interventions, missing outcome data, measurement of the outcome and selection of the reported result. ROBINS-I evaluates seven bias domains: due to confounding, selection of participants into the study, classification of interventions, deviations from intended interventions, missing outcome data, measurement of the outcome and selection of the reported result.

### Statistical analyses

We reported our systematic review according to 2020 PRISMA guidelines [15]. We primarily stratified our analyses by study design (RCT vs. IPTW cohort). Inverse variance random effect meta-analyses were performed to evaluate effect of TCZ vs. control on outcomes. Effects of meta-analyses were reported as relative risks (RR) for dichotomous outcomes and as mean difference (MD) for the continuous outcome such as length of stay. The 95% confidence intervals (CIs) of effects were adjusted with the Hartung-Knapp method [16], and the between study variance tau^2^ was calculated with the Paule-Mandel method [17]. Heterogeneity of effects among studies was quantified with the I^2^ statistic (an I^2^>60% means high heterogeneity). We pre-specified subgroup analyses by severity of COVID-19 disease and RoB; the p for interaction test <0.1 indicated effect modification by subgroup. The *meta* package of R 3.5.1 (www.r-project.org) was used for meta-analyses. The quality of evidence was evaluated using the GRADE methodology, which covers five aspects: risk of bias, inconsistency, indirectness, imprecision, and publication bias [18]. Quality of evidence (QoE) was evaluated per outcome, and described in summary of findings (SoF) tables; GRADEpro GDT was used to create SoF tables [19]. According to the Cochrane Handbook version 6.2 (February 2021), tests for funnel plot asymmetry should be used only when there are at least ten studies included in the meta-analysis, because when there are fewer studies the power of the tests is low (https://training.cochrane.org/handbook/current/chapter-13).

## Results

### Selection of studies

**Fig 1:**
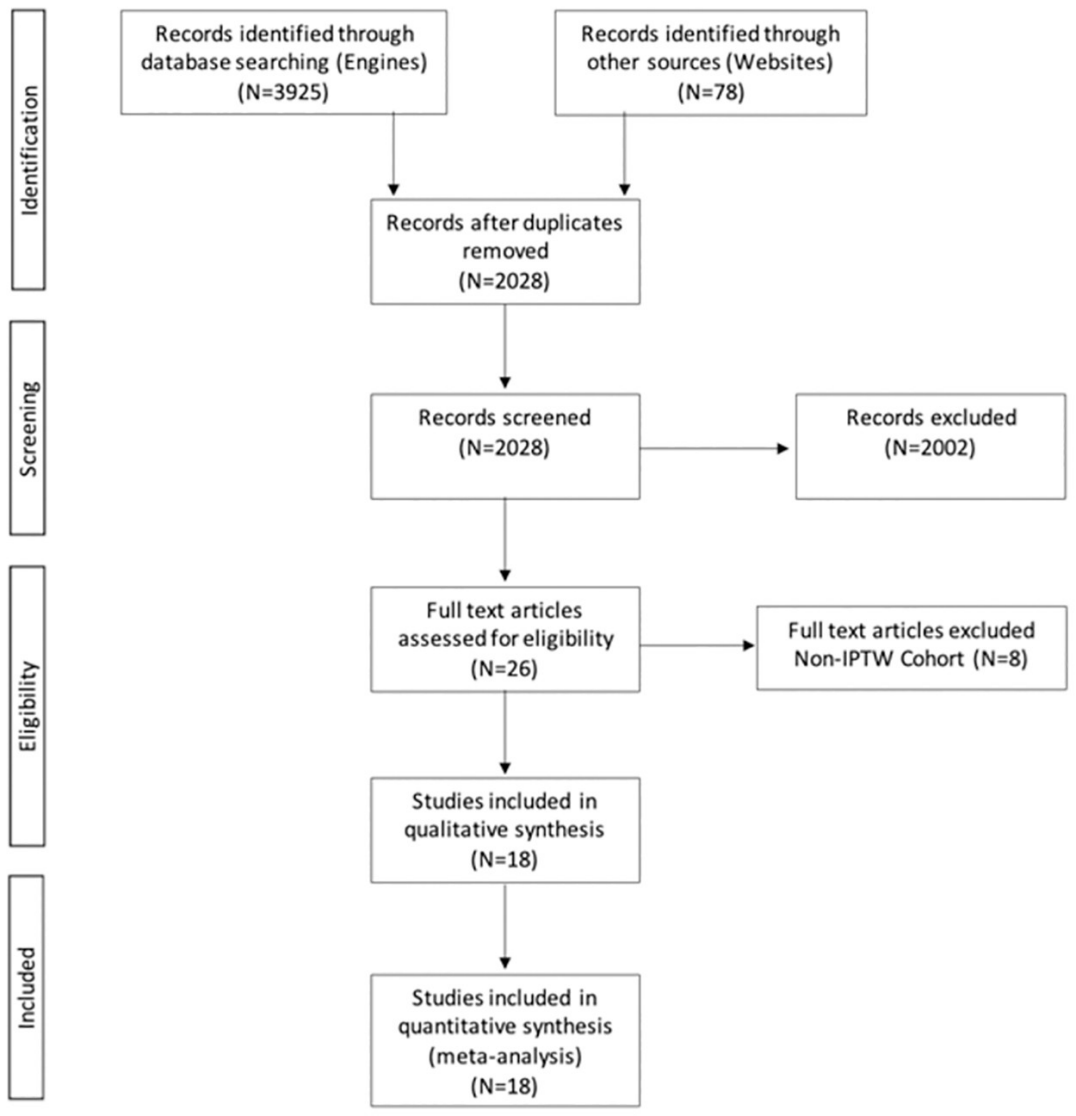
PRISMA 2020 flowchart diagram.

Our comprehensive search yielded 3925 citations with an additional 78 citations identified through other sources. After our assessment, we identified nine RCTs [20–28] (n = 7,021) and nine IPTW cohort studies (29–37) (n = 7,796) which were all homogenous enough to warrant meta-analyses by study design (Fig 1).

### Characteristics of included studies

The general characteristics of the included RCTs and IPTW cohort studies are included in S1 and S2 Tables in S1 File, respectively. Moderate, moderate to severe, and severe hospitalized COVID-19 patients were assessed in two [21, 25], six [22–24, 26–28] and one [20] RCTs, respectively. Also, moderate, moderate to severe, severe, and unspecified severity in hospitalized COVID-19 patients were assessed in three [34–36], two [30, 32], three [29, 31, 37], and one [33] IPTW cohorts, respectively. Standard of care (SoC) and SoC plus placebo were the comparators in six [20, 21, 24, 26–28] and three [22, 23, 25] RCTs, respectively. SoC was the comparator for all nine IPTW cohorts. The nine RCTs used TCZ doses of 8 mg/kg, and five of them used a second dose [20, 21, 24, 27, 28]; follow-up times ranged between 14 and 28 days. The IPTW cohort studies had reported a wider variety of dosing ranging from 4–8 mg/kg or some using total daily dose of 400-800mg. There were two studies with no dose reported [31, 34] and four studies reporting a second dose [29, 32, 35, 36]. Follow-up on the cohorts ranged from 21 to 47 days with one study not specifying a follow-up period [33].

### Risk of bias of included studies

The RCT by Veiga et al. had high risk of bias due to selection of the reported results [26], six RCTs had some concerns of risk of bias due to randomization process, deviations from intended intervention, and selection of the reported results. Hermine et al. [21] and Horby et al. [27] had low risk of bias (S1 Fig in S1 File). Among the IPTW cohorts, Biran et al. [29], Chilimuri et al. [30], Martínez-Sanz et al. [33], Rodríguez-Bano et al. [34], Roumier et al. [36] and Somers et al. [37] were at serious risk of bias. Gupta et al. [31], Hill et al. [32], and Rossi et al. [35] were at moderate risk of bias (S2 Fig in S1 File). Details of assessments of each individual RCT and IPTW cohorts are available in the S2 and S3 Files, respectively.

### Effects of tocilizumab on clinical outcomes

**Fig 2:**
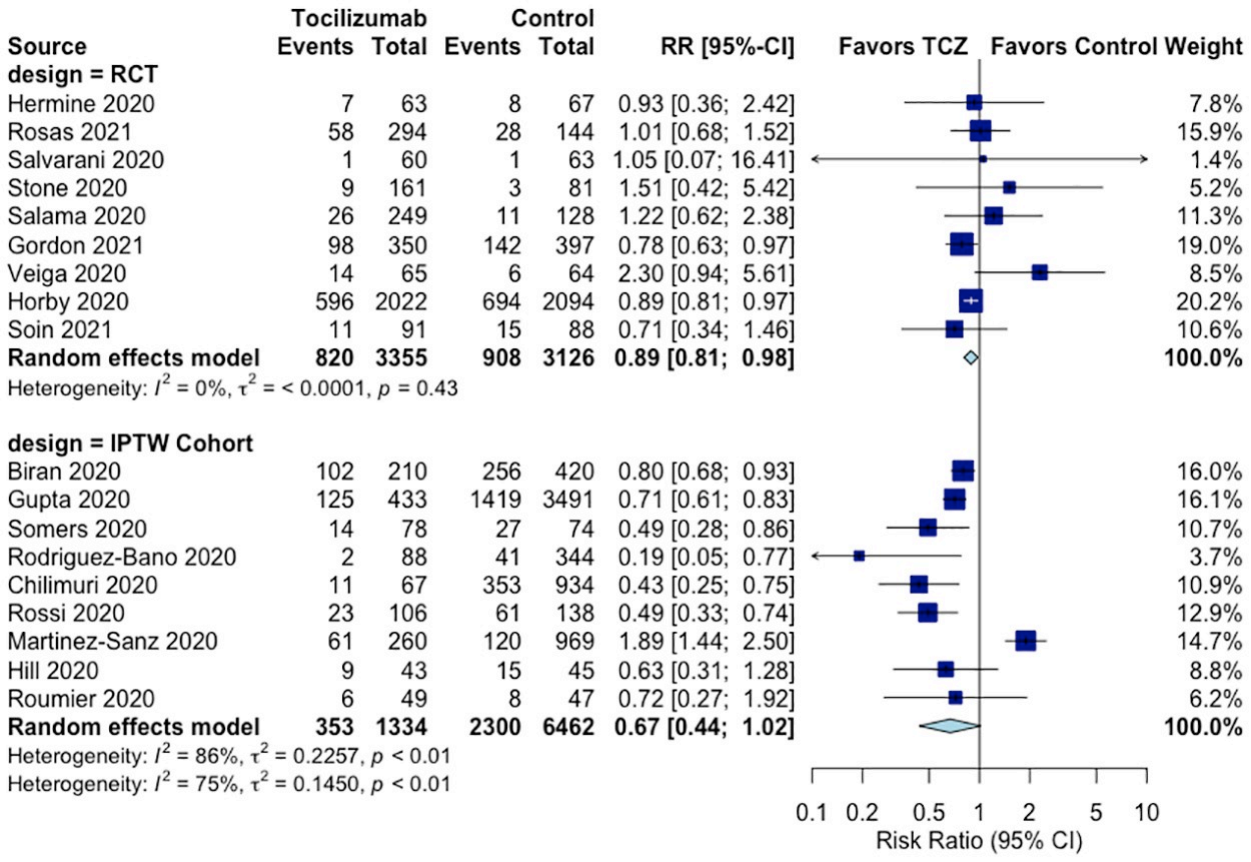
Effect of tocilizumab on all-cause mortality in RCTs and IPTW cohort studies of hospitalized COVID-19 patients.

The dataset and R syntax used for meta-analyses are available in the S4 and S5 Files. In comparison to the control group, TCZ significantly reduced all-cause mortality by 11% (RR 0.89, 95%CI 0.81–0.98, p = 0.03, I^2^ = 0%, moderate QoE) in the nine RCTs. In comparison to the control group, TCZ non-significantly reduced all-cause mortality by 33% (RR 0.67, 95%CI 0.44–1.02, p = 0.08, I^2^ = 86%, very low QoE) in nine IPTW cohorts (Fig 2, Tables 1 and 2).

**Table 1:** Summary of Findings table of tocilizumab compared to standard of care for COVID-19 in RCTs.

Outcomes	Anticipated absolute effects* (95% CI)		Relative effect (95% CI)	№ of participants (studies)	Certainty of the evidence (GRADE)
	Risk with standard of care	Risk with tocilizumab			
All-cause mortality follow up: range 14 days to 28 days	29 per 100	**26 per 100** (24 to 28)	**RR 0.89** (0.81 to 0.98)	6481 (9 RCTs)	⨁⨁⨁◯ MODERATE ^a^
Clinical improvement assessed with: Discharge at follow up or Decrease in 2points of the 7-point clinical improvement scale follow up: range 14 days to 28 days	75 per 100	**77 per 100** (68 to 87)	**RR 1.02** (0.91 to 1.16)	365 (2 RCTs)	⨁⨁⨁◯ MODERATE ^b^
Clinical worsening assessed with: Mechanical ventilation (MV), death or clinical aggravation (PaO2/FiO2<150); Non-invasive ventilation, high-flow O2, MV or death; Increase in > = 1 point w/O2 at baseline or > = 2 points wo/O2 at baseline in a 7-point scale (the higher the worse); OR death, ICU or MV; COVID-19 progression (moderate to severe and severe to death) follow up: range 14 days to 28 days	27 per 100	**21 per 100** (16 to 28)	**RR 0.79** (0.58 to 1.06)	947 (5 RCTs)	⨁⨁⨁◯ MODERATE ^c^
Mechanical ventilation follow up: range 14 days to 28 days	18 per 100	**15 per 100** (13 to 17)	**RR 0.80** (0.71 to 0.90)	5269 (7 RCTs)	⨁⨁⨁◯ MODERATE ^d^
Length of Stay follow up: range 14 days to 28 days	The mean length of Stay was **10** days	MD **1.92 days lower** (3.46 lower to 0.38 lower)	-	517 (2 RCTs)	⨁⨁◯◯ LOW ^e^^,^^f^
Adverse events follow up: range 14 days to 28 days	18 per 100	**22 per 100** (15 to 32)	**RR 1.20** (0.83 to 1.74)	5504 (7 RCTs)	⨁◯◯◯ VERY LOW ^g^^,^^h^
Severe adverse events follow up: range 14 days to 28 days	5 per 100	**4 per 100** (3 to 5)	**RR 0.91** (0.75 to 1.10)	6501 (9 RCTs)	⨁⨁⨁◯ MODERATE ^a^
Bacteremia/infection follow up: range 14 days to 28 days	4 per 100	**3 per 100** (2 to 4)	**RR 0.78** (0.58 to 1.05)	6501 (9 RCTs)	⨁⨁◯◯ LOW ^a^^,^^i^
Neutropenia follow up: range 14 days to 28 days	0 per 100	**3 per 100** n(1 to 11)	**RR 7.87** (2.14 to 28.93)	624 (4 RCTs)	⨁◯◯◯ VERY LOW ^j^^,^^k^
Bleeding events follow up: range 14 days to 28 days	3 per 100	**4 per 100** (3 to 7)	**RR 1.29** (0.80 to 2.09)	1564 (4 RCTs)	⨁⨁◯◯ LOW ^l^^,^^m^
Thrombotic events follow up: range 14 days to 28 days	3 per 100	**1 per 100** (1 to 3)	**RR 0.44** (0.19 to 1.04)	1436 (5 RCTs)	⨁⨁◯◯ LOW ^n^^,^^o^
Abnormal liver function assessed with: Elevation of AST or ALT above upper normal limits follow up: range 14 days to 28 days	5 per 100	**7 per 100** (3 to 15)	**RR 1.24** (0.57 to 2.69)	932 (4 RCTs)	⨁◯◯◯ VERY LOW ^p^^,^^q^^,^^r^

*The risk in the intervention group (and its 95% confidence interval) is based on the assumed risk in the comparison group and the **relative effect** of the intervention (and its 95% CI).

**CI =** Confidence interval; **MD =** Mean difference; **RR =** Risk ratio.

GRADE Working Group grades of evidence

**High certainty**: We are very confident that the true effect lies close to that of the estimate of the effect

**Moderate certainty**: We are moderately confident in the effect estimate: The true effect is likely to be close to the estimate of the effect, but there is a possibility that it is substantially different

**Low certainty**: Our confidence in the effect estimate is limited: The true effect may be substantially different from the estimate of the effect

**Very low certainty**: We have very little confidence in the effect estimate: The true effect is likely to be substantially different from the estimate of effect

Explanations

^a^. RoB: Veiga et al. has high risk of bias due to selection of the reported results, Salvarani et al. and Soin et al. have some concerns on randomization process and deviation from intended interventions, Stone et al. has some concerns on randomization process and selection of reported result, Rosas et al. has some concerns on the randomization process, Salama et al. has some concerns on deviation from intended interventions and selection of the reported result, Gordon et al. has some concerns on selection of the reported results. Hermine et al. and Horby et al. have low risk of bias.

^b^. RoB: Salvarani et al. has some concerns on randomization process and deviation from intended interventions, Stone et al. has some concerns on randomization process and selection of reported result.

^c^. RoB: Salvarani et al. and Soin et al. have some concerns on randomization process and deviation from intended interventions, Stone et al. has some concerns on randomization process and selection of reported result, Rosas et al. has some concerns on the randomization process. Hermine et al. has low risk of bias.

^d^. RoB: Veiga et al. has high risk of bias due to selection of the reported results, Soin et al. has some concerns of risk of bias on randomization process and deviation from intended interventions, Stone et al. has some concerns on randomization process and selection of reported result, Rosas et al. has some concerns on the randomization process, Salama et al. has some concerns on deviation from intended interventions and selection of the reported result, Gordon et al. has some concerns on selection of the reported results. Horby et al. has low risk of bias.

^e^. RoB: Veiga et al. has high risk of bias due to selection of the reported results, Salama et al. has some concerns on deviation from intended interventions and selection of the reported result.

^f^. Imprecision: 95% CI goes from -11.92 to 8.08

^g^. RoB: Veiga et al. has high risk of bias due to selection of the reported results, Salvarani et al. and Soin et al. have some concerns on randomization process and deviation from intended interventions, Rosas et al. has some concerns on the randomization process, Salama et al. has some concerns on deviation from intended interventions and selection of the reported result. Hermine et al. and Horby et al. have low risk of bias.

^h^. Inconsistency: I2 = 82%

^i^. Imprecision: 95%CI goes from 0.58 to 1.05

^j^. RoB: Veiga et al. has high risk of bias due to selection of the reported results, Salvarani et al. has some concerns on randomization process and deviation from intended interventions, Stone et al. has some concerns on randomization process and selection of reported result. Hermine et al. has low risk of bias.

^k^. Imprecision: 95% CI goes from -3.46 to -0.38 days

^l^. RoB: Veiga et al. has high risk of bias due to selection of the reported results, Stone et al. has some concerns on randomization process and selection of reported result, Rosas et al. has some concerns on the randomization process, Gordon et al. has some concerns on selection of the reported results.

^m^. Imprecision: 95%CI goes from 0.80 to 2.09

^n^. RoB: Veiga et al. has high risk of bias due to selection of the reported results, Soin et al. has some concerns on randomization process and deviation from intended interventions, Stone et al. has some concerns on randomization process and selection of reported result, Gordon et al. has some concerns on selection of the reported results. Hermine et al. has low risk of bias.

^o^. Imprecision: 95%CI goes from 0.19 to 1.04

^p^. RoB: Veiga et al. has high risk of bias due to selection of the reported results, Salvarani et al. has some concerns on randomization process and deviation from intended interventions, Stone et al. has some concerns on randomization process and selection of reported result, Rosas et al. has some concerns on the randomization process.

^q^. Inconsistency: I2 = 45%

^r^. Imprecision: 95%CI goes from 0.57 to 2.69

**Table 2:** Summary of findings table of tocilizumab compared to standard of care for COVID-19 in IPTW cohorts.

Outcomes	Anticipated absolute effects* (95% CI)		Relative effect (95% CI)	№ of participants (studies)	Certainty of the evidence (GRADE)
	Risk with standard of care	Risk with tocilizumab			
All-cause mortality follow up: range 6 days to 47 days	36 per 100	**24 per 100** (16 to 36)	**RR 0.67** (0.44 to 1.02)	7796 (9 observational studies)	⨁◯◯◯ VERY LOW ^a^^,^^b^
Clinical improvement assessed with: Discharge during follow up or undefined improvement follow up: range 6 days to 47 days	66 per 100	**70 per 100** (47 to 100)	**RR 1.07** (0.72 to 1.60)	336 (3 observational studies)	⨁⨁◯◯ LOW ^c^
Clinical worsening assessed with: Increase in scale of 1 point in 7-point scale (higher score the worse) follow up: range 6 days to 47 days	33 per 100	**28 per 100** (15 to 53)	**RR 0.84** (0.44 to 1.58)	88 (1 observational study)	⨁⨁◯◯ LOW ^d^^,^^e^
Mechanical ventilation follow up: range 6 days to 47 days	14 per 100	**13 per 100** (4 to 41)	**RR 0.98** (0.32 to 3.01)	616 (3 observational studies)	⨁◯◯◯ VERY LOW ^f^^,^^g^^,^^h^
Length of Stay follow up: range 6 days to 47 days	The mean length of Stay was **8.8** days	MD **3.23 days higher** (2.41 lower to 8.86 higher)	-	1381 (2 observational studies)	⨁◯◯◯ VERY LOW ^i^^,^^j^^,^^k^
Bacteremia/infection follow up: range 6 days to 47 days	27 per 100	**32 per 100** (21 to 51)	**RR 1.19** (0.76 to 1.87)	5317 (6 observational studies)	⨁◯◯◯ VERY LOW ^l^^,^^m^
Neutropenia follow up: range 6 days to 47 days	0 per 100	**0 per 100** (0 to 0)	**RR 7.66** (0.25 to 235.22)	184 (2 observational studies)	⨁⨁◯◯ VERY LOW ^n^^,^^o^^,^^p^
Bleeding events follow up: range 6 days to 47 days	1 per 100	**1 per 100** (0 to 12)	**RR 1.94** (0.18 to 21.12)	429 (1 observational study)	⨁◯◯◯ VERY LOW ^q^^,^^r^
Thrombotic events follow up: range 6 days to 47 days	10 per 100	**11 per 100** (8 to 15)	**RR 1.13** (0.86 to 1.49)	4108 (3 observational studies)	⨁⨁⨁◯ MODERATE ^s^
Abnormal liver function assessed with: Elevation of AST or ALT above upper normal limits follow up: range 6 days to 47 days	19 per 100	**26 per 100** (22 to 31)	**RR 1.40** (1.19 to 1.64)	4108 (3 observational studies)	⨁⨁⨁◯ MODERATE ^s^

*The risk in the intervention group (and its 95% confidence interval) is based on the assumed risk in the comparison group and the **relative effect** of the intervention (and its 95% CI).

**CI =** Confidence interval; **MD =** Mean difference; **RR =** Risk ratio.

GRADE Working Group grades of evidence

**High certainty**: We are very confident that the true effect lies close to that of the estimate of the effect

**Moderate certainty**: We are moderately confident in the effect estimate: The true effect is likely to be close to the estimate of the effect, but there is a possibility that it is substantially different

**Low certainty**: Our confidence in the effect estimate is limited: The true effect may be substantially different from the estimate of the effect

**Very low certainty**: We have very little confidence in the effect estimate: The true effect is likely to be substantially different from the estimate of effect

Explanations

^a^. ROBINS-I: Chilimuri et al., Biran et al., Somers et al. and Rodriguez-Baño et al. have serious risk of bias due to confounding; Martinez-Sanz et al. has serious risk of bias due to missing data and selection of reported results; Roumier et al. has serious risk of bias in selection of participants into the study; Rossi et al. and Gupta et al. have moderate risk of bias due to confounding, selection of participants into the study, classification of interventions, and deviations from intended interventions; Hill et al. has moderate risk of bias due to confounding, classification of interventions, and deviations from intended interventions.

^b^. Inconsistency: I2 = 86%

^c^. ROBINS-I: Somers et al. has serious risk of bias due to confounding; Roumier et al. has serious risk of bias in selection of participants into the study; Hill et al. has moderate risk of bias due to confounding, classification of interventions, and deviations from intended interventions.

^d^. ROBINS-I: Hill et al. has moderate risk of bias due to confounding, classification of interventions, and deviations from intended interventions.

^e^. Imprecision: 95%CI goes from 0.44 to 1.58

^f^. ROBINS-I: Rodriguez-Baño et al. has serious risk of bias due to confounding; Roumier et al. has serious risk of bias in selection of participants into the study; Hill et al. has moderate risk of bias due to confounding, classification of interventions, and deviations from intended interventions.

^g^. Inconsistency: I2 = 64%

^h^. Imprecision: 95%CI goes from 0.32 to 3.01

^i^. ROBINS-I: Somers et al. has serious risk of bias due to confounding; Martinez-Sanz et al. has serious risk of bias due to missing data and selection of reported results.

^j^. Inconsistency: I2 = 86%

^k^. Imprecision: 95%CI goes from -2.41 to 8.86 days.

^l^. ROBINS-I: Biran et al., Somers et al. and Rodriguez-Baño et al. have serious risk of bias due to confounding; Roumier et al. has serious risk of bias in selection of participants into the study; Gupta et al. has moderate risk of bias due to confounding, selection of participants into the study, classification of interventions, and deviations from intended interventions; Hill et al. has moderate risk of bias due to confounding, classification of interventions, and deviations from intended interventions.

^m^. Inconsistency: I2 = 63%

^n^. ROBINS-I: Roumier et al. has serious risk of bias in selection of participants into the study; Hill et al. has moderate risk of bias due to confounding, classification of interventions, and deviations from intended interventions.

^o^. Inconsistency: I2 = 52%

^p^. Imprecision: 95% CI goes from 0.25 to 235.22

^q^. ROBINS-I: Rodriguez-Bano et al. has serious risk of bias due to confounding.

^r^. Imprecision: 95% CI goes from 0.18 to 21.12

^s^. ROBINS-I: Roumier et al. has serious risk of bias in selection of participants into the study; Gupta et al. has moderate risk of bias due to confounding, selection of participants into the study, classification of interventions, and deviations from intended interventions; Hill et al. has moderate risk of bias due to confounding, classification of interventions, and deviations from intended interventions.

**Fig 3:**
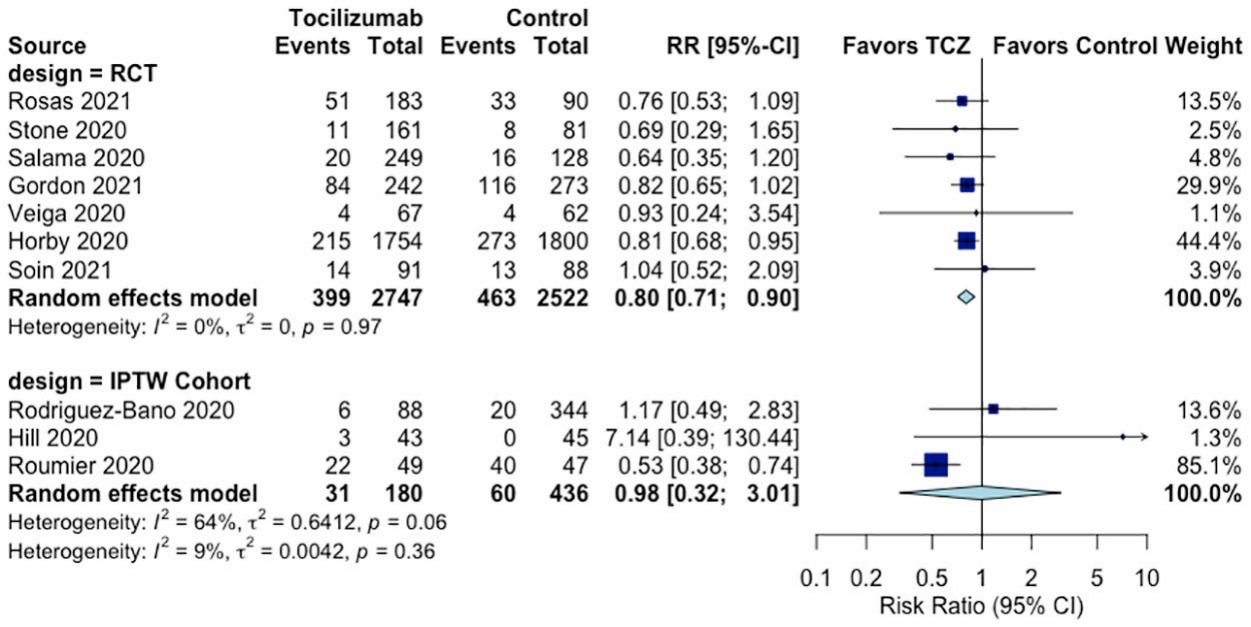
Effect of tocilizumab on mechanical ventilation in RCTs and IPTW cohorts of hospitalized COVID-19 patients.

In comparison to the control group, TCZ non-significantly increased the proportion of clinical improvement in two RCTs [24, 25] and in three [31, 35, 36] IPTW cohorts (S3 Fig in S1 File). TCZ did not decrease the proportion of clinical worsening in five RCTs [21, 22, 24, 25, 28] and in one IPTW cohort [31] (S4 Fig in S1 File). TCZ significantly decreased the need for mechanical ventilation by 20% in seven RCTs [20, 22, 23, 25–28] (RR 0.80, 95%CI 0.71–0.90, p = 0.001, I^2^ = 0%, moderate QoE) and by 2% in three IPTW cohorts [31, 33, 35] (RR 0.98, 95%CI 0.32–3.01, p = 0.9, I^2^ = 64%, very low QoE) (Fig 3, Tables 1 and 2). Lastly, TCZ decreased the length of stay in two RCTs [23, 26] by 1.92 days (95%CI -3.46 to -0.38, p = 0.01, I^2^ = 43%, low QoE), and non-significantly increased length of stay by 3.23 days (95%CI -2.41 to 8.86, p = 0.7, I^2^ = 86%, very low QoE) in two IPTW cohorts [32, 36] (S5 Fig in S1 File, Tables 1 and 2).

### Effects of tocilizumab on adverse events

**Fig 4:**
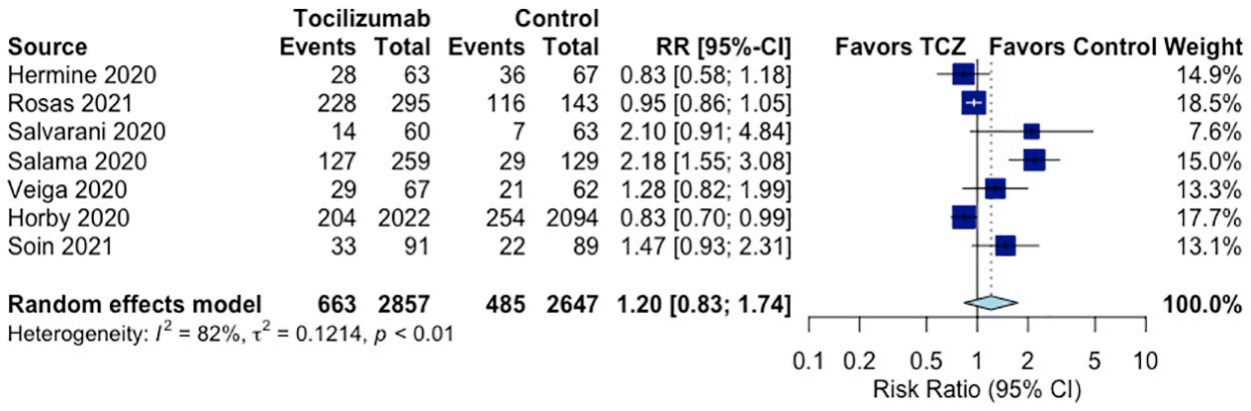
Effect of tocilizumab on adverse events in RCTs of hospitalized COVID-19 patients.

There was no significant difference in the risk of adverse events (RR 1.20, 95%CI 0.83–1.74, p = 0.5, I^2^ = 82%, very low QoE, Fig 4) in seven RCTs [21–24, 26–28] or severe adverse events (RR 0.91, 95%CI 0.75–1.10, p = 0.8, I^2^ = 0%, moderate QoE, S6 Fig in S1 File) in all RCTs [20–28]. The IPTW cohorts did not report on adverse events or severe adverse events.

Compared to controls, TCZ was not associated with bacteremia/infection in all nine RCTs and in six IPTW cohorts [29, 31, 32, 34, 36, 37] (S7 Fig in S1 File); the direction of the effects was different between study designs (p for interaction: 0.01). TCZ significantly increased the risk of neutropenia in four RCTs [21, 24–26] (RR 7.87, 95%CI 2.14–28.93, p = 0.01, I^2^ = 0%, very low QoE) and not significantly in two IPTW cohorts [31, 35] (RR 7.66, 95%CI 0.25–235.22, p = 0.7, I^2^ = 52%, very low QoE) (S8 Fig in S1 File). There were no statistically significant differences in the risks of bleeding events between TCZ and controls in RCTs (RR 1.29, 95%CI 0.80–2.09, p = 0.5, I^2^ = 0%, low QoE) and cohorts (RR 1.94, 95%CI 0.18–21.12, p = 0.9, I^2^ = 0%, very low QoE) (S9 Fig in S1 File). Also, there were no statistically significant differences in the risks of thrombotic events between TCZ and controls in RCTs (RR 0.44, 95%CI 0.19–1.04, p = 0.07, I^2^ = 0%, low QoE) and cohorts (RR 1.13, 95%CI 0.86–1.49, p = 0.4, I^2^ = 0%, moderate QoE) (S10 Fig in S1 File). Finally, TCZ increased the risk of abnormal liver function in three IPTW cohorts [31, 32, 36] (RR 1.40, 95%CI 1.19–1.64, p = 0.02, I^2^ = 0%, moderate QoE) (S11 Fig in S1 File).

### Subgroup analyses

Most of subgroup analyses by severity and risk of bias in both RCTs and IPTW cohorts were consistent with main analyses (S12 Fig in S1 File).

### Quality of evidence across outcomes

The QoE of outcomes in RCTs was moderate for five outcomes, low for four outcomes, and very low for three outcomes (Table 1). For cohorts, the QoE of outcomes was worse: moderate for two outcomes, low for two outcomes, and very low for six outcomes (Table 2).

## Discussion

### Main findings

We found that the use of TCZ in hospitalized, moderate to severe COVID-19 patients had a significant relative reduction of all-cause mortality of 11% in RCTs and non-significant relative reduction of 33% in IPTW cohorts. The quality of evidence for all-cause mortality was moderate for RCTs and very low for IPTW cohorts. TCZ also significantly reduced the relative risk of need for mechanical ventilation by 20% in RCTs, with moderate quality of evidence and significantly reduced the length of stay by 1.92 days in RCTs, with low quality of evidence. Clinical improvement or worsening were not significantly affected by TCZ in all studies. Adverse events, severe adverse events, bleeding and thrombotic events were not significantly different between TCZ and controls; however, we found in RCTs a significantly higher risk of neutropenia with very low quality of evidence. Subgroup analyses by severity of disease and by risk of bias were consistent with main analyses.

### What is known in the literature about our research question?

Published systematic reviews and meta-analyses [38–43] assessed efficacy and safety outcomes of TCZ in hospitalized patients with COVID-19. From those six, none included the RECOVERY trial by Horby et al. [27], nor used GRADE methodology to assess quality of evidence. Four [38–40, 42] reviews included IPTW cohorts and other observational studies. Khan et al. [40], evaluated studies until January 7^th^ 2021, Hariyanto et al. [39] combined RCTs, cohorts and case-control studies, and Berardicurti et al. [38] did not include RCTs.

Current clinical guidelines recommend the use of TCZ in hospitalized, severe COVID-19 patients based on RCT data. The IDSA updated its guidelines [6] to conditionally recommend TCZ in addition to standard of care (i.e., steroids) among hospitalized adults with progressive severe or critical disease and who have elevated markers of systematic inflammation. The Australian guidelines [7] conditionally recommended the use of TCZ for the treatment of COVID-19 in adults who require supplemental oxygen both within and outside the context of a RCT unless contraindicated (e.g., other active severe infections). This recommendation highlighted that significant cost of TCZ may affect equity based on geographical area and access to the drug.

NICE guidelines [8] recommended TCZ offer TCZ to adults in hospital with COVID-19 if all the following apply: 1) they are having or have completed a course of corticosteroids; 2) they have not had another IL-6 inhibitor during this admission; and 3) there is no evidence of a bacterial or other viral infection that might be worsened by TCZ. Also, they need supplemental oxygen and have a C-reactive protein of 75mg/L or more, or are within 48h of starting high-flow nasal oxygen, continuous positive airway pressure, non-invasive ventilation or invasive mechanical ventilation. Finally, the PAHO guidelines [9] found that current evidence of TCZ showed that reduced mortality and need for mechanical ventilation in RCTs with moderate to high quality of evidence. The National Institutes of Health Guidelines [44] do not use GRADE methodology and recommend as of December 16, 2021 that TCZ can be used in hospitalized COVID-19 patients who require supplemental oxygen, high-flow oxygen, non-invasive ventilation, or mechanical ventilation.

### What our study adds to the literature

We evaluated RCTs and IPTW cohorts with robust samples sizes that assessed the impact of TCZ on all-cause mortality and other clinical outcomes, the doses delivered were generally similar, and hospitalized patients had moderate to severe disease. We pre-defined the inclusion of higher quality IPTW cohort studies, which resemble a randomized trial (i.e., target trial approach). The similar direction of effect for reductions in all-cause mortality in RCTs and IPTW cohorts is a strength and there was mostly low statistical heterogeneity in meta-analyses of RCTs.

Our review supports the hypothesis that anti-inflammatory therapies are efficacious options for prolonging survival in COVID-19 patients. The antiviral monoclonal antibodies bamlanivimab and casirivimab plus imdevimab are efficacious therapies at preventing hospitalization or emergency visit in the early stages of COVID-19 but do not provide benefit patients with more advanced disease [45, 46]. Remdesivir may be effective at hastening clinical recovery of hospitalized COVID-19 patients but does not prolong survival [47, 48]. Before TCZ, only corticosteroids have been shown to prolong patient survival and that benefit was predominantly seen in patients with severe COVID-19 [49]. Importantly, TCZ was studied in addition to standard of care COVID-19 therapies which included corticosteroids in every RCT and IPTW cohort except Hill et al. [32]. As such, TCZ can be considered a viable adjunctive option to have better survival rates in severely-ill patients with COVID-19.

Our findings of the effects of TCZ on all-cause mortality and its quality of evidence in RCTs agreed with the meta-analyses of the IDSA [6], Australian [7], and PAHO/WHO [9] guidelines. We used the same set of RCTs, but guideline authors did not describe their methods in detail to judge slightly different effects. Although effects of need for mechanical ventilation with TCZ are similar among the Australian guidelines, PAHO/WHO guidelines and our study, we have differences in the set of RCTs and the quality of evidence. The Australian guidelines [7] evaluated four RCTs (n = 4248), with high QoE; the PAHO/WHO guidelines [9] evaluated 21 RCTs (n = 7655) with high QoE; we evaluated seven RCTs (n = 5269), and concluded moderate QoE. None of those guidelines [6–9] evaluated specific adverse events.

While we were underpowered to fully assess adverse events, there is a suggestion from our meta-analyses that adverse events were non-significantly more frequent with TCZ and this is supported by the known adverse effects seen when TCZ is used in other diseases [50]. We did find a statistically significant 7.9-fold increase in the risk of neutropenia with TCZ in RCTs and 7.7-fold non-significant increase in IPTW cohorts; however, QoE was very low for both designs particularly due to high RoB and large imprecision of effects. Our assessments of other outcomes like clinical improvement or clinical worsening were underpowered across all studies (701 and 947 patients, respectively). There were no statistically significant differences in the risks of bleeding events and thrombotic events between TCZ and controls in both RCTs and IPTW cohorts, with low heterogeneity of effects within designs, and very low to moderate QoE across outcomes and designs.

Whether TCZ should be used in COVID-19 patients with moderate disease or reserved for severely-ill patients cannot be answered by our systematic review. In RCTs, our survival subgroup analyses in severely-ill patients showed strong reduction in one RCT [20] but in eight moderate or moderate to severe RCTs no significant reductions were seen. However, in IPTW cohorts, TCZ was similarly as effective at improving survival in moderate cases as in more severe cases. For other clinical outcomes and specific adverse events, there were a few studies per severity subgroups (severe, moderate to severe, moderate), and effects were similar across subgroups in most of the analyses. These factors make determining the balance of benefits to harms for tocilizumab in patients with moderate disease difficult.

TCZ is an expensive biological agent for treating COVID-19, so the cost-effectiveness of therapy in moderate disease may not be as strong as in severe disease but this requires further study. In addition, the available supply of TCZ may be an issue for widespread use in COVID-19 while still allowing availability in the treatment of rheumatologic patients. Reserving TCZ for severely-ill COVID-19 patients may preserve the supply of the medication for those most likely to benefit. However, if hospitalizations continue to drop due to public health measures and the impact of SARS-CoV-2 vaccines, there may be ample supply of TCZ for both moderately and severely ill patients with COVID-19.

### Limitations

We acknowledge several limitations in our study. First, there was a lack of reporting of clinical outcomes in many of the RCTs and IPTW cohorts. Second, there was a lack of description the degree of mortality effects among patients with moderate disease vs. severe disease; several studies included a mix of moderate and severe patients. Third, the outcomes clinical improvement and clinical worsening were markedly underpowered, and their definitions were heterogeneous across studies. Fourth, no IPTW cohorts reported on the occurrence of adverse events. Finally, the finding of higher risk of neutropenia was based on few events and studies, and we found this outcome had very low quality of evidence for RCTs and IPTW cohorts.

### Conclusions

Our meta-analysis showed that the use of tocilizumab in patients with moderate to severe COVID-19 reduced all-cause mortality in all studies, and the need for mechanical ventilation and length of stay in RCTs. Also, tocilizumab non-significantly increased clinical improvement in addition to corticosteroid therapy. In addition, we showed no differences in the risk of adverse events or severe adverse events such as bacteremia or infection, but tocilizumab increased the risk of neutropenia, abnormal liver function, and bleeding events vs controls in both RCTs and IPTW cohorts. In particular, tocilizumab significantly increased the risk of neutropenia in RCTs, although quality of evidence was very low. The degree of all-cause mortality benefit and further risks of tocilizumab use in the subset of people with moderate versus severe COVID-19 requires further study.

## Supporting information

S1 File Supplementary information—Contains all supporting tables and figures. (DOCX)

S2 File RoB2.0 assessments of each of the included RCTs. (XLSM)

S3 File ROBINS-I assessments of each of the included IPTW cohorts. (DOCX)

S4 File Excel dataset used for meta-analyses. (XLSX)

S5 File R syntax used for meta-analyses. (R)

S6 File PRISMA 2020 checklist. (DOCX)
