# Supplementary material for: Efficacy and harms of tocilizumab for the treatment of COVID-19 patients: A systematic review and meta-analysis
Source: PLoS One. 2022 Jun 3;17(6):e0269368. doi: 10.1371/journal.pone.0269368 (PMC9165853; doi:10.1371/journal.pone.0269368)
Supplement: S1 File — (DOCX) [file pone.0269368.s001.docx]

**S1 File**

**Efficacy and harms of tocilizumab for the treatment of COVID-19 patients:**

**A systematic review and meta-analysis**

Piscoya A et al.

**Pubmed search strategy**

**S1 Table.** Baseline characteristics of included RCTs.

**S2 Table.** Baseline characteristics of included IPTW cohorts.

**S1 Fig**. Cochrane Risk of Bias 2.0 Tool of included randomized controlled trials.

**S2 Fig.** ROBINS-I Risk of Bias Tool Figure of included cohort studies.

**S3 Fig**. Effect of tocilizumab on clinical improvement in RCTs and IPTW cohorts of hospitalized COVID-19 patients.

**S4 Fig.** Effect of tocilizumab on clinical worsening in RCTs and IPTW cohorts of hospitalized COVID-19 patients.

**S5 Fig**. Effect of tocilizumab on length of hospital stay in RCTs and IPTW cohorts of hospitalized COVID-19 patients.

**S6 Fig**. Effect of tocilizumab on severe adverse events in RCTs of hospitalized COVID-19 patients.

**S7 Fig.** Effect of tocilizumab on bacteremia/infection in RCTs and IPTW cohorts of hospitalized COVID-19 patients.

**S8 Fig.** Effect of tocilizumab on neutropenia in RCTs and IPTW cohorts of hospitalized COVID-19 patients.

**S9 Fig.** Effect of tocilizumab on bleeding events in RCTs and IPTW cohorts of hospitalized COVID-19 patients.

**S10 Fig.** Effect of tocilizumab on thrombotic events in RCTs and IPTW cohorts of hospitalized COVID-19 patients.

**S11 Fig.** Effect of tocilizumab on abnormal liver function in RCTs and IPTW cohorts of hospitalized COVID-19 patients.

**S12 Fig.** Subgroup analyses

Fig S12.1. All-cause mortality

S12.1.1. RCTs

S12.1.1.a. Severity

S12.1.1.b. RoB

S12.1.2. IPTW cohorts

S12.1.2.a. Severity

S12.1.2.b. RoB

Fig S12.2. Clinical improvement

S12.2.1. RCTs

S12.2.1.a. Severity

S12.2.1.b. RoB

S12.2.2. IPTW cohorts

S12.2.2.a. Severity

S12.2.2.b. RoB

Fig S12.3. Clinical worsening

S12.3.1. RCTs

S12.3.1.a. Severity

S12.3.1.b. RoB

S12.3.2. IPTW cohorts

S12.3.2.a. Severity

S12.3.2.b. RoB

Fig S12.4. Mechanical ventilation

S12.4.1. RCTs

S12.4.1.a. Severity

S12.4.1.b. RoB

S12.4.2. IPTW cohorts

S12.4.2.a. Severity

S12.4.2.b. RoB

Fig S12.5. Adverse events

S12.5.1. RCTs

S12.5.1.a. Severity

S12.5.1.b. RoB

Fig S12.6. Severe adverse events

S12.6.1 RCTs

S12.6.1.a. Severity

S12.6.1.b. RoB

Fig S12.7. Bacteremia/infection

S12.7.1 RCTs

S12.7.1.a. Severity

S12.7.1.b. RoB

S12.7.2. IPTW cohorts

S12.7.2.a. Severity

S12.7.2.b. RoB

Fig S12.8. Bleeding events

S12.8.1 RCTs

S12.8.1.a. Severity

S12.8.1.b. RoB

S12.8.2 IPTW cohorts

S12.8.2.a. Severity

S12.8.2.b. RoB

Fig S12.9. Neutropenia

S12.9.1. RCTs

S12.9.1.a. Severity

S12.9.1.b. RoB

S12.9.2. IPTW cohorts

S12.9.2.a. Severity

S12.9.2.b. RoB

Fig S12.10. Thrombotic Events

S12.10.1. RCTs

S12.10.1.a. Severity

S12.10.1.b. RoB

S12.10.2. IPTW cohorts

S12.10.2.a. Severity

S12.10.2.b. RoB

Fig S12.11. Abnormal liver function

S12.11.1. RCTs

S12.11.1.a. Severity

S12.11.1.b. RoB

S12.11.2. IPTW cohorts

S12.11.2.a. Severity

S12.11.2.b. RoB

**PubMed search strategy**

(("tocilizumab"[Supplementary Concept] OR "tocilizumab"[All Fields]) OR (("tocilizumab"[Supplementary Concept] OR "tocilizumab"[All Fields]) OR "actemra"[All Fields])) AND (((((((((((("covid 19"[All Fields] OR "covid 2019"[All Fields]) OR "severe acute respiratory syndrome coronavirus 2"[Supplementary Concept]) OR "severe acute respiratory syndrome coronavirus 2"[All Fields]) OR "2019 ncov"[All Fields]) OR "sars cov 2"[All Fields]) OR "2019ncov"[All Fields]) OR (("wuhan"[All Fields] AND ("coronavirus"[MeSH Terms] OR "coronavirus"[All Fields])) AND (2019/12/1:2019/12/31[Date - Publication] OR 2020/1/1:2020/12/31[Date - Publication]))) OR (("coronavirus"[MeSH Terms] OR "coronavirus"[All Fields]) OR "coronaviruses"[All Fields])) OR ((("coronavirus"[MeSH Terms] OR "coronavirus"[All Fields]) OR "coronaviruses"[All Fields]) AND (((("disease"[MeSH Terms] OR "disease"[All Fields]) OR "diseases"[All Fields]) OR "disease s"[All Fields]) OR "diseased"[All Fields]))) OR (("covid 19"[Supplementary Concept] OR "covid 19"[All Fields]) OR "coronavirus disease 19"[All Fields])) OR (("severe acute respiratory syndrome"[MeSH Terms] OR ((("severe"[All Fields] AND "acute"[All Fields]) AND "respiratory"[All Fields]) AND "syndrome"[All Fields])) OR "severe acute respiratory syndrome"[All Fields])) OR (("severe acute respiratory syndrome coronavirus 2"[Supplementary Concept] OR "severe acute respiratory syndrome coronavirus 2"[All Fields]) OR "sars cov 2"[All Fields]))

**S1 Table. Baseline characteristics of included RCTs**

|  | **Gordon et al.^20^** | | **Hermine et al.^21^** | | **Rosas et al.^22^** | | **Salama et al.^23^** | | **Salvarani et al.^24^** | | **Stone et al.^25^** | | **Veiga et al.^26^** | | | **Horby et al.^27^** | | **Soin et al.^28^** | |
| --- | --- | --- | --- | --- | --- | --- | --- | --- | --- | --- | --- | --- | --- | --- | --- | --- | --- | --- | --- |
| **Country (ies)** | Multiple^a^ | | France | | USA, UK, Spain | | Multiple^b^ | | Italy | | USA | | Brazil | | | Multiple countries | | India | |
| **COVID-19 diagnosis** | I: 82.3% RT-PCR confirmed SARS-CoV-2  C: 84.8% | | I:89% RT-PCR confirmed SARS-CoV-2  C: 90% | | 100% RT-PCR confirmed SARS-CoV-2 | | 100% RT-PCR confirmed SARS-CoV-2 | | 100% RT-PCR confirmed SARS-CoV-2 | | PCR or serum IgM antibody assay (unspecified %) | | 100% RT-PCR confirmed SARS-CoV-2 | | | 94% RT-PCR confirmed SARS-CoV-2 | | 100% RT-PCR confirmed SARS-CoV-2 | |
| **Severity of hospitalized patients at baseline per WHO classification** | Severe: ICU admission (high-flow O_2_: 28%; non-invasive ventilation (NIV): 42%; invasive MV: 30%) | | Moderate: requiring oxygen by mask or nasal prongs (WHO-CPS scale 5: 100%) | | Moderate to Severe: Supplementary O_2_ (28%); NIV or high flow O_2_ (30%); MV (38%). | | Moderate to severe: No supplementary O_2_ (9%); Supplementary O_2_ (64%); NIV or high-flow O_2_ (27%) | | Moderate to severe: Supplementary O_2_ or high-flow O_2_ (100%) | | Moderate: No supplementary O_2_ (16%); Supplementary O_2_ (80%); NIV or high flow O_2_ (4%) | | Moderate to severe: Supplementary O_2_ (52%), NIV or high-flow O_2_ (15%) or invasive MV (16%) | | | Moderate to severe:  Supplementary O_2_ (45%), NIV or high-flow O_2_ (41%). Invasive MV (14%) | | Moderate to severe: Supplementary O_2_ (90%), non-invasive BiPAP (27%), MV (5%) | |
| **Sample Size** | 895 | | 131 | | 438 | | 389 | | 126 | | 243 | | 129 | | | 4116 | | 180 | |
| **Intervention Group** | TCZ 8mg/kg IV, with possible 2nd dose | | TCZ 8mg/kg on day 1, and 400mg on day 3 | | TCZ 8mg/kg IV, up to 800mg/d | | TCZ 8mg/kg IV, up to 800mg/d | | TCZ 8mg/kg IV, up to 800mg/d, followed by 2^nd^ dose 12 hours later | | TCZ 8mg/kg IV, up to 800mg/d | | TCZ 8mg/kg IV, single dose | | | TCZ 8mg/kg IV, up to 800mg/d, possible 2^nd^ dose 12-24 hours later | | TCZ 6mg/kg IV, up to 480mg/d, possible 2^nd^ dose 12h-7d | |
| **Comparator Group** | SoC | | SoC | | Placebo + SoC | | Placebo + SoC | | SoC | | Placebo + SoC | | SoC | | | SoC | | SoC | |
| **Co-interventions** | Steroids, remdesivir | | HCQ, AZT, steroids, anticoagulants, antivirals, remdesivir, anakinra | | Steroids, antivirals, CP | | Steroids, antivirals | | HCQ, AZT, anticoagulants, antivirals | | HCQ, steroids, antivirals, remdesivir | | HCQ, AZT, steroids, anticoagulants, immunosupressors, vasopressors | | | Steroids, colchicine, IVIG, CP, SNAb, antiplatelet | | Remdesivir, steroids | |
| **Follow-up Time** | 21 days | | 28 days | | 28 days | | 28 days | | 14 days | | 28 days | | 15 days | | | 28 days | | 28 days | |
| **Primary Outcome** | Composite of organ support free-days + death at 21 days | | Clinical worsening at 14 days | | Clinical status at 28 days | | Composite of need of MV + death at 28 days | | Clinical worsening at 14 days | | Composite of intubation + death at 28 days | | Clinical status at 15 days | | | 28-day mortality | | Progression of COVID-19 at 14 days | |
| **Risk of Bias 2.0** | Some concerns | | Low | | Some concerns | | Some concerns | | Some concerns | | Some concerns | | High | | | Low | | Some concerns | |
| **Baseline characteristics** | **I(353)** | **C(402)** | **I (63)** | **C(67)** | **I (294)** | **C(144)** | **I (259)** | **C(129)** | **I (60)** | **C (66)** | **I (161)** | **C(82)** | | **I (65)** | **C(64)** | **I**  **(2022)** | **C (2094)** | **I (91)** | **C (88)** |
| **Age Median (IQR) or Mean (SD)** | 61.5 (12.5) | 1.1 (12.8) | 64 (57.1-74.3) | 63.3 (57.1-72.3) | 60.9 (14.6) | 60.6 (13.7) | 56.0 (14.3) | 55.6 (14.9) | 61.5 (53.5-73.5) | 60 (54-69) | 61.6 (46.4-69.7) | 56.5 (44.7-67.8) | | 57.4 (15.7) | 57.5 (13.5) | 63.3  (13.7) | 63.9 (13.6) | 56 (47-63) | 54 (43-63) |
| **Male Gender (n, %)** | 261 (73.9) | 283 (70.4) | 44 (70) | 44 (66) | 205 (69.7) | 101 (70.1) | 150 (60.2) | 73 (57.0) | 40 (66.7) | 37 (56.1) | 96 (60) | 45 (55) | | 44 (68) | 44 (69) | 1335  (66) | 1437  (69) | 76 (84) | 76 (86) |
| **BMI Median (IQR) or Mean (SD)** | 30.5 (26.9-34.9) | 30.9 (27.1-34.9) | 27.9 (23.3-30.8) | 27.4 (24.5-31.3) | NA | NA | 32 (7.9) | 33.1 (7.2) | NA | NA | 29.9 (26-34.2) | 30.2 (25.7-33.8) | | NA | NA | NA | NA | 27 (4.4) | 26.8 (4.6) |

^a^Multiple: Australia, Belgium, Canada, Ireland, Netherlands, New Zealand, North Ireland, Sri Lanka, Thailand, UK, USA

^b^Multiple: Brazil, Kenya, Mexico, Peru, South Africa, USA

AZT = Azithromycin; C = Control group; HCQ = hydroxychloroquine; I = Intervention group; ICU = intensive care unit; IgM = immunoglobulin M; IQR = interquartile range; IV = intravenous; IVIG = intravenous immunoglobulin; MV = mechanical ventilation; NIV = non-invasive ventilation; PCR = polymerase chain reaction; RT-PCR = real-time polymerase chain reaction; TCZ = tocilizumab; SD = standard deviation; SoC = standard of care; CP = Convalescent plasma; WHO-CPS = World Health Organization 10-point Clinical Progression Scale; BiPAP: Bilevel positive airway pressure.

**S2 Table. Baseline characteristics of included IPTW-cohort studies**

|  | **Biran et al.^29^** | | | **Chilimuri et al.^30^** | | | **Gupta et al.^31^** | | | **Hill et al.^32^** | | | **Martinez-Sanz et al.^33^** | | | **Rodriguez-Baño et al.^34^** | | | **Rossi et al.^35^** | | | **Roumier et al.^36^** | | | **Somers et al.^37^** | | |
| --- | --- | --- | --- | --- | --- | --- | --- | --- | --- | --- | --- | --- | --- | --- | --- | --- | --- | --- | --- | --- | --- | --- | --- | --- | --- | --- | --- |
| **Country (ies)** | USA | | | USA | | | USA | | | USA | | | Spain | | | Spain | | | France | | | France | | | USA | | |
| **COVID-19 diagnosis** | 100% PCR confirmed SARS-CoV-2 | | | 100% PCR confirmed SARS-CoV-2 | | | 100% PCR confirmed SARS-CoV-2 | | | 100% PCR confirmed SARS-CoV-2 | | | 100% PCR confirmed SARS-CoV-2 | | | 100% PCR confirmed SARS-CoV-2 | | | RT-PCR confirmed SARS-CoV-2 or chest CT scan with typical lesions (unspecified %) | | | RT-PCR confirmed SARS-CoV-2 (94%) or chest CT scan with typical lesions | | | 100% PCR confirmed SARS-CoV-2 | | |
| **Severity of hospitalized patients** | Severe: ICU admission, 94% with MV | | | Moderate to severe; intubated were excluded. No more details | | | Severe: ICU admission, 39% with MV | | | Moderate to severe: Supplementary O_2_ (35%); NIV or high flow O_2_ (34%); MV (34%) | | | Unspecified. No details | | | Moderate: Supplementary O_2_ (95%); High-flow O_2_ or NIV (4%) | | | Moderate: High-flow O_2_ or NIV (5%); no MV | | | Moderate: Supplementary O_2_ (100%); neither high-flow O_2_, nor NIV nor MV | | | Severe: ICU admission, 100% with MV | | |
| **Sample Size** | 630 | | | 1225 | | | 3924 | | | 88 | | | 1229 | | | 778 | | | 246 | | | 96 | | | 154 | | |
| **Intervention Group** | TCZ 4-8mg/kg IV, with possible 2^nd^ dose | | | TCZ 400mg IV, single dose | | | TCZ IV or SC, unspecified dose | | | TCZ 400mg/d IV, with 2nd dose on day 3 | | | TCZ 600-800mg/d IV | | | TCZ intermediate-high dose, unspecified | | | TCZ 400mg IV or SC, with possible 2^nd^ dose | | | TCZ 8mg/kg IV, up to 800mg/d, with possible 2^nd^ dose | | | TCZ 8mg/kg IV, up to 800mg/d | | |
| **Comparator Group** | SoC | | | SoC | | | SoC | | | SoC | | | SoC | | | SoC | | | SoC | | | SoC | | | SoC | | |
| **Co-interventions** | HCQ, AZT, steroids | | | HCQ, steroids, anticoagulants, antivirals, antibiotics, CP | | | HCQ, AZT, anticoagulants, steroids | | | HCQ, anticoagulants, remdesivir | | | HCQ, AZT, antivirals, steroids | | | Steroids | | | Steroids, antivirals, antibiotics, baricitinib | | | HCQ, AZT, antivirals, antibiotics, steroids | | | HCQ, anticoagulants, steroids, remdesivir | | |
| **Follow-up Time** | 22 days | | | 28 days | | | 28 days | | | 28 days | | | NA | | | 21 days | | | 28 days | | | 28 days | | | 47 days | | |
| **Primary Outcome** | Death | | | Intubation or death | | | Death | | | Clinical improvement | | | Time to death | | | Intubation or death | | | Composite of MV + death | | | Need for ventilatory support | | | Survival | | |
| **ROBINS-I** | Serious | | | Serious | | | Moderate | | | Moderate | | | Serious | | | Serious | | | Moderate | | | Serious | | | Serious | | |
| **Baseline characteristics** | **I (420)** | **C**  **(210)** | **I**  **(87)** | | **C**  **(1138)** | **I**  **(433)** | | **C**  **(3491)** | **I**  **(43)** | | **C**  **(45)** | **I**  **(260)** | | **C**  **(969)** | **I**  **(88)** | | **C**  **(344)** | **I**  **(106)** | | **C (140)** | **I (49)** | | **C (47)** | **I (78)** | | **C (76)** |  |
| **Age Median (IQR) or Mean (SD)** | 62 (53-71) | 65 (56-74) | 60 (50-70) | | 63 (54-73) | 58 (48-65) | | 63 (52-72) | NA | | NA | 65 (55-76) | | 68 (57-80) | 66 (56-72) | | 69 (59-76) | 64.8 (12.8) | | 64.4 (16.9) | 57.8 (11.5) | | 62.2 (13) | 55 (14.9) | | 60  (14.5) |  |
| **Male Gender (n, %)** | 155 (74) | 281 (67) | 52 (62.7) | | 324  (47.3) | 299 (69.1) | | 2165 (62) | 30 (70) | | 31 (69) | 191 (73) | | 574 (59) | 40 (62.5) | | 237 (69.1) | 55 (65.5) | | 49 (58.3) | 40 (82) | | 38 (81) | 53 (68) | | 49 (64) |  |
| **BMI Median (IQR) or Mean (SD)** | 76 (36) | 154 (37) | NA | | NA | 31.6 (27.5-37) | | 30.4 (26.3-35.9) | NA | | NA | NA | | NA | NA | | NA | NA | | NA | 27.7 (4.4) | | 28.2 (4.4) | 34.7 (10.1) | | 33.4 (8.8) |  |

AZT = azythromycin; C = control group; HCQ = hydroxychloroquine; I = intervention group; ICU, intensive care unit; IPTW = inverse probability treatment weighting; IQR = interquartile range; IV = intravenous; MV = mechanical ventilation; NA = not available; NIV = non-invasive ventilation; PCR = polymerase chain reaction; RT-PCR = real-time polymerase chain reaction; SC = subcutaneous; SD = standard deviation; SoC = standard of care; TCZ = tocilizumab.

**S1 Fig.** Cochrane Risk of Bias 2.0 Tool of included randomized controlled trials

**S2 Fig**. ROBINS-I Risk of Bias Tool Figure of included cohort studies


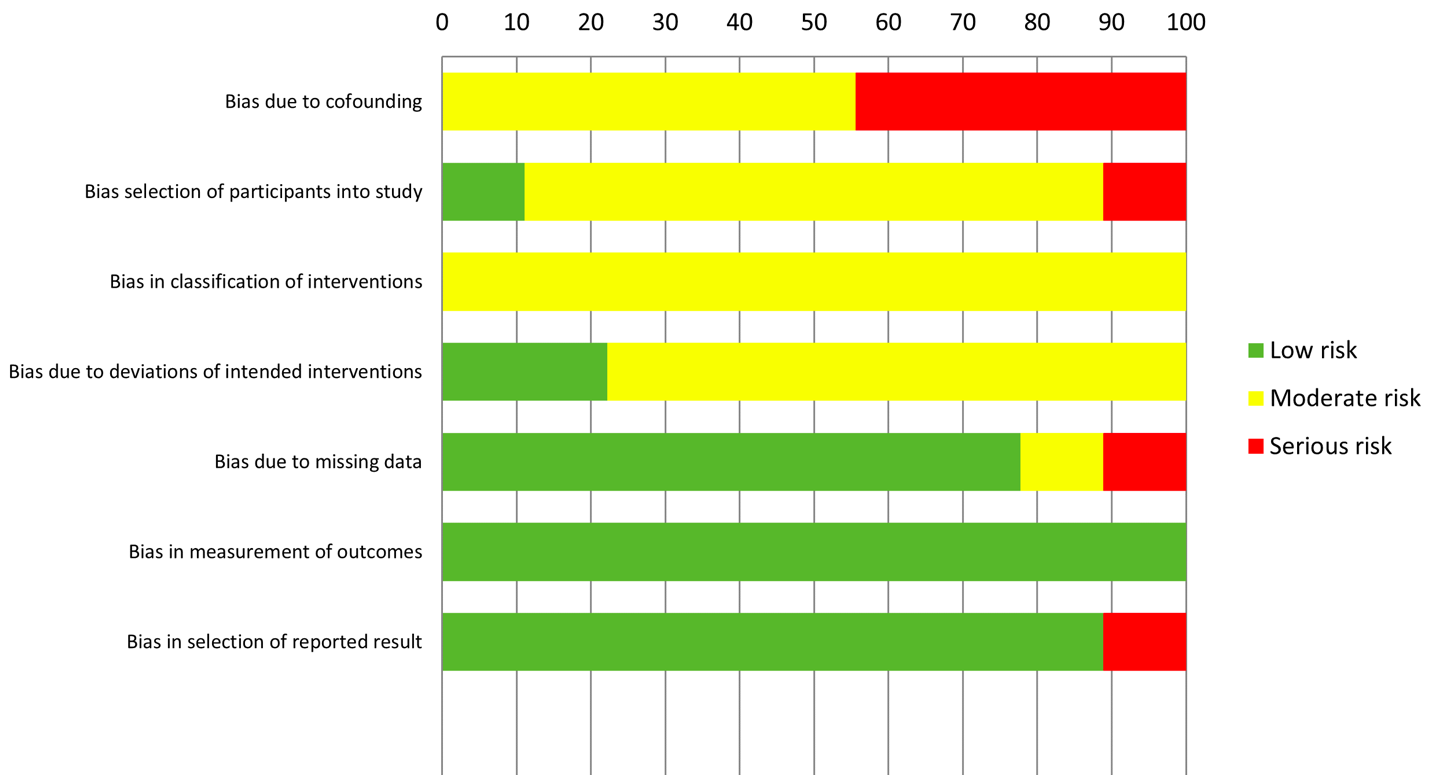


**S3 Fig.** Effect of tocilizumab on clinical improvement in RCTs and IPTW cohorts of hospitalized COVID-19 patients.


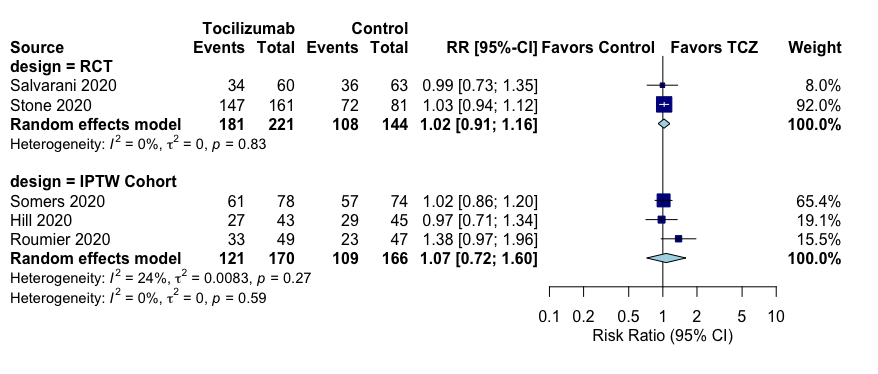


**S4 Fig.** Effect of tocilizumab on clinical worsening in RCTs and IPTW cohorts of hospitalized COVID-19 patients.


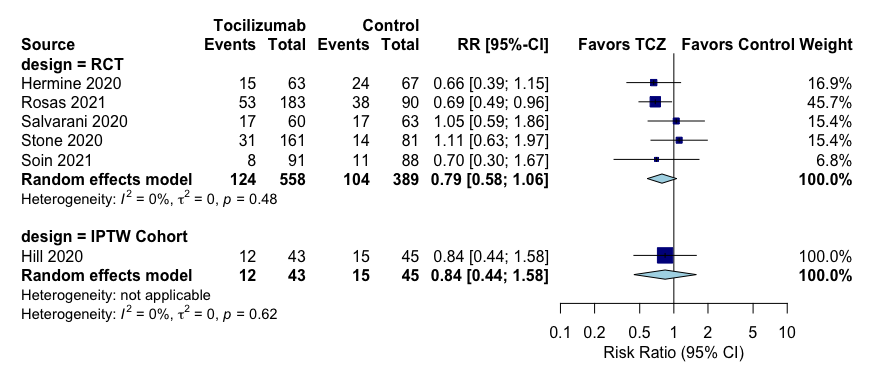


**S5 Fig.** Effect of tocilizumab on length of hospital stay in RCTs and IPTW cohorts of hospitalized COVID-19 patients.


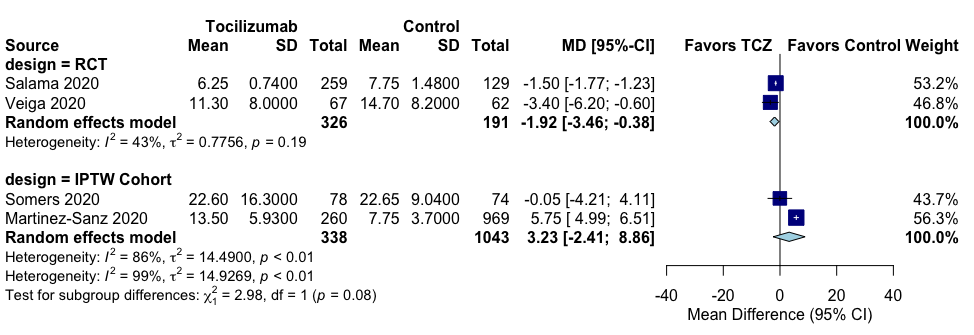


**S6 Fig.** Effect of tocilizumab on severe adverse events in RCTs of hospitalized COVID-19 patients.


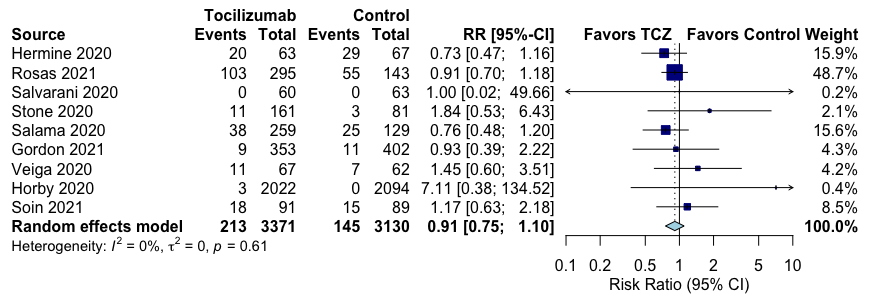


**S7 Fig.** Effect of tocilizumab on bacteremia/infection in RCTs and IPTW cohorts of hospitalized COVID-19 patients.


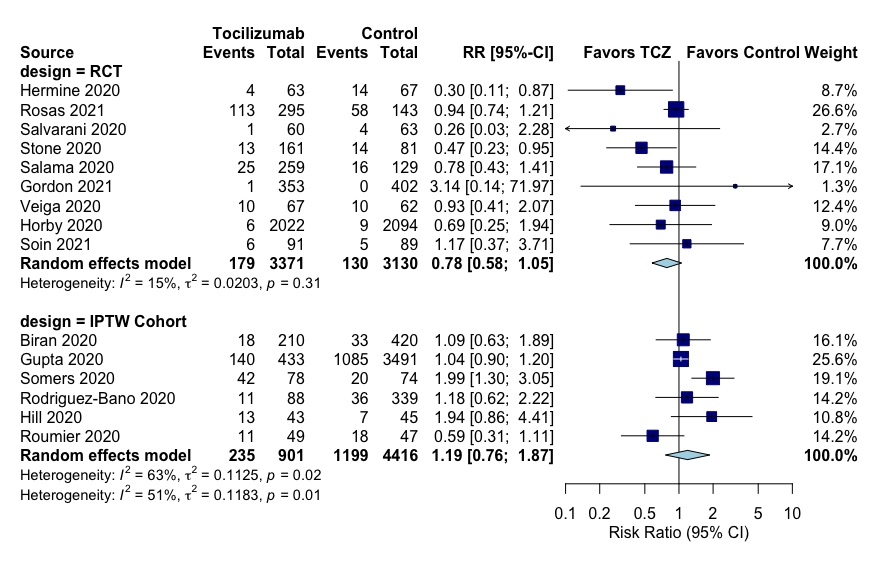


**S8 Fig.** Effect of tocilizumab on neutropenia in RCTs and IPTW cohorts of hospitalized COVID-19 patients.


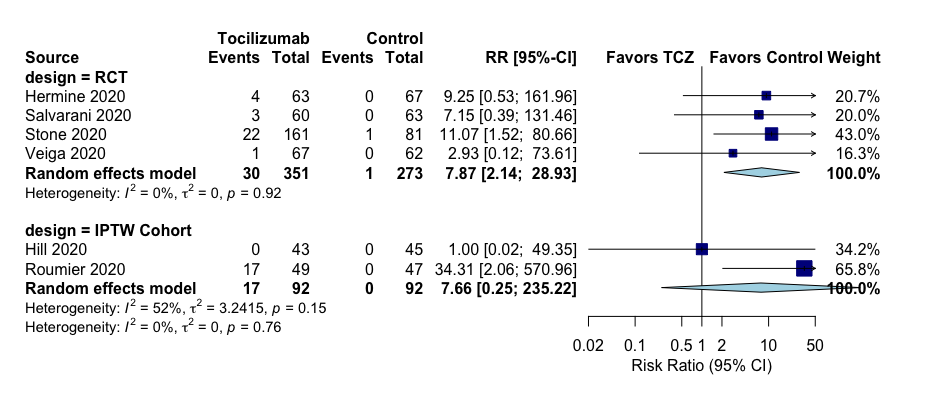


**S9 Fig.** Effect of tocilizumab on bleeding events in RCTs and IPTW cohorts of hospitalized COVID-19 patients.


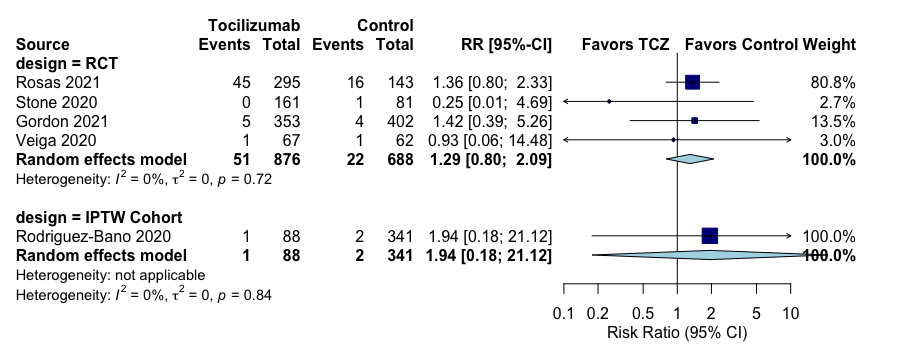


**S10 Fig**. Effect of tocilizumab on thrombotic events in RCTs and IPTW cohorts of hospitalized COVID-19 patients.


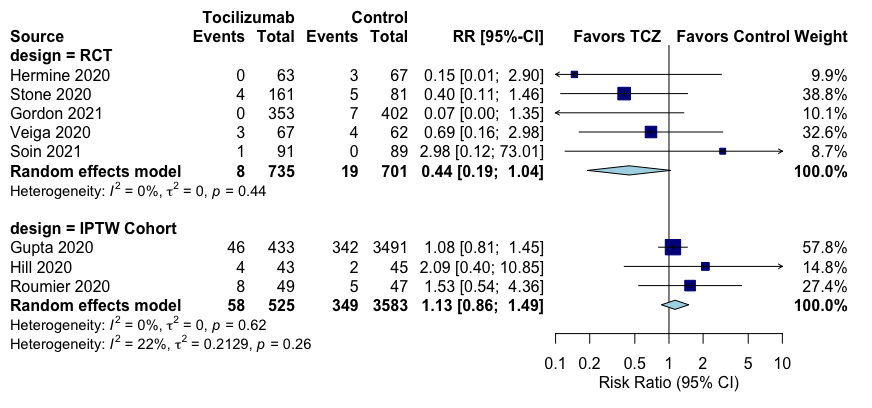


**S11 Fig.** Effect of tocilizumab on abnormal liver function in RCTs and IPTW cohorts of hospitalized COVID-19 patients.

**
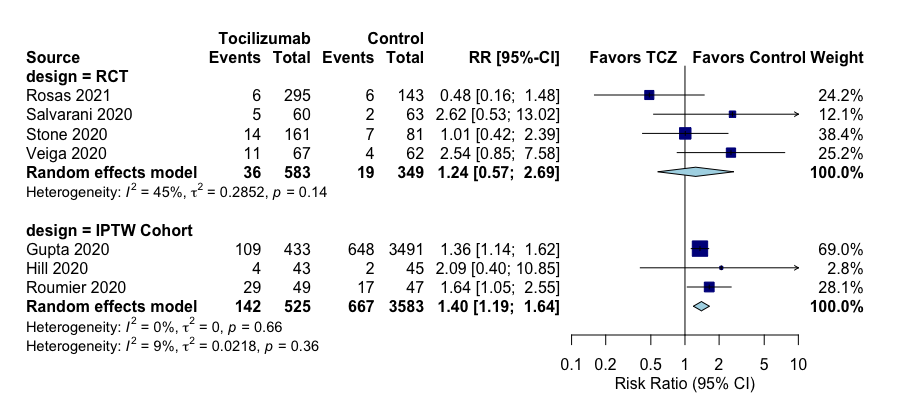
**

**S12 Fig. Subgroup analyses**

**S12.1. All-cause mortality**

**S12.1.1. RCTs**

**S12.1.1.a. Severity:**


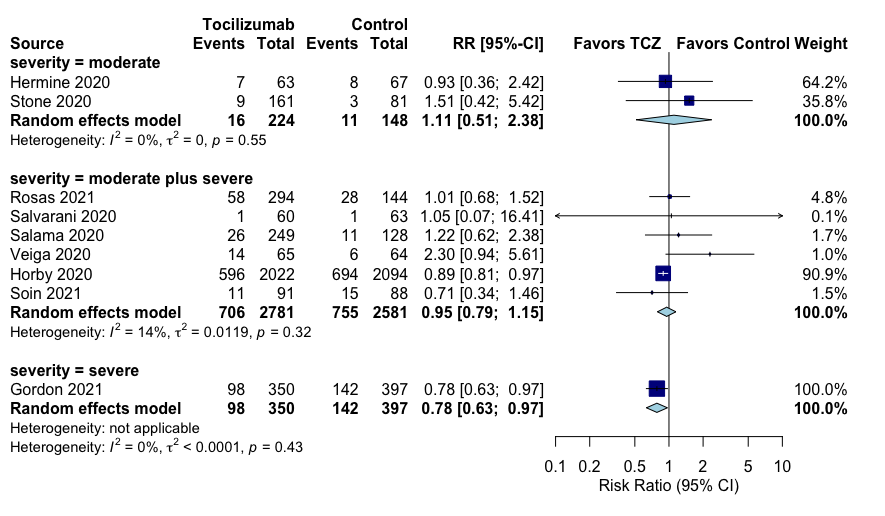


**S12.1.1.b. RoB:**


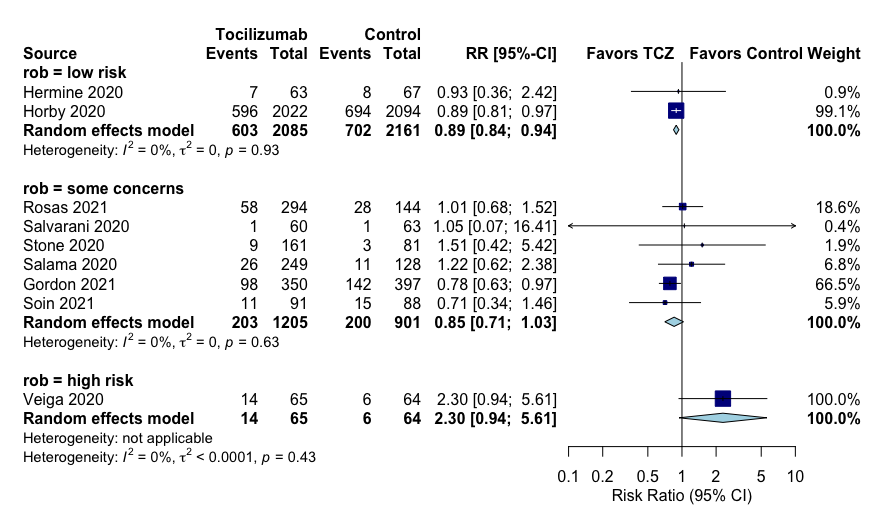


**S12.1.2. IPTW cohorts**

**S12.1.2.a. Severity:**


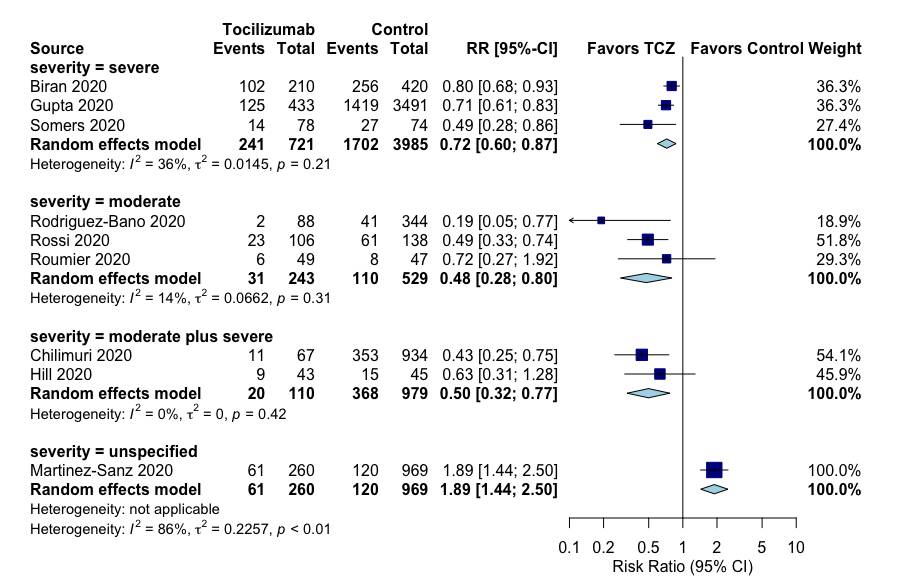


**S12.1.2.b. RoB:**

**
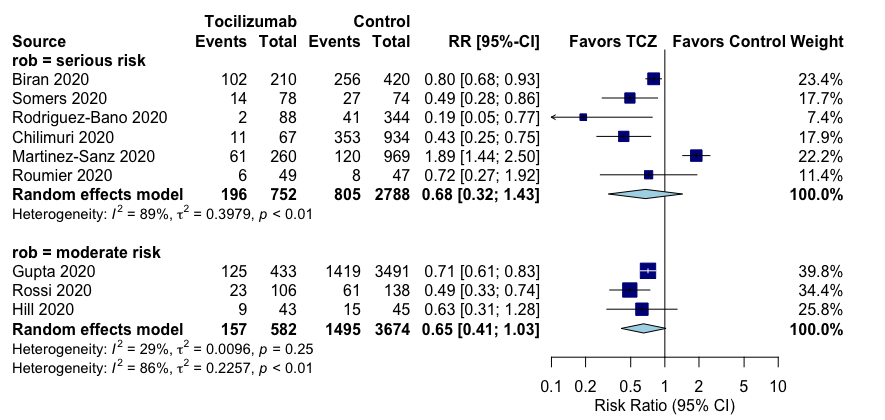
**

**S12.2. Clinical improvement**

**S12.2.1. RCTs**

**S12.2.1.a. Severity:**

**
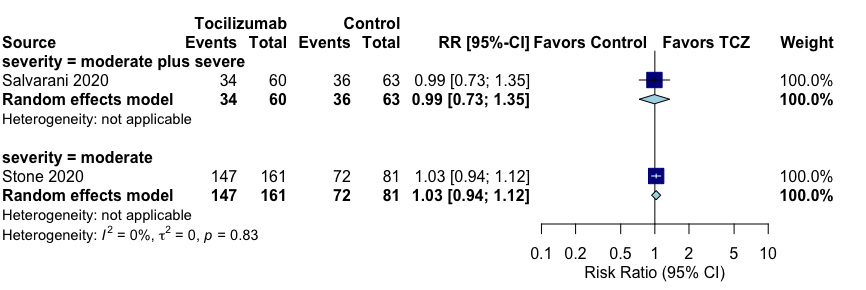
**

**S12.2.1.b. RoB: N/A**

**S.12.2. IPTW cohorts**

**S12.2.2.a. Severity:**

**
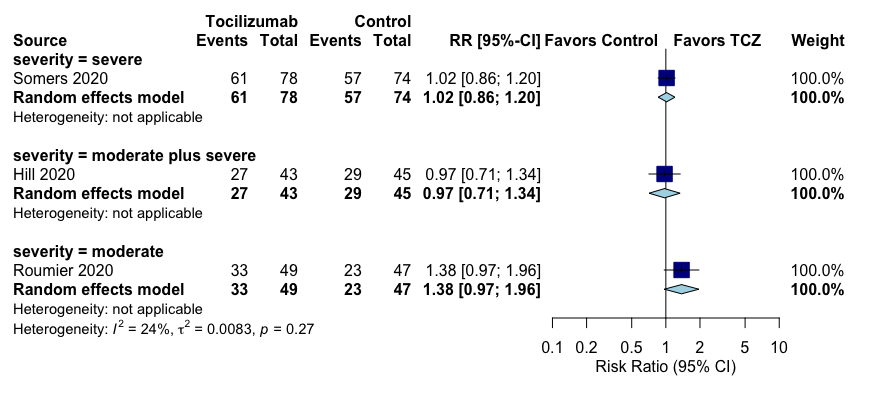
**

**S12.2.2.b. RoB:**

**
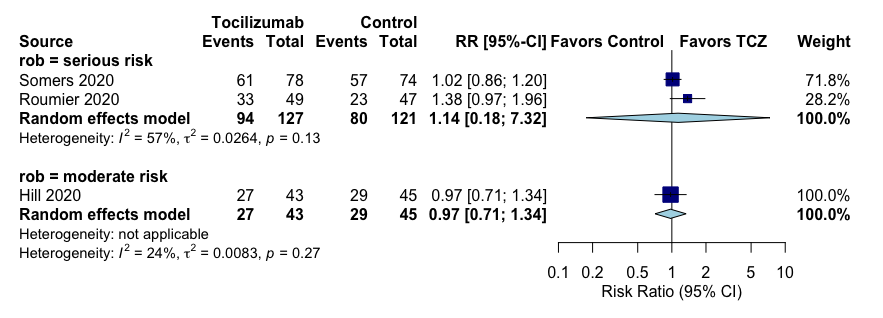
**

**S12.3. Clinical worsening**

**S12.3.1. RCTs**

**S12.3.1.a. Severity:**

**
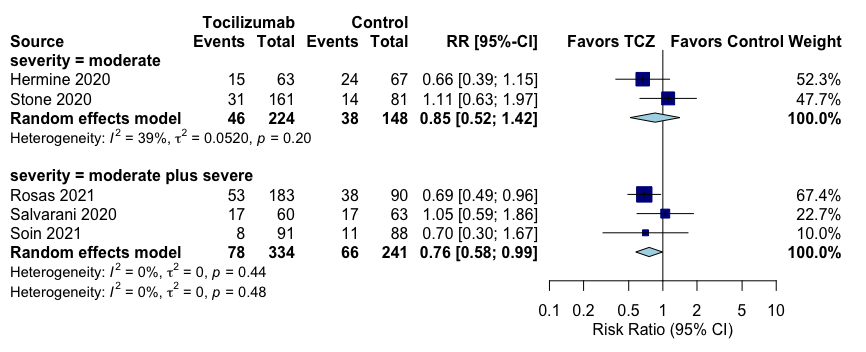
**

**S12.3.1.b. RoB:**

**
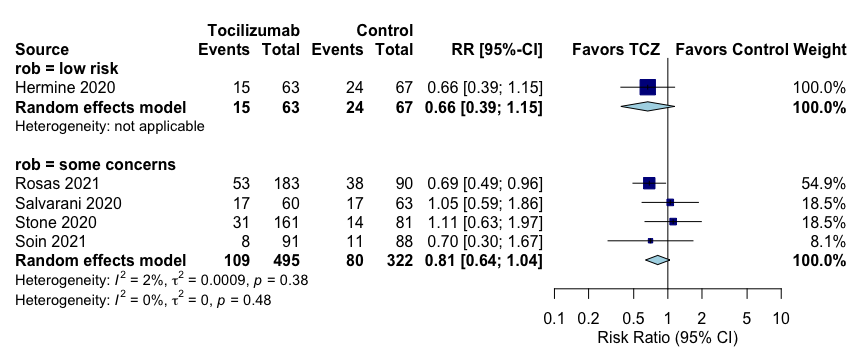
**

**S12.3.2. IPTW cohorts**

**S12.3.2.a. Severity:** N/A

**S12.3.2.b. RoB:** N/A

**S12.4. Mechanical ventilation**

**S12.4.1. RCTs**

**S12.4.1.a. Severity:**

**
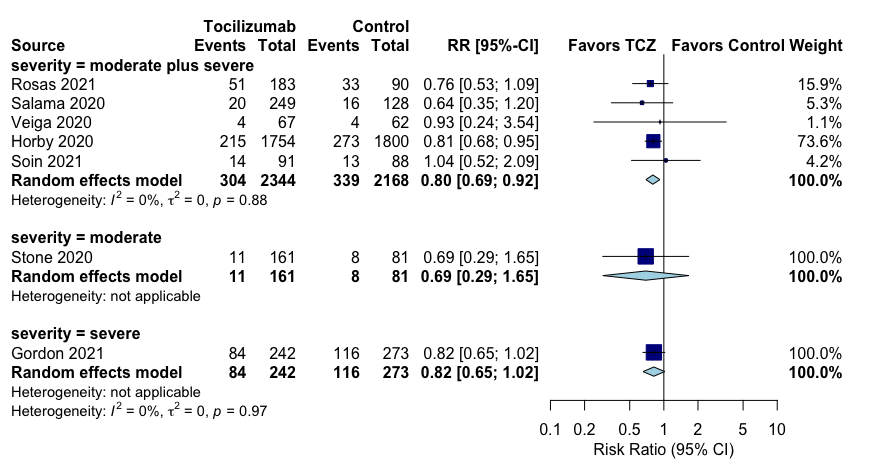
**

**S12.4.1.b. RoB:**

**
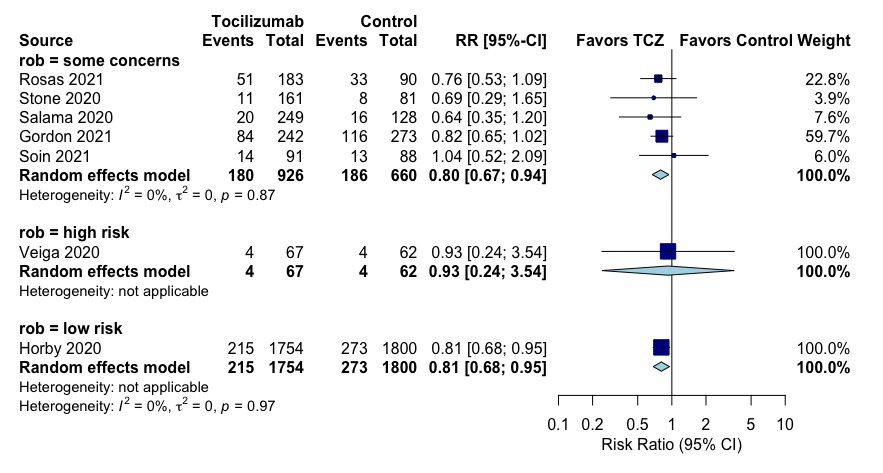
**

**S12.4.2. IPTW cohorts**

**S12.4.2.a. Severity:**

**
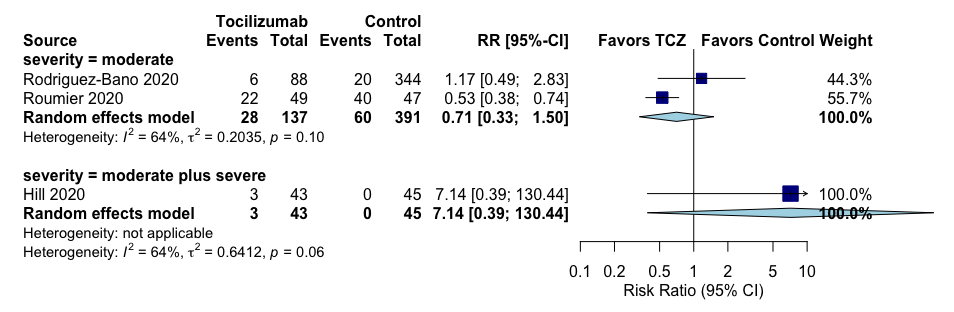
**

**S12.4.2.b. RoB:**

**
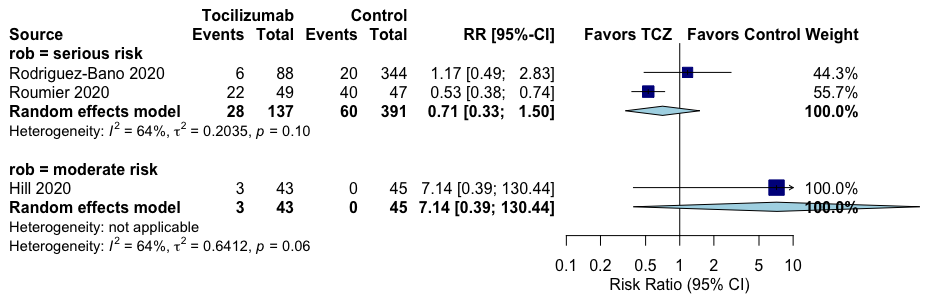
**

**S12.5. Adverse events**

**S12.5.1. RCTs**

**S12.5.1.a. Severity:**

**
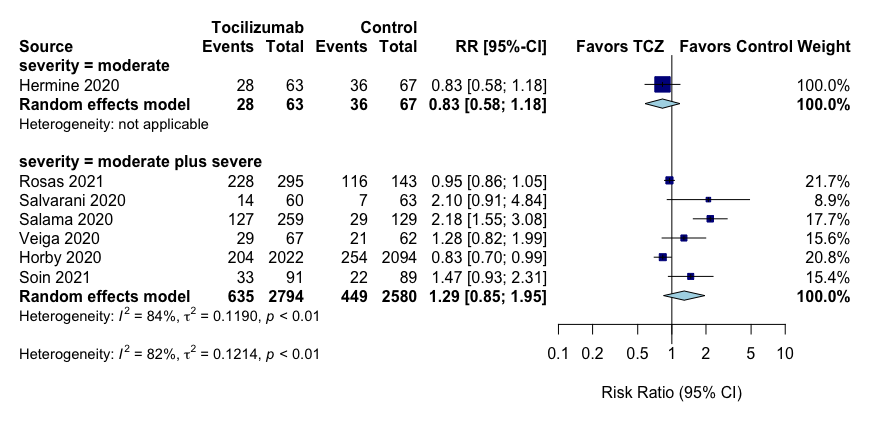
**

**S12.5.1.b. RoB:**

**
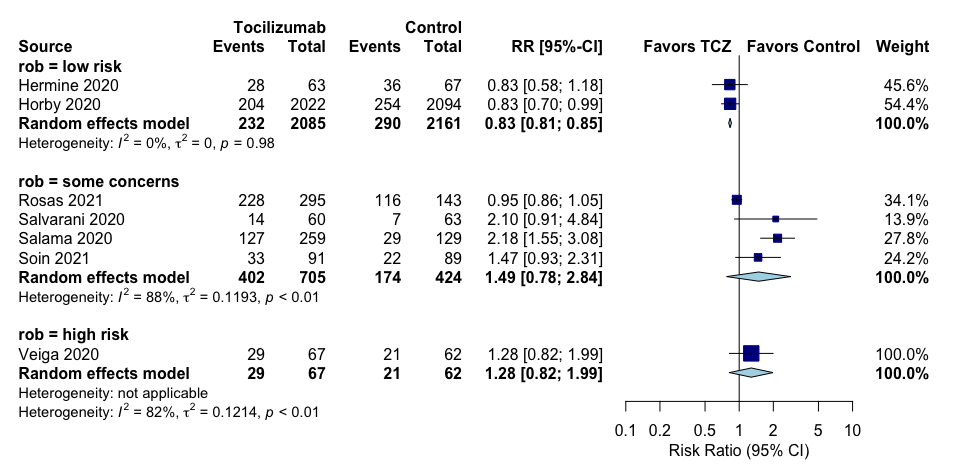
**

**S12.6. Severe adverse events**

**S12.6.1. RCTs**

**S12.6.1.a. Severity:**

**
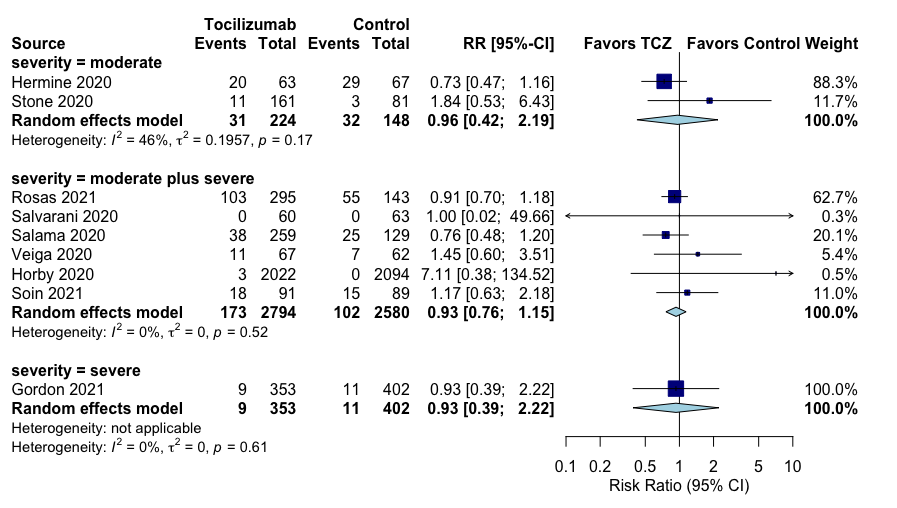
**

**S12.6.1.b. RoB:**

**
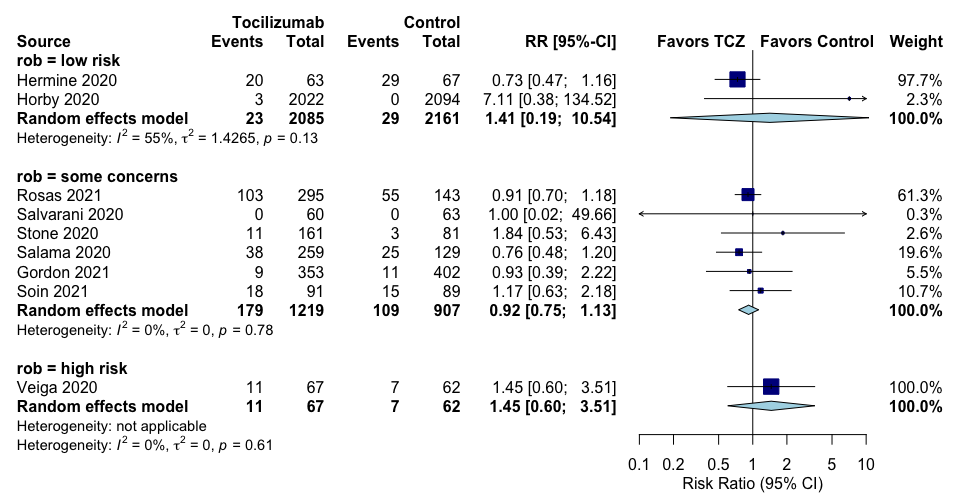
**

**S12.7. Bacteremia/infection**

**S12.7.1. RCTs**

**S12.7.1.a. Severity:**

**
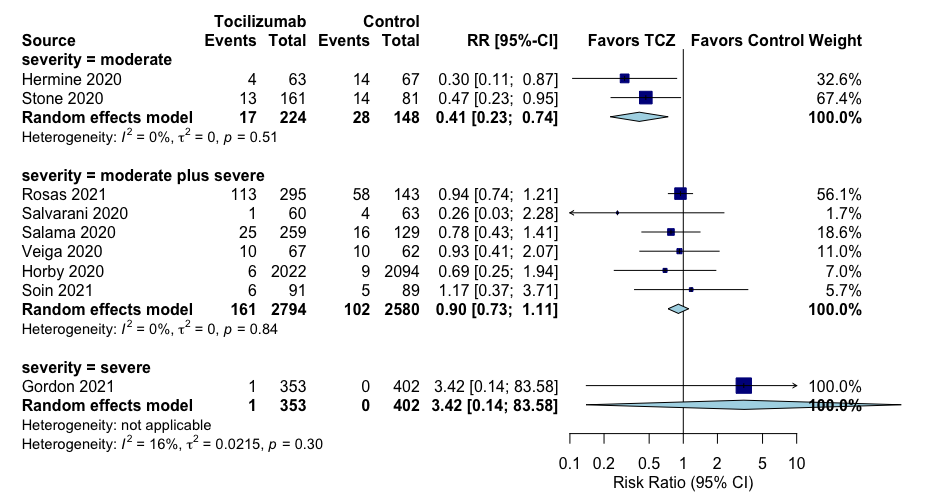
**

**S12.7.1.b. RoB:**

**
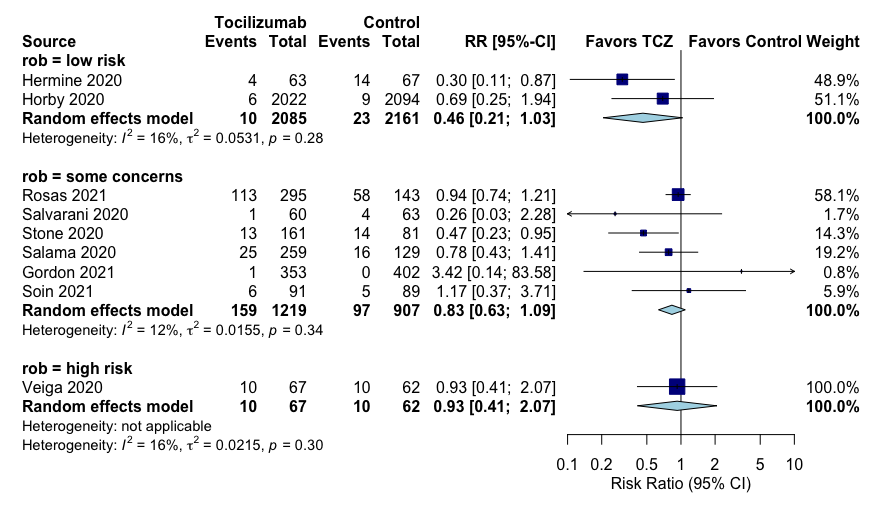
**

**S12.7.2. IPTW cohorts**

**S12.7.2.a. Severity**

**
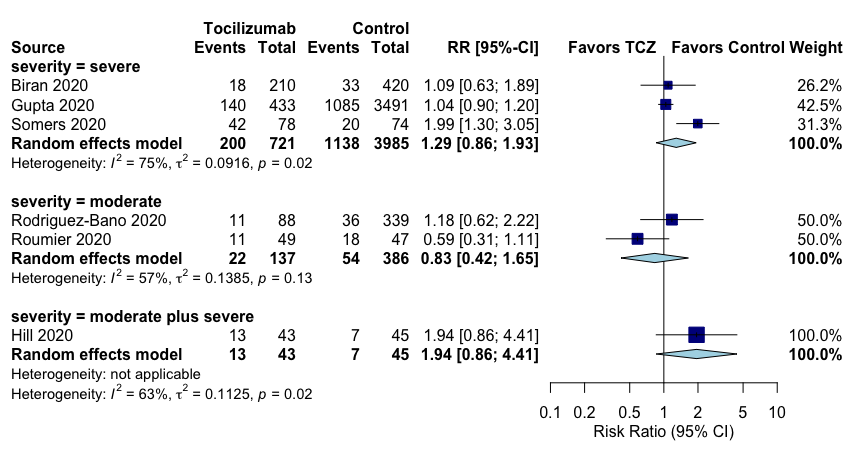
**

**S12.7.2.b. RoB**

**
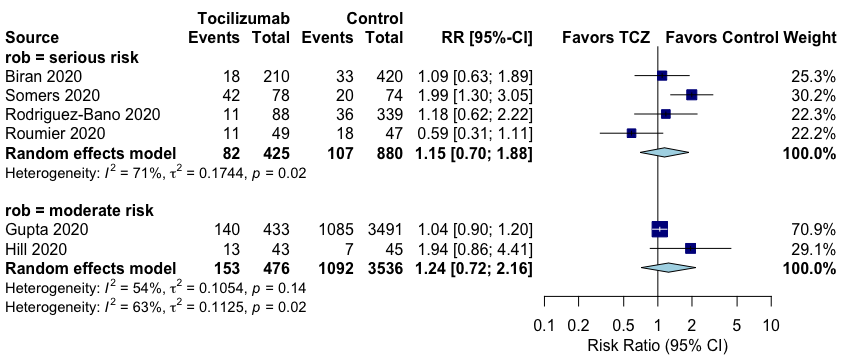
**

**S12.8. Bleeding events**

**S12.8.1. RCTs**

**S12.8.1.a. Severity:**

**
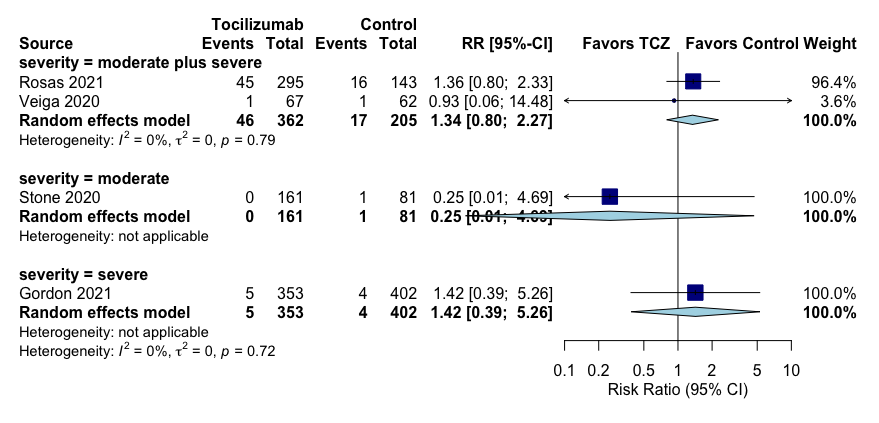
**

**S12.8.1.b. RoB:**


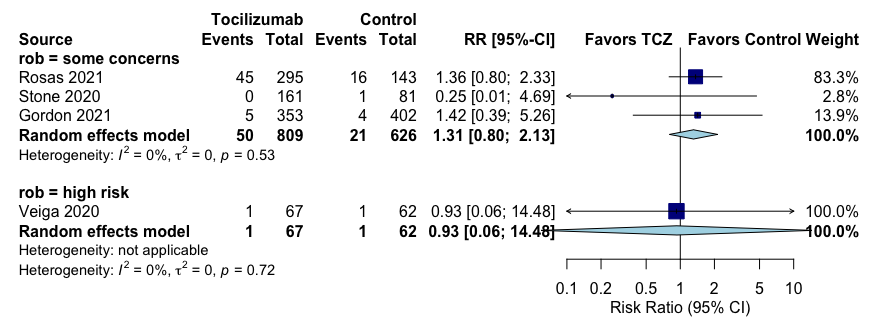


**S12.8.2. IPTW cohorts**

**S12.8.2.a. Severity:** N/A

**S12.8.2.b. RoB:** N/A

**S12.9. Neutropenia**

**S12.9.1. RCTs**

**S12.9.1.a. Severity:**

**
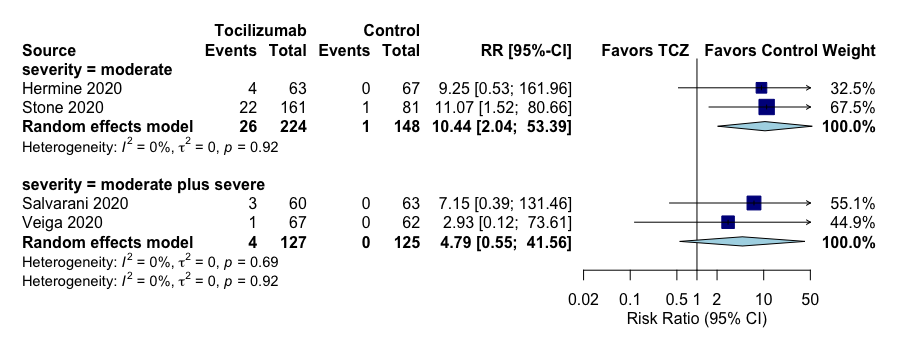
**

**S12.9.1.b. RoB:**

**
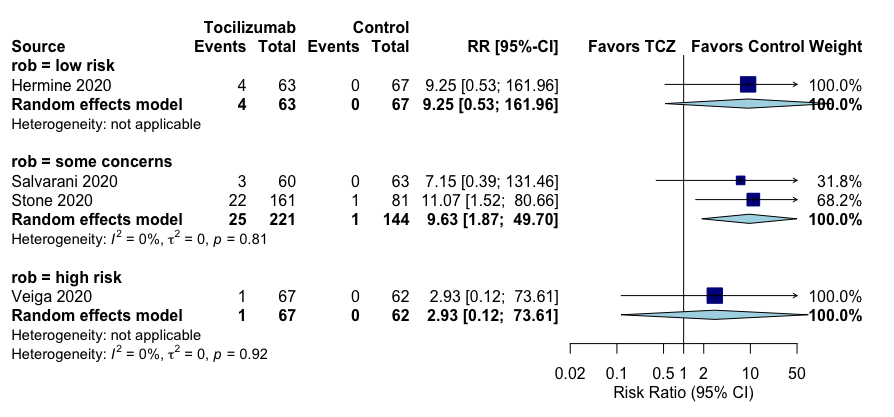
**

**S12.9.2. IPTW cohorts**

**S12.9.2.a. Severity:** N/A

**S12.9.2.b. RoB:** N/A

**S12.10. Thrombotic Events**

**S12.10.1. RCTs**

**S12.10.1.a. Severity:**

**
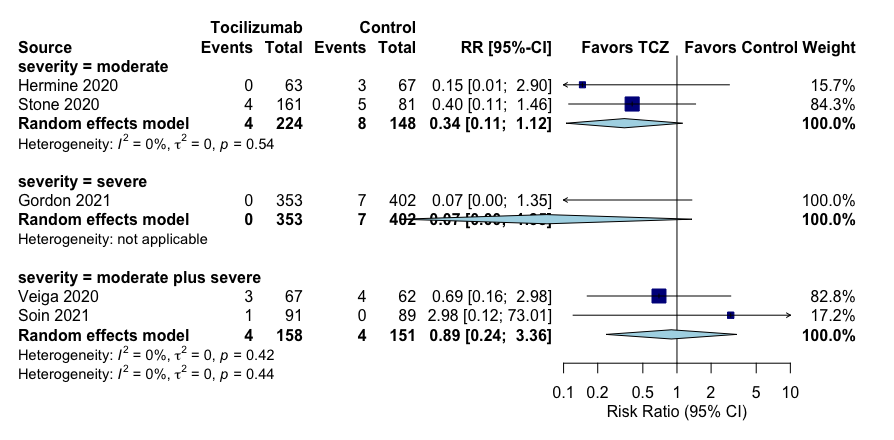
**

**S12.10.1.b. RoB:**

**
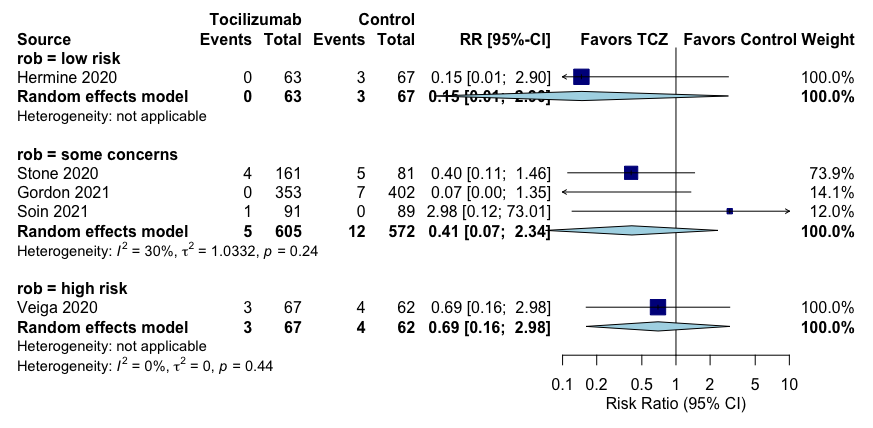
**

**S12.10.2. IPTW cohorts**

**S12.10.2.a. Severity:**

**
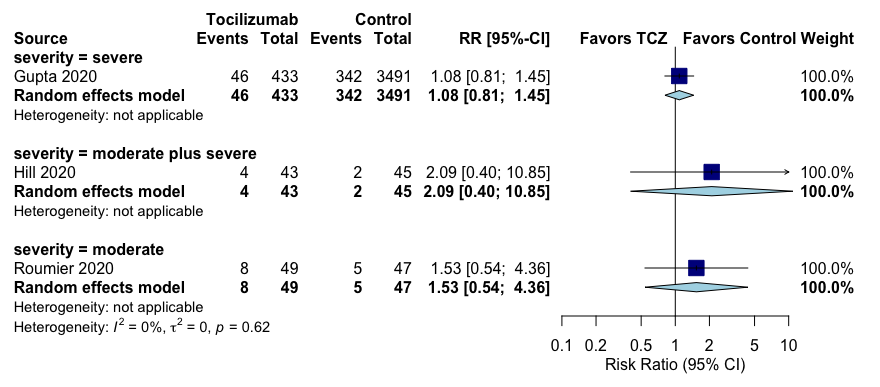
**

**S12.10.2.b. RoB:**

**
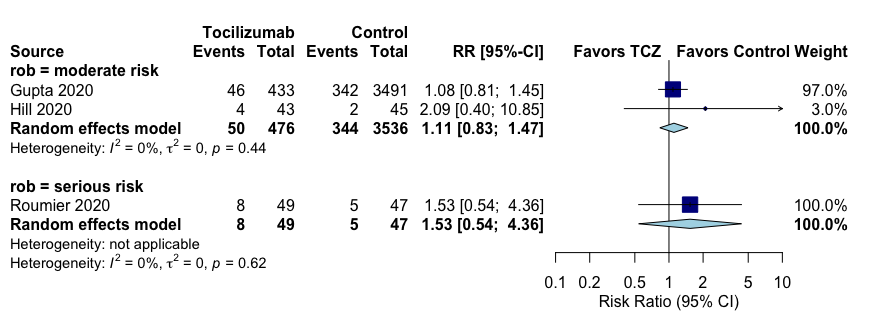
**

**S12.11. Abnormal liver function**

**S12.11.1. RCTs**

**S12.11.1.a. Severity:**

**
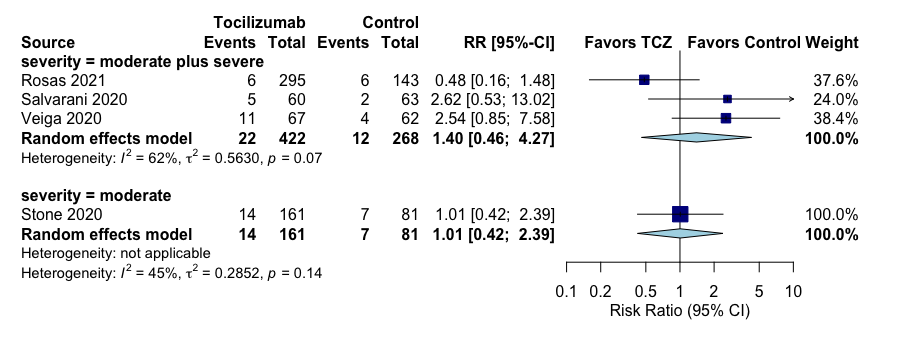
**

**S12.11.1.b. RoB:**

**
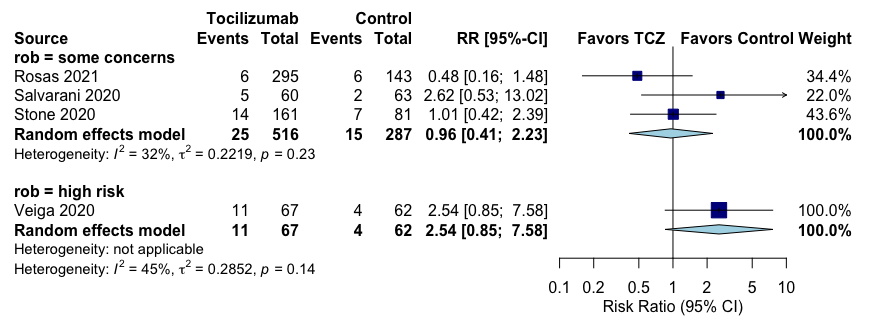
**

**S12.11.2. IPTW Cohorts**

**S12.11.2.a. Severity:**

**
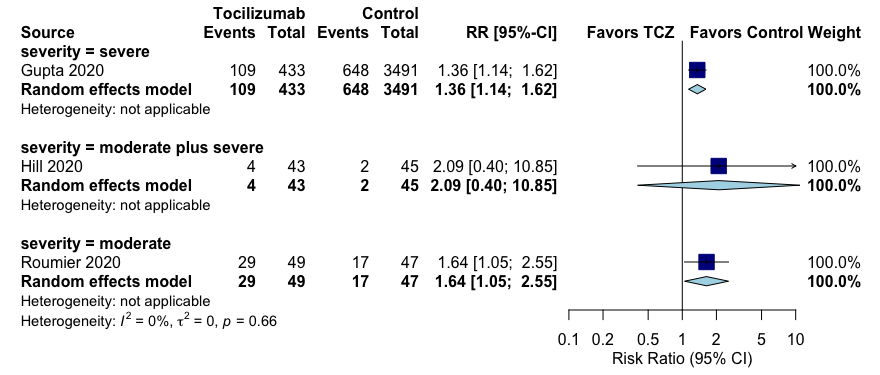
**

**S12.11.2.b. RoB:**

**
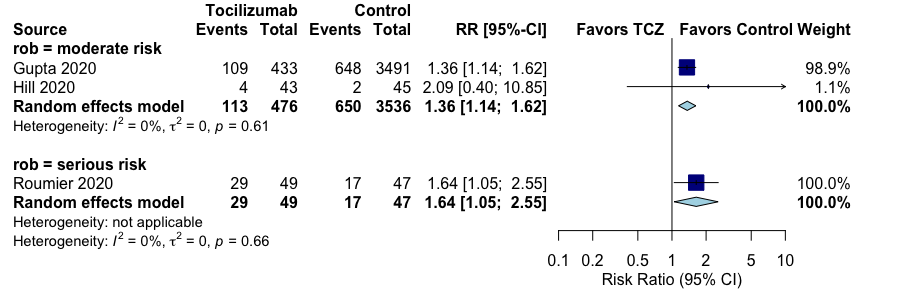
**
