# Supplementary material for: Efficacy and harms of tocilizumab for the treatment of COVID-19 patients: A systematic review and meta-analysis
Source: PLoS One. 2022 Jun 3;17(6):e0269368. doi: 10.1371/journal.pone.0269368 (PMC9165853; doi:10.1371/journal.pone.0269368)
Supplement: S3 File — (DOCX) [file pone.0269368.s003.docx]

The Risk Of Bias In Non-randomized Studies – of Interventions (ROBINS-I) assessment tool

(version for cohort-type studies)

**Version 19 September 2016**


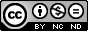


This work is licensed under a [Creative Commons Attribution-NonCommercial-NoDerivatives 4.0 International License](http://creativecommons.org/licenses/by-nc-nd/4.0/).

# ROBINS-I tool (Stage I): At protocol stage

## Specify the review question

| Participants | Hospitalized, adult patients with moderate or severe PCR-confirmed SARS-CoV-2 infection |
| --- | --- |
| Experimental intervention | Tocilizumab + Standard of care |
| Comparator | Standard of care |
| Outcomes | Primary outcomes: mortality rate, intubation rate, clinical improvement |

## List the confounding domains relevant to all or most studies

| Demographics (age, gender, comorbidities)  Baseline inflammatory markers (CRP, D-Dimer, LDH, Ferritin, IL-6)  Baseline severity of disease |
| --- |

## List co-interventions that could be different between intervention groups and that could impact on outcomes

| HCQ, antivirals (remdesivir) and antiretrovirals, antibiotics, convalescent plasma, steroids, anticoagulants |
| --- |

# ROBINS-I tool (Stage II): For each study

**Chilimuri S, Sun H, Alemam A, et al. Tocilizumab use in patients with moderate to severe COVID-19: a retrospective cohort study. J Clin Pharm Ther. 2020. 00:1-7**

## Specify a target randomized trial specific to the study

| Design | Individually randomized / Cluster randomized / **Matched (e.g. cross-over)** |
| --- | --- |
| Participants | Hospitalized, adult patients ≥19 years old, with moderate to severe PCR-confirmed SARS-CoV-2 infection |
| Experimental intervention | Tocilizumab 4-8mg/kg, with suggested dose of 400mg IV, and a second dose if inadequate clinical response |
| Comparator | Standard of care (HCQ, steroids, anticoagulation, antimicrobials, antiretrovirals) |

## Is your aim for this study…?

| **X** | **to assess the effect of *assignment to* intervention** |
| --- | --- |
| □ | to assess the effect of *starting and adhering to* intervention |

## Specify the outcome

Specify which outcome is being assessed for risk of bias (typically from among those earmarked for the Summary of Findings table). Specify whether this is a proposed benefit or harm of intervention.

| Respiratory failure (Intubation or Death), or Time to Intubation if death occurred after |
| --- |

## Specify the numerical result being assessed

In case of multiple alternative analyses being presented, specify the numeric result (e.g. RR = 1.52 (95% CI 0.83 to 2.77) and/or a reference (e.g. to a table, figure or paragraph) that uniquely defines the result being assessed.

| IPTW 🡪 HR: 0.40 (95%CI 0.20-0.77)  No IPTW 🡪 HR: 0.36 (95%CI 0.19-0.71) |
| --- |

## Preliminary consideration of confounders

Complete a row for each important confounding domain (i) listed in the review protocol; and (ii) relevant to the setting of this particular study, or which the study authors identified as potentially important.

#### “Important” confounding domains are those for which, in the context of this study, adjustment is expected to lead to a clinically important change in the estimated effect of the intervention. “Validity” refers to whether the confounding variable or variables fully measure the domain, while “reliability” refers to the precision of the measurement (more measurement error means less reliability).

| **(i) Confounding domains listed in the review protocol** | | | | |
| --- | --- | --- | --- | --- |
| Confounding domain | Measured variable(s) | Is there evidence that controlling for this variable was unnecessary?* | Is the confounding domain measured validly and reliably by this variable (or these variables)? | OPTIONAL: Is failure to adjust for this variable (alone) expected to favour the experimental intervention or the comparator? |
| There are no confounding domains described in the protocol. |  |  |  |  |

| **(ii) Additional confounding domains relevant to the setting of this particular study, or which the study authors identified as important** | | | | |
| --- | --- | --- | --- | --- |
| Confounding domain | Measured variable(s) | Is there evidence that controlling for this variable was unnecessary?* | Is the confounding domain measured validly and reliably by this variable (or these variables)? | OPTIONAL: Is failure to adjust for this variable (alone) expected to favour the experimental intervention or the comparator? |
| Demographics | Age, male gender, BMI, hypertension, diabetes, CV disease, pulmonary disease | There is no evidence controlling was unnecessary | **Yes** / No / No information | Favour experimental / Favour comparator / **No information** |
| Baseline inflammatory markers | CRP, D-Dimer, IL-6, ferritin, LDH | There is no evidence controlling was unnecessary | **Yes** / No / No information | Favour experimental / Favour comparator / **No information** |
| Baseline severity of disease | None | There is no evidence controlling was unnecessary | Yes / **No** / No information | Favour experimental / Favour comparator / **No information** |

* In the context of a particular study, variables can be demonstrated not to be confounders and so not included in the analysis: (a) if they are not predictive of the outcome; (b) if they are not predictive of intervention; or (c) because adjustment makes no or minimal difference to the estimated effect of the primary parameter. Note that “no statistically significant association” is not the same as “not predictive”.

## Preliminary consideration of co-interventions

Complete a row for each important co-intervention (i) listed in the review protocol; and (ii) relevant to the setting of this particular study, or which the study authors identified as important.

#### “Important” co-interventions are those for which, in the context of this study, adjustment is expected to lead to a clinically important change in the estimated effect of the intervention.

| **(i) Co-interventions listed in the review protocol** | | |
| --- | --- | --- |
| Co-intervention | Is there evidence that controlling for this co-intervention was unnecessary (e.g. because it was not administered)? | Is presence of this co-intervention likely to favour outcomes in the experimental intervention or the comparator |
| There are no co-interventions described in the protocol. |  |  |

| **(ii) Additional co-interventions relevant to the setting of this particular study, or which the study authors identified as important** | | |
| --- | --- | --- |
| Co-intervention | Is there evidence that controlling for this co-intervention was unnecessary (e.g. because it was not administered)? | Is presence of this co-intervention likely to favour outcomes in the experimental intervention or the comparator |
| Antiretrovirals | There is no evidence controlling for co-intervention was unnecessary. | Favour experimental / Favour comparator / **No information** |
| Antimicrobials | There is no evidence controlling for co-intervention was unnecessary. | Favour experimental / Favour comparator / **No information** |
| Steroids | There is no evidence controlling for co-intervention was unnecessary. | Favour experimental / Favour comparator / **No information** |
| Anticoagulants | There is no evidence controlling for co-intervention was unnecessary. | Favour experimental / Favour comparator / **No information** |
| Convalescent plasma | There is no evidence controlling for co-intervention was unnecessary. | Favour experimental / Favour comparator / **No information** |

## Risk of bias assessment

Responses underlined in green are potential markers for low risk of bias, and responses in red are potential markers for a risk of bias. Where questions relate only to sign posts to other questions, no formatting is used.

|  | **Signalling questions** | **Description** | **Response options** |
| --- | --- | --- | --- |
| **Bias due to confounding** | | | |
|  | 1.1 Is there potential for confounding of the effect of intervention in this study?  **If N/PN to 1.1:** the study can be considered to be at low risk of bias due to confounding and no further signalling questions need be considered | Yes. There are potential confounders of the effect of intervention. | **Y** / PY / PN / N |
|  | **If Y/PY to 1.1**: determine whether there is a need to assess time-varying confounding: |  |  |
|  | 1.2. Was the analysis based on splitting participants’ follow up time according to intervention received?  **If N/PN**, answer questions relating to baseline confounding (1.4 to 1.6)  **If Y/PY**, go to question 1.3. | No. The intervention received did not change over time and post-baseline prognostic factors could not have affected the effect of intervention. | NA / Y / PY / PN / **N** / NI |
|  | 1.3. Were intervention discontinuations or switches likely to be related to factors that are prognostic for the outcome?  **If N/PN**, answer questions relating to baseline confounding (1.4 to 1.6)  **If Y/PY**, answer questions relating to both baseline and time-varying confounding (1.7 and 1.8) |  | NA / Y / PY / PN / N / NI |

|  | **Questions relating to baseline confounding only** | | |
| --- | --- | --- | --- |
|  | 1.4. Did the authors use an appropriate analysis method that controlled for all the important confounding domains? | Yes. The authors used inverse probability weighting, Kaplan-Meier curves and Cox regression models to control the confounding domains. | NA / **Y** / PY / PN / N / NI |
|  | 1.5. **If Y/PY to 1.4**: Were confounding domains that were controlled for measured validly and reliably by the variables available in this study? | No. The authors measured demographic factors, clinical factors, laboratory tests, radiological tests, and medications validly and reliably to control confounding domains; but they did not measured clinical status at baseline to control for baseline severity of disease. | NA / Y / PY / PN / **N** / NI |
|  | 1.6. Did the authors control for any post-intervention variables that could have been affected by the intervention? | No. The authors did not control any post-baseline variables that could have affected the effect of intervention. | NA / Y / PY / PN / **N** / NI |
|  | **Questions relating to baseline and time-varying confounding** | |  |
|  | 1.7. Did the authors use an appropriate analysis method that controlled for all the important confounding domains and for time-varying confounding? | Yes. The authors used inverse probability weighting, Kaplan-Meier curves and Cox regression models to control the confounding domains and time-varying confounding. | NA / **Y** / PY / PN / N / NI |
|  | 1.8. **If Y/PY to 1.7**: Were confounding domains that were controlled for measured validly and reliably by the variables available in this study? | Yes. The authors measured demographic factors, clinical factors, laboratory tests, radiological tests, and medications validly and reliably to control confounding domains; but they did not measured clinical status at baseline to control for baseline severity of disease. | NA / **Y** / PY / PN / N / NI |
|  | **Risk of bias judgement** | **Serious risk of bias:** i) At least one known important domain was not appropriately measured, or not controlled for. | Low / Moderate / **Serious** / Critical / NI |
|  | Optional: What is the predicted direction of bias due to confounding? | Unpredictable | Favours experimental / Favours comparator / **Unpredictable** |

| **Bias in selection of participants into the study** | | | |
| --- | --- | --- | --- |
|  | 2.1. Was selection of participants into the study (or into the analysis) based on participant characteristics observed after the start of intervention?  **If N/PN to 2.1:** go to 2.4 | No. The participants selected had a baseline study as 24 hours after presentation to the Emergency Department. | Y / PY / PN / **N** / NI |
|  | 2.2. **If Y/PY to 2.1**: Were the post-intervention variables that influenced selection likely to be associated with intervention?  2.3 **If Y/PY to 2.2**: Were the post-intervention variables that influenced selection likely to be influenced by the outcome or a cause of the outcome? |  | NA / Y / PY / PN / N / NI  NA / Y / PY / PN / N / NI |
|  | 2.4. Do start of follow-up and start of intervention coincide for most participants? | No. The start of intervention was defined as administration of the intervention at baseline study (24 hours after presentation to the ED) or anytime during the follow-up period before intubation or death. | Y / PY / PN / **N** / NI |
|  | 2.5. **If Y/PY to 2.2 and 2.3, or N/PN to 2.4**: Were adjustment techniques used that are likely to correct for the presence of selection biases? | Yes. The authors used inverse probability weighting to correct for the presence of selection biases. | NA / **Y** / PY / PN / N / NI |
|  | **Risk of bias judgement** | **Moderate risk of bias:** ii) Start of the follow-up and start of the intervention do not coincide for all participants AND a) The authors used appropriate methods to adjust for the selection bias. | Low / **Moderate** / Serious / Critical / NI |
|  | Optional: What is the predicted direction of bias due to selection of participants into the study? | Unpredictable | Favours experimental / Favours comparator / Towards null /Away from null / **Unpredictable** |

| **Bias in classification of interventions** | | | |
| --- | --- | --- | --- |
|  | 3.1 Were intervention groups clearly defined? | Yes. The intervention and control groups were clearly defined. | **Y** / PY / PN / N / NI |
|  | 3.2 Was the information used to define intervention groups recorded at the start of the intervention? | Yes. The study was conducted at BronxCare Health System (BCHS) in New York City, NY. They use Electronic Medical Records (EMR) to record the start of the intervention. | **Y** / PY / PN / N / NI |
|  | 3.3 Could classification of intervention status have been affected by knowledge of the outcome or risk of the outcome? | Probably no. Classification of the intervention status was done retrospectively according to EMR, so misclassification was not likely. | Y / PY / **PN** / N / NI |
|  | **Risk of bias judgement** | **Moderate risk of bias:** i) Intervention status is well defined AND ii) Some aspects of the assignments of intervention status were done retrospectively. | Low / **Moderate** / Serious / Critical / NI |
|  | Optional: What is the predicted direction of bias due to classification of interventions? | Unpredictable | Favours experimental / Favours comparator / Towards null /Away from null / **Unpredictable** |

| **Bias due to deviations from intended interventions** | | | |
| --- | --- | --- | --- |
|  | **If your aim for this study is to assess the effect of assignment to intervention, answer questions 4.1 and 4.2** | |  |
|  | 4.1. Were there deviations from the intended intervention beyond what would be expected in usual practice? | No. There were no deviations from the intended intervention. | Y / PY / PN / **N** / NI |
|  | 4.2. **If Y/PY to 4.1**: Were these deviations from intended intervention unbalanced between groups *and* likely to have affected the outcome? |  | NA / Y / PY / PN / N / NI |
|  | **If your aim for this study is to assess the effect of starting and adhering to intervention, answer questions 4.3 to 4.6** | |  |
|  | 4.3. Were important co-interventions balanced across intervention groups? | Yes. Important co-interventions were balanced across the intervention and control group. | **Y** / PY / PN / N / NI |
|  | 4.4. Was the intervention implemented successfully for most participants? | Yes. The intervention was implemented successfully for all participants. | **Y** / PY / PN / N / NI |
|  | 4.5. Did study participants adhere to the assigned intervention regimen? | Yes. Study participants adhered to the assigned intervention regimen. | **Y** / PY / PN / N / NI |
|  | 4.6. **If N/PN to 4.3, 4.4 or 4.5**: Was an appropriate analysis used to estimate the effect of starting and adhering to the intervention? |  | NA / Y / PY / PN / N / NI |
|  | **Risk of bias judgement** | **Low risk of bias:** i) Any deviation from the intended intervention reflected usual practice. | **Low** / Moderate / Serious / Critical / NI |
|  | Optional: What is the predicted direction of bias due to deviations from the intended interventions? | Unpredictable | Favours experimental / Favours comparator / Towards null /Away from null / **Unpredictable** |

| **Bias due to missing data** | | | |
| --- | --- | --- | --- |
|  | 5.1 Were outcome data available for all, or nearly all, participants? | Yes. Outcome data was available for all participants. | **Y** / PY / PN / N / NI |
|  | 5.2 Were participants excluded due to missing data on intervention status? | No. Participants were not excluded since there was no missing data on the intervention status. | Y / PY / PN / **N** / NI |
|  | 5.3 Were participants excluded due to missing data on other variables needed for the analysis? | No. Participants were not excluded since there was no missing data on the other variables needed for the analysis. | Y / PY / PN / **N** / NI |
|  | 5.4 **If PN/N to 5.1, or Y/PY to 5.2 or 5.3**: Are the proportion of participants and reasons for missing data similar across interventions? |  | NA / Y / PY / PN / N / NI |
|  | 5.5 **If PN/N to 5.1, or Y/PY to 5.2 or 5.3**: Is there evidence that results were robust to the presence of missing data? |  | NA / Y / PY / PN / N / NI |
|  | **Risk of bias judgement** | **Low risk of bias:** i) Data were reasonably complete. | **Low** / Moderate / Serious / Critical / NI |
|  | Optional: What is the predicted direction of bias due to missing data? | Unpredictable | Favours experimental / Favours comparator / Towards null /Away from null / **Unpredictable** |

| **Bias in measurement of outcomes** | | | |
| --- | --- | --- | --- |
|  | 6.1 Could the outcome measure have been influenced by knowledge of the intervention received? | No. The primary outcome was intubation or death, which is a hard outcome; so, no influence by knowledge of the intervention received could have been done. | Y / PY / PN / **N** / NI |
|  | 6.2 Were outcome assessors aware of the intervention received by study participants? | Yes. Since it is a retrospective cohort study, the outcome assessors were fully aware of the intervention received by study participants. | **Y** / PY / PN / N / NI |
|  | 6.3 Were the methods of outcome assessment comparable across intervention groups? | Yes. The primary outcome was intubation or death, which is a hard outcome; so, methods of outcome assessment were the same for both. | **Y** / PY / PN / N / NI |
|  | 6.4 Were any systematic errors in measurement of the outcome related to intervention received? | No. There were no systematic errors in measurement of the outcome, since it was a hard outcome. | Y / PY / PN / **N** / NI |
|  | **Risk of bias judgement** | **Low risk of bias:** i) The methods of outcome assessment were comparable across intervention groups AND ii) The outcome measure was unlikely to be influenced by knowledge of the intervention received by study participants AND iii) Any error in measuring the outcome is unrelated to intervention status. | **Low** / Moderate / Serious / Critical / NI |
|  | Optional: What is the predicted direction of bias due to measurement of outcomes? | Unpredictable | Favours experimental / Favours comparator / Towards null /Away from null / **Unpredictable** |

| **Bias in selection of the reported result** | | | |
| --- | --- | --- | --- |
|  | Is the reported effect estimate likely to be selected, on the basis of the results, from... |  |  |
|  | 7.1. ... multiple outcome *measurements* within the outcome domain? | No. The reported effect estimate was not likely to be selected from multiple outcome measurements. | Y / PY / PN / **N** / NI |
|  | 7.2 ... multiple *analyses* of the intervention-outcome relationship? | No. The reported effect estimate was not likely to be selected from multiple analysed of the intervention-outcome relationship. Although, the authors did an unadjusted multivariate analysis and two propensity-score analysis (one including inverse probability weighting); they presented all their complete analyses. | Y / PY / PN / **N** / NI |
|  | 7.3 ... different *subgroups*? | No. The reported effect estimate was not likely to be selected from different subgroups. | Y / PY / PN / **N** / NI |
|  | **Risk of bias judgement** | **Low risk of bias:** There is clear evidence that all reported results correspond to all intended outcome, analysis, and sub-cohorts. | **Low** / Moderate / Serious / Critical / NI |
|  | Optional: What is the predicted direction of bias due to selection of the reported result? | Unpredictable | Favours experimental / Favours comparator / Towards null /Away from null / **Unpredictable** |

| **Overall bias** | | | |
| --- | --- | --- | --- |
|  | **Risk of bias judgement** | **Serious risk of bias:** This study was judged to have serious risk of bias in one out of seven domains. | Low / Moderate / **Serious** / Critical / NI |
|  | Optional: What is the overall predicted direction of bias for this outcome? | Unpredictable | Favours experimental / Favours comparator / Towards null /Away from null / **Unpredictable** |


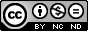


This work is licensed under a [Creative Commons Attribution-NonCommercial-NoDerivatives 4.0 International License](http://creativecommons.org/licenses/by-nc-nd/4.0/).

# ROBINS-I tool (Stage II): For each study

**Rossi, B.; Nguyen, L.S.; Zimmermann, P.; Boucenna, F.; Dubret, L.; Baucher, L.; Guillot, H.; Bouldouyre, M.-A.; Allenbach, Y.; Salem, J.-E.; Barsoum, P.; Oufella, A.; Gros, H. Effect of Tocilizumab in Hospitalized Patients with Severe COVID-19 Pneumonia: A Case-Control Cohort Study. Pharmaceuticals 2020, 13, 317.**

## Specify a target randomized trial specific to the study

| Design | Individually randomized / Cluster randomized / **Matched (e.g. cross-over)** |
| --- | --- |
| Participants | Adult hospitalized COVID-19 patients (SpO2≤96% with O2≥ 6L/min) without MV |
| Experimental intervention | Tocilizumab 400mg IV, single dose + SOC |
| Comparator | Standard of care (antibiotics, antivirals, steroids, baricitinib) |

## Is your aim for this study…?

| **X** | **to assess the effect of *assignment to* intervention** |
| --- | --- |
| □ | to assess the effect of *starting and adhering to* intervention |

## Specify the outcome

Specify which outcome is being assessed for risk of bias (typically from among those earmarked for the Summary of Findings table). Specify whether this is a proposed benefit or harm of intervention.

| Composite of mortality and ventilation at day 28 |
| --- |

## Specify the numerical result being assessed

In case of multiple alternative analyses being presented, specify the numeric result (e.g. RR = 1.52 (95% CI 0.83 to 2.77) and/or a reference (e.g. to a table, figure or paragraph) that uniquely defines the result being assessed.

| Propensity-score matched: HR0.49 (95%CI 0.3-0.81); Cox multivariable analysis: HR 0.26 (95%CI 0.125-0.51) |
| --- |

## Preliminary consideration of confounders

Complete a row for each important confounding domain (i) listed in the review protocol; and (ii) relevant to the setting of this particular study, or which the study authors identified as potentially important.

#### “Important” confounding domains are those for which, in the context of this study, adjustment is expected to lead to a clinically important change in the estimated effect of the intervention. “Validity” refers to whether the confounding variable or variables fully measure the domain, while “reliability” refers to the precision of the measurement (more measurement error means less reliability).

| **(i) Confounding domains listed in the review protocol** | | | | |
| --- | --- | --- | --- | --- |
| Confounding domain | Measured variable(s) | Is there evidence that controlling for this variable was unnecessary?* | Is the confounding domain measured validly and reliably by this variable (or these variables)? | OPTIONAL: Is failure to adjust for this variable (alone) expected to favour the experimental intervention or the comparator? |
| There are no confounding domains described in the protocol. |  |  |  |  |

| **(ii) Additional confounding domains relevant to the setting of this particular study, or which the study authors identified as important** | | | | |
| --- | --- | --- | --- | --- |
| Confounding domain | Measured variable(s) | Is there evidence that controlling for this variable was unnecessary?* | Is the confounding domain measured validly and reliably by this variable (or these variables)? | OPTIONAL: Is failure to adjust for this variable (alone) expected to favour the experimental intervention or the comparator? |
| Demographics | Age, male gender, BMI, obesity hypertension, diabetes, CV disease, pulmonary disease, smoking status | There is no evidence controlling was unnecessary | **Yes** / No / No information | Favour experimental / Favour comparator / **No information** |
| Baseline inflammatory markers | CRP, D-Dimer, IL-6, ferritin, LDH, lymphocyte count | There is no evidence controlling was unnecessary | **Yes** / No / No information | Favour experimental / Favour comparator / **No information** |
| Baseline severity of disease | SpO2, PaFiO2, O2 flow support, PaO2, PaCO2, | There is no evidence controlling was unnecessary | **Yes** / No / No information | Favour experimental / Favour comparator / **No information** |

* In the context of a particular study, variables can be demonstrated not to be confounders and so not included in the analysis: (a) if they are not predictive of the outcome; (b) if they are not predictive of intervention; or (c) because adjustment makes no or minimal difference to the estimated effect of the primary parameter. Note that “no statistically significant association” is not the same as “not predictive”.

## Preliminary consideration of co-interventions

Complete a row for each important co-intervention (i) listed in the review protocol; and (ii) relevant to the setting of this particular study, or which the study authors identified as important.

#### “Important” co-interventions are those for which, in the context of this study, adjustment is expected to lead to a clinically important change in the estimated effect of the intervention.

| **(i) Co-interventions listed in the review protocol** | | |
| --- | --- | --- |
| Co-intervention | Is there evidence that controlling for this co-intervention was unnecessary (e.g. because it was not administered)? | Is presence of this co-intervention likely to favour outcomes in the experimental intervention or the comparator |
| There are no co-interventions described in the protocol. |  |  |

| **(ii) Additional co-interventions relevant to the setting of this particular study, or which the study authors identified as important** | | |
| --- | --- | --- |
| Co-intervention | Is there evidence that controlling for this co-intervention was unnecessary (e.g. because it was not administered)? | Is presence of this co-intervention likely to favour outcomes in the experimental intervention or the comparator |
| Antibiotics | There is no evidence controlling for co-intervention was unnecessary. | Favour experimental / Favour comparator / **No information** |
| Antivirals | There is no evidence controlling for co-intervention was unnecessary. | Favour experimental / Favour comparator / **No information** |
| Hydroxychloroquine | There is no evidence controlling for co-intervention was unnecessary. | Favour experimental / Favour comparator / **No information** |
| Lopinavir / Ritonavir | There is no evidence controlling for co-intervention was unnecessary. | Favour experimental / Favour comparator / **No information** |
| Immunosuppressants or steroids | There is no evidence controlling for co-intervention was unnecessary. | Favour experimental / Favour comparator / **No information** |
| Baricitinib | There is no evidence controlling for co-intervention was unnecessary. | Favour experimental / Favour comparator / **No information** |

## Risk of bias assessment

Responses underlined in green are potential markers for low risk of bias, and responses in red are potential markers for a risk of bias. Where questions relate only to sign posts to other questions, no formatting is used.

|  | **Signalling questions** | **Description** | **Response options** |
| --- | --- | --- | --- |
| **Bias due to confounding** | | | |
|  | 1.1 Is there potential for confounding of the effect of intervention in this study?  **If N/PN to 1.1:** the study can be considered to be at low risk of bias due to confounding and no further signalling questions need be considered | Yes. There are potential confounders of the effect of intervention. | **Y** / PY / PN / N |
|  | **If Y/PY to 1.1**: determine whether there is a need to assess time-varying confounding: |  |  |
|  | 1.2. Was the analysis based on splitting participants’ follow up time according to intervention received?  **If N/PN**, answer questions relating to baseline confounding (1.4 to 1.6)  **If Y/PY**, go to question 1.3. | No. The intervention received did not change over time and post-baseline prognostic factors could not have affected the effect of intervention. | NA / Y / PY / PN / **N** / NI |
|  | 1.3. Were intervention discontinuations or switches likely to be related to factors that are prognostic for the outcome?  **If N/PN**, answer questions relating to baseline confounding (1.4 to 1.6)  **If Y/PY**, answer questions relating to both baseline and time-varying confounding (1.7 and 1.8) |  | NA / Y / PY / PN / N / NI |

|  | **Questions relating to baseline confounding only** | | |
| --- | --- | --- | --- |
|  | 1.4. Did the authors use an appropriate analysis method that controlled for all the important confounding domains? | Yes. The authors used inverse probability weighting and a Cox multivariable analysis to control the confounding domains. | NA / **Y** / PY / PN / N / NI |
|  | 1.5. **If Y/PY to 1.4**: Were confounding domains that were controlled for measured validly and reliably by the variables available in this study? | Yes. The authors measured demographic factors, baseline clinical factors, laboratory tests, and co-interventions validly and reliably to control confounding domains. | NA / **Y** / PY / PN / N / NI |
|  | 1.6. Did the authors control for any post-intervention variables that could have been affected by the intervention? | No. The authors did not control any post-baseline variables that could have affected the effect of intervention. | NA / Y / PY / PN / **N** / NI |
|  | **Questions relating to baseline and time-varying confounding** | |  |
|  | 1.7. Did the authors use an appropriate analysis method that controlled for all the important confounding domains and for time-varying confounding? | Yes. The authors used inverse probability weighting and a Cox multivariable analysis to control the confounding domains and time-varying confounding. | NA / **Y** / PY / PN / N / NI |
|  | 1.8. **If Y/PY to 1.7**: Were confounding domains that were controlled for measured validly and reliably by the variables available in this study? | Yes. The authors measured demographic factors, baseline clinical factors, laboratory tests, and co-interventions validly and reliably to control confounding domains. | NA / **Y** / PY / PN / N / NI |
|  | **Risk of bias judgement** | **Moderate risk of bias:** i) Confounding expected, all known important confounding domains appropriately measured and controlled for AND ii) Reliability and validity of measurement of important domains were sufficient, such that we do not expect serious residual confounding. | Low / **Moderate** / Serious / Critical / NI |
|  | Optional: What is the predicted direction of bias due to confounding? | Unpredictable | Favours experimental / Favours comparator / **Unpredictable** |

| **Bias in selection of participants into the study** | | | |
| --- | --- | --- | --- |
|  | 2.1. Was selection of participants into the study (or into the analysis) based on participant characteristics observed after the start of intervention?  **If N/PN to 2.1:** go to 2.4 | No. The selection of participants into the study was not based on participants characteristics observed after the start of the intervention. | Y / PY / PN / **N** / NI |
|  | 2.2. **If Y/PY to 2.1**: Were the post-intervention variables that influenced selection likely to be associated with intervention?  2.3 **If Y/PY to 2.2**: Were the post-intervention variables that influenced selection likely to be influenced by the outcome or a cause of the outcome? |  | NA / Y / PY / PN / N / NI  NA / Y / PY / PN / N / NI |
|  | 2.4. Do start of follow-up and start of intervention coincide for most participants? | No. There are specific variables on Table 1 measuring the delay between the first symptoms and hospital admission, and study inclusion. | Y / PY / PN / **N** / NI |
|  | 2.5. **If Y/PY to 2.2 and 2.3, or N/PN to 2.4**: Were adjustment techniques used that are likely to correct for the presence of selection biases? | Yes. The authors used inverse probability weighting to correct for the presence of selection biases. | NA / **Y** / PY / PN / N / NI |
|  | **Risk of bias judgement** | **Moderate risk of bias:** ii) Start of the follow-up and start of the intervention do not coincide for all participants AND a) The authors used appropriate methods to adjust for the selection bias. | Low / **Moderate** / Serious / Critical / NI |
|  | Optional: What is the predicted direction of bias due to selection of participants into the study? | Unpredictable | Favours experimental / Favours comparator / Towards null /Away from null / **Unpredictable** |

| **Bias in classification of interventions** | | | |
| --- | --- | --- | --- |
|  | 3.1 Were intervention groups clearly defined? | Yes. The intervention and control groups were clearly defined. | **Y** / PY / PN / N / NI |
|  | 3.2 Was the information used to define intervention groups recorded at the start of the intervention? | Probably yes. The study was conducted on France, so they probably used Electronic Medical Records (EMR) to define the start of the intervention. | Y / **PY** / PN / N / NI |
|  | 3.3 Could classification of intervention status have been affected by knowledge of the outcome or risk of the outcome? | Probably no. Classification of the intervention status was done retrospectively according to EMR, so misclassification was not likely. | Y / PY / **PN** / N / NI |
|  | **Risk of bias judgement** | **Moderate risk of bias:** i) Intervention status is well defined AND ii) Some aspects of the assignments of intervention status were done retrospectively. | Low / **Moderate** / Serious / Critical / NI |
|  | Optional: What is the predicted direction of bias due to classification of interventions? | Unpredictable | Favours experimental / Favours comparator / Towards null /Away from null / **Unpredictable** |

| **Bias due to deviations from intended interventions** | | | |
| --- | --- | --- | --- |
|  | **If your aim for this study is to assess the effect of assignment to intervention, answer questions 4.1 and 4.2** | |  |
|  | 4.1. Were there deviations from the intended intervention beyond what would be expected in usual practice? | No. There were no deviations from the intended intervention. | Y / PY / PN / **N** / NI |
|  | 4.2. **If Y/PY to 4.1**: Were these deviations from intended intervention unbalanced between groups *and* likely to have affected the outcome? |  | NA / Y / PY / PN / N / NI |
|  | **If your aim for this study is to assess the effect of starting and adhering to intervention, answer questions 4.3 to 4.6** | |  |
|  | 4.3. Were important co-interventions balanced across intervention groups? | No. There are some disbalances across the intervention groups, specifically in macrolides, hydroxychloroquine, and lopinavir/ritonavir. | Y / PY / PN / **N** / NI |
|  | 4.4. Was the intervention implemented successfully for most participants? | Yes. The intervention was implemented successfully for all participants. | **Y** / PY / PN / N / NI |
|  | 4.5. Did study participants adhere to the assigned intervention regimen? | Yes. Study participants adhered to the assigned intervention regimen. | **Y** / PY / PN / N / NI |
|  | 4.6. **If N/PN to 4.3, 4.4 or 4.5**: Was an appropriate analysis used to estimate the effect of starting and adhering to the intervention? | Yes. The authors used IPTW analysis to estimate the effect of starting and adhering to the intervention. | NA / **Y** / PY / PN / N / NI |
|  | **Risk of bias judgement** | **Moderate risk of bias:** ii) The important co-interventions were not balanced across the intervention groups. | Low / **Moderate** / Serious / Critical / NI |
|  | Optional: What is the predicted direction of bias due to deviations from the intended interventions? | Unpredictable | Favours experimental / Favours comparator / Towards null /Away from null / **Unpredictable** |

| **Bias due to missing data** | | | |
| --- | --- | --- | --- |
|  | 5.1 Were outcome data available for all, or nearly all, participants? | Yes. Outcome data was available for nearly all participants. On Figure 2, they show outcome data on 138 out of 140 patients in the intervention group. | **Y** / PY / PN / N / NI |
|  | 5.2 Were participants excluded due to missing data on intervention status? | No. Participants were not excluded due to missing data. | Y / PY / PN / **N** / NI |
|  | 5.3 Were participants excluded due to missing data on other variables needed for the analysis? | No. Participants were not excluded since there was no missing data on the other variables needed for the analysis. | Y / PY / PN / **N** / NI |
|  | 5.4 **If PN/N to 5.1, or Y/PY to 5.2 or 5.3**: Are the proportion of participants and reasons for missing data similar across interventions? |  | NA / Y / PY / PN / N / NI |
|  | 5.5 **If PN/N to 5.1, or Y/PY to 5.2 or 5.3**: Is there evidence that results were robust to the presence of missing data? |  | NA / Y / PY / PN / N / NI |
|  | **Risk of bias judgement** | **Low risk of bias:** i) Data were reasonably complete. | **Low** / Moderate / Serious / Critical / NI |
|  | Optional: What is the predicted direction of bias due to missing data? | Unpredictable | Favours experimental / Favours comparator / Towards null /Away from null / **Unpredictable** |

| **Bias in measurement of outcomes** | | | |
| --- | --- | --- | --- |
|  | 6.1 Could the outcome measure have been influenced by knowledge of the intervention received? | No. The primary outcome was a composite of death or ventilation at day 28, which is a hard outcome; so, no influence by knowledge of the intervention received could have been done. | Y / PY / PN / **N** / NI |
|  | 6.2 Were outcome assessors aware of the intervention received by study participants? | Yes. Since it is a retrospective cohort study, the outcome assessors were fully aware of the intervention received by study participants. | **Y** / PY / PN / N / NI |
|  | 6.3 Were the methods of outcome assessment comparable across intervention groups? | Yes. The primary outcome was a composite of death or ventilation, which is a hard outcome; so, methods of outcome assessment were the same for both. | **Y** / PY / PN / N / NI |
|  | 6.4 Were any systematic errors in measurement of the outcome related to intervention received? | No. There were no systematic errors in measurement of the outcome, since it was a hard outcome. | Y / PY / PN / **N** / NI |
|  | **Risk of bias judgement** | **Low risk of bias:** i) The methods of outcome assessment were comparable across intervention groups AND ii) The outcome measure was unlikely to be influenced by knowledge of the intervention received by study participants AND iii) Any error in measuring the outcome is unrelated to intervention status. | **Low** / Moderate / Serious / Critical / NI |
|  | Optional: What is the predicted direction of bias due to measurement of outcomes? | Unpredictable | Favours experimental / Favours comparator / Towards null /Away from null / **Unpredictable** |

| **Bias in selection of the reported result** | | | |
| --- | --- | --- | --- |
|  | Is the reported effect estimate likely to be selected, on the basis of the results, from... |  |  |
|  | 7.1. ... multiple outcome *measurements* within the outcome domain? | No. The reported effect estimate was not likely to be selected from multiple outcome measurements. | Y / PY / PN / **N** / NI |
|  | 7.2 ... multiple *analyses* of the intervention-outcome relationship? | No. The reported effect estimate was not likely to be selected from multiple analysed of the intervention-outcome relationship. Although, the authors did an Cox multivariable analysis and a propensity-score analysis (including inverse probability weighting); they presented all their complete analyses. | Y / PY / PN / **N** / NI |
|  | 7.3 ... different *subgroups*? | No. The reported effect estimate was not likely to be selected from different subgroups. | Y / PY / PN / **N** / NI |
|  | **Risk of bias judgement** | **Low risk of bias:** There is clear evidence that all reported results correspond to all intended outcome, analysis, and sub-cohorts. | **Low** / Moderate / Serious / Critical / NI |
|  | Optional: What is the predicted direction of bias due to selection of the reported result? | Unpredictable | Favours experimental / Favours comparator / Towards null /Away from null / **Unpredictable** |

| **Overall bias** | | | |
| --- | --- | --- | --- |
|  | **Risk of bias judgement** | **Moderate risk of bias:** This study is judged to have moderate risk of bias in four out of seven domains. | Low / **Moderate** / Serious / Critical / NI |
|  | Optional: What is the overall predicted direction of bias for this outcome? | Unpredictable | Favours experimental / Favours comparator / Towards null /Away from null / Unpredictable |


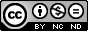


This work is licensed under a [Creative Commons Attribution-NonCommercial-NoDerivatives 4.0 International License](http://creativecommons.org/licenses/by-nc-nd/4.0/).

# ROBINS-I tool (Stage II): For each study

﻿**Hill JA, Menon MP, Dhanireddy S, et al. Tocilizumab in hospitalized patients with COVID‐19: Clinical outcomes, inflammatory marker kinetics, and safety. J Med Virol. 2020;1–11. https://doi.org/10.1002/jmv.26674**

## Specify a target randomized trial specific to the study

| Design | Individually randomized / Cluster randomized / **Matched (e.g. cross-over)** |
| --- | --- |
| Participants | Hospitalized, adult, PCR-confirmed SARS-CoV-2 patients with supplemental oxygen |
| Experimental intervention | Tocilizumab 400mg IV, single dose + Standard of care |
| Comparator | Standard of care |

## Is your aim for this study…?

| **X** | **to assess the effect of *assignment to* intervention** |
| --- | --- |
| □ | to assess the effect of *starting and adhering to* intervention |

## Specify the outcome

Specify which outcome is being assessed for risk of bias (typically from among those earmarked for the Summary of Findings table). Specify whether this is a proposed benefit or harm of intervention.

| Sustained (≥3 days) clinical improvement within 28 days |
| --- |

## Specify the numerical result being assessed

In case of multiple alternative analyses being presented, specify the numeric result (e.g. RR = 1.52 (95% CI 0.83 to 2.77) and/or a reference (e.g. to a table, figure or paragraph) that uniquely defines the result being assessed.

| HR 0.92 (95%CI 0.38-2.22) |
| --- |

## Preliminary consideration of confounders

Complete a row for each important confounding domain (i) listed in the review protocol; and (ii) relevant to the setting of this particular study, or which the study authors identified as potentially important.

#### “Important” confounding domains are those for which, in the context of this study, adjustment is expected to lead to a clinically important change in the estimated effect of the intervention. “Validity” refers to whether the confounding variable or variables fully measure the domain, while “reliability” refers to the precision of the measurement (more measurement error means less reliability).

| **(i) Confounding domains listed in the review protocol** | | | | |
| --- | --- | --- | --- | --- |
| Confounding domain | Measured variable(s) | Is there evidence that controlling for this variable was unnecessary?* | Is the confounding domain measured validly and reliably by this variable (or these variables)? | OPTIONAL: Is failure to adjust for this variable (alone) expected to favour the experimental intervention or the comparator? |
| There are no confounding domains described in the protocol. |  |  |  |  |

| **(ii) Additional confounding domains relevant to the setting of this particular study, or which the study authors identified as important** | | | | |
| --- | --- | --- | --- | --- |
| Confounding domain | Measured variable(s) | Is there evidence that controlling for this variable was unnecessary?* | Is the confounding domain measured validly and reliably by this variable (or these variables)? | OPTIONAL: Is failure to adjust for this variable (alone) expected to favour the experimental intervention or the comparator? |
| Demographics | Age, race and ethnicity, BMI, comorbidities | There is no evidence controlling was unnecessary | **Yes** / No / No information | Favour experimental / Favour comparator / **No information** |
| Baseline inflammatory markers | CRP, D-dimer, IL-6, ferritin, LDH, fibrinogen | There is no evidence controlling was unnecessary | **Yes** / No / No information | Favour experimental / Favour comparator / **No information** |
| Baseline severity of disease | Oxygen support categories | There is no evidence controlling was unnecessary | **Yes** / No / No information | Favour experimental / Favour comparator / **No information** |

* In the context of a particular study, variables can be demonstrated not to be confounders and so not included in the analysis: (a) if they are not predictive of the outcome; (b) if they are not predictive of intervention; or (c) because adjustment makes no or minimal difference to the estimated effect of the primary parameter. Note that “no statistically significant association” is not the same as “not predictive”.

## Preliminary consideration of co-interventions

Complete a row for each important co-intervention (i) listed in the review protocol; and (ii) relevant to the setting of this particular study, or which the study authors identified as important.

#### “Important” co-interventions are those for which, in the context of this study, adjustment is expected to lead to a clinically important change in the estimated effect of the intervention.

| **(i) Co-interventions listed in the review protocol** | | |
| --- | --- | --- |
| Co-intervention | Is there evidence that controlling for this co-intervention was unnecessary (e.g. because it was not administered)? | Is presence of this co-intervention likely to favour outcomes in the experimental intervention or the comparator |
| There are no co-interventions described in the protocol. |  |  |

| **(ii) Additional co-interventions relevant to the setting of this particular study, or which the study authors identified as important** | | |
| --- | --- | --- |
| Co-intervention | Is there evidence that controlling for this co-intervention was unnecessary (e.g. because it was not administered)? | Is presence of this co-intervention likely to favour outcomes in the experimental intervention or the comparator |
| Hydroxychloroquine, open-label | There is no evidence controlling for co-intervention was unnecessary. | Favour experimental / Favour comparator / **No information** |
| Hydroxychloroquine or placebo, blinded trial | There is no evidence controlling for co-intervention was unnecessary. | Favour experimental / Favour comparator / **No information** |
| Remdesivir, open-label | There is no evidence controlling for co-intervention was unnecessary. | Favour experimental / Favour comparator / **No information** |
| Remdesivir or placebo, blinded trial | There is no evidence controlling for co-intervention was unnecessary. | Favour experimental / Favour comparator / **No information** |

## Risk of bias assessment

Responses underlined in green are potential markers for low risk of bias, and responses in red are potential markers for a risk of bias. Where questions relate only to sign posts to other questions, no formatting is used.

|  | **Signalling questions** | **Description** | **Response options** |
| --- | --- | --- | --- |
| **Bias due to confounding** | | | |
|  | 1.1 Is there potential for confounding of the effect of intervention in this study?  **If N/PN to 1.1:** the study can be considered to be at low risk of bias due to confounding and no further signalling questions need be considered | Yes. There are potential confounders of the effect of intervention. | **Y** / PY / PN / N |
|  | **If Y/PY to 1.1**: determine whether there is a need to assess time-varying confounding: |  |  |
|  | 1.2. Was the analysis based on splitting participants’ follow up time according to intervention received?  **If N/PN**, answer questions relating to baseline confounding (1.4 to 1.6)  **If Y/PY**, go to question 1.3. | No. The intervention received did not change over time and post-baseline prognostic factors could not have affected the effect of intervention. | NA / Y / PY / PN / **N** / NI |
|  | 1.3. Were intervention discontinuations or switches likely to be related to factors that are prognostic for the outcome?  **If N/PN**, answer questions relating to baseline confounding (1.4 to 1.6)  **If Y/PY**, answer questions relating to both baseline and time-varying confounding (1.7 and 1.8) |  | NA / Y / PY / PN / N / NI |

|  | **Questions relating to baseline confounding only** | | |
| --- | --- | --- | --- |
|  | 1.4. Did the authors use an appropriate analysis method that controlled for all the important confounding domains? | Yes. The authors used inverse probability weighting and an adjusted Cox model to control the confounding domains. | NA / **Y** / PY / PN / N / NI |
|  | 1.5. **If Y/PY to 1.4**: Were confounding domains that were controlled for measured validly and reliably by the variables available in this study? | Yes. The authors measured demographic factors, baseline clinical factors, laboratory tests, and co-interventions validly and reliably to control confounding domains. | NA / **Y** / PY / PN / N / NI |
|  | 1.6. Did the authors control for any post-intervention variables that could have been affected by the intervention? | No. The authors did not control any post-baseline variables that could have affected the effect of intervention. | NA / Y / PY / PN / **N** / NI |
|  | **Questions relating to baseline and time-varying confounding** | | **Questions relating to baseline and time-varying confounding** |
|  | 1.7. Did the authors use an appropriate analysis method that controlled for all the important confounding domains and for time-varying confounding? | Yes. The authors used inverse probability weighting and an adjusted Cox model to control the confounding domains and time-varying confounding. | NA / **Y** / PY / PN / N / NI |
|  | 1.8. **If Y/PY to 1.7**: Were confounding domains that were controlled for measured validly and reliably by the variables available in this study? | Yes. The authors measured demographic factors, baseline clinical factors, laboratory tests, and co-interventions validly and reliably to control confounding domains. | NA / **Y** / PY / PN / N / NI |
|  | **Risk of bias judgement** | **Moderate risk of bias:** i) Confounding expected, all known important confounding domains appropriately measured and controlled for AND ii) Reliability and validity of measurement of important domains were sufficient, such that we do not expect serious residual confounding. | Low / **Moderate** / Serious / Critical / NI |
|  | Optional: What is the predicted direction of bias due to confounding? | Unpredictable | Favours experimental / Favours comparator / **Unpredictable** |

| **Bias in selection of participants into the study** | | | |
| --- | --- | --- | --- |
|  | 2.1. Was selection of participants into the study (or into the analysis) based on participant characteristics observed after the start of intervention?  **If N/PN to 2.1:** go to 2.4 | No. The selection of participants into the study was not based on participants characteristics observed after the start of the intervention. | Y / PY / PN / **N** / NI |
|  | 2.2. **If Y/PY to 2.1**: Were the post-intervention variables that influenced selection likely to be associated with intervention?  2.3 **If Y/PY to 2.2**: Were the post-intervention variables that influenced selection likely to be influenced by the outcome or a cause of the outcome? |  | NA / Y / PY / PN / N / NI  NA / Y / PY / PN / N / NI |
|  | 2.4. Do start of follow-up and start of intervention coincide for most participants? | Yes. The authors considered baseline as the time of TCZ administration for the intervention group, and 2 days after hospitalization for the control group, to align with the median number of days between hospitalization and TCZ administration (1.8 days, 0.8-3.8) | **Y** / PY / PN / N / NI |
|  | 2.5. **If Y/PY to 2.2 and 2.3, or N/PN to 2.4**: Were adjustment techniques used that are likely to correct for the presence of selection biases? | Yes. The authors used inverse probability weighting to correct for the presence of selection biases. | NA / **Y** / PY / PN / N / NI |
|  | **Risk of bias judgement** | **Low risk of bias:** i) All participants who would have been eligible for the target trial were included in the study AND ii) For each participant, start of follow up and start of intervention coincided. | **Low** / Moderate / Serious / Critical / NI |
|  | Optional: What is the predicted direction of bias due to selection of participants into the study? | Unpredictable | Favours experimental / Favours comparator / Towards null /Away from null / **Unpredictable** |

| **Bias in classification of interventions** | | | |
| --- | --- | --- | --- |
|  | 3.1 Were intervention groups clearly defined? | Yes. The intervention and control groups were clearly defined. | **Y** / PY / PN / N / NI |
|  | 3.2 Was the information used to define intervention groups recorded at the start of the intervention? | Probably yes. The study was conducted on University of Washington Hospital system in Seattle, US. They used Electronic Medical Records (EMR) to define the start of the intervention. | **Y** / PY / PN / N / NI |
|  | 3.3 Could classification of intervention status have been affected by knowledge of the outcome or risk of the outcome? | Probably no. Classification of the intervention status was done retrospectively according to EMR, so misclassification was not likely. | Y / PY / **PN** / N / NI |
|  | **Risk of bias judgement** | **Moderate risk of bias:** i) Intervention status is well defined AND ii) Some aspects of the assignments of intervention status were done retrospectively. | Low / **Moderate** / Serious / Critical / NI |
|  | Optional: What is the predicted direction of bias due to classification of interventions? | Unpredictable | Favours experimental / Favours comparator / Towards null /Away from null / **Unpredictable** |

| **Bias due to deviations from intended interventions** | | | |
| --- | --- | --- | --- |
|  | **If your aim for this study is to assess the effect of assignment to intervention, answer questions 4.1 and 4.2** | |  |
|  | 4.1. Were there deviations from the intended intervention beyond what would be expected in usual practice? | No. There were no deviations from the intended intervention. | Y / PY / PN / **N** / NI |
|  | 4.2. **If Y/PY to 4.1**: Were these deviations from intended intervention unbalanced between groups *and* likely to have affected the outcome? |  | NA / Y / PY / PN / N / NI |
|  | **If your aim for this study is to assess the effect of starting and adhering to intervention, answer questions 4.3 to 4.6** | | **If your aim for this study is to assess the effect of starting and adhering to intervention, answer questions 4.3 to 4.6** |
|  | 4.3. Were important co-interventions balanced across intervention groups? | No. There are some disbalances across the intervention groups, specifically in hydroxychloroquine (open-label) | Y / PY / PN / **N** / NI |
|  | 4.4. Was the intervention implemented successfully for most participants? | Yes. The intervention was implemented successfully for all participants. | **Y** / PY / PN / N / NI |
|  | 4.5. Did study participants adhere to the assigned intervention regimen? | Yes. Study participants adhered to the assigned intervention regimen. | **Y** / PY / PN / N / NI |
|  | 4.6. **If N/PN to 4.3, 4.4 or 4.5**: Was an appropriate analysis used to estimate the effect of starting and adhering to the intervention? | Yes. The authors used IPTW analysis to estimate the effect of starting and adhering to the intervention. | NA / **Y** / PY / PN / N / NI |
|  | **Risk of bias judgement** | **Moderate risk of bias:** ii) The important co-interventions were not balanced across the intervention groups. | Low / **Moderate** / Serious / Critical / NI |
|  | Optional: What is the predicted direction of bias due to deviations from the intended interventions? | Unpredictable | Favours experimental / Favours comparator / Towards null /Away from null / **Unpredictable** |

| **Bias due to missing data** | | | |
| --- | --- | --- | --- |
|  | 5.1 Were outcome data available for all, or nearly all, participants? | Yes. Outcome data was available for all 88 participants. | **Y** / PY / PN / N / NI |
|  | 5.2 Were participants excluded due to missing data on intervention status? | No. Participants were not excluded due to missing data; since there were no missing data. | Y / PY / PN / **N** / NI |
|  | 5.3 Were participants excluded due to missing data on other variables needed for the analysis? | No. Participants were not excluded since there was no missing data on the other variables needed for the analysis. | Y / PY / PN / **N** / NI |
|  | 5.4 **If PN/N to 5.1, or Y/PY to 5.2 or 5.3**: Are the proportion of participants and reasons for missing data similar across interventions? |  | NA / Y / PY / PN / N / NI |
|  | 5.5 **If PN/N to 5.1, or Y/PY to 5.2 or 5.3**: Is there evidence that results were robust to the presence of missing data? |  | NA / Y / PY / PN / N / NI |
|  | **Risk of bias judgement** | **Low risk of bias:** i) Data were reasonably complete. | **Low** / Moderate / Serious / Critical / NI |
|  | Optional: What is the predicted direction of bias due to missing data? | Unpredictable | Favours experimental / Favours comparator / Towards null /Away from null / **Unpredictable** |

| **Bias in measurement of outcomes** | | | |
| --- | --- | --- | --- |
|  | 6.1 Could the outcome measure have been influenced by knowledge of the intervention received? | No. The primary outcome was a sustained (≥3 days) clinical improvement within 28 days, defined as an improvement of at least 2 points in a severity ordinal scale. This is a hard outcome; so, no influence by knowledge of the intervention received could have been done. | Y / PY / PN / **N** / NI |
|  | 6.2 Were outcome assessors aware of the intervention received by study participants? | Yes. This is a retrospective cohort study; so, the outcome assessors were fully aware of the intervention received by study participants. | **Y** / PY / PN / N / NI |
|  | 6.3 Were the methods of outcome assessment comparable across intervention groups? | Yes. The primary outcome was a sustained (≥3 days) clinical improvement within 28 days, defined as an improvement of at least 2 points in a severity ordinal scale. This is a hard outcome; so, methods of outcome assessment were the same for both. | **Y** / PY / PN / N / NI |
|  | 6.4 Were any systematic errors in measurement of the outcome related to intervention received? | No. There were no systematic errors in measurement of the outcome, since it was a hard outcome. | Y / PY / PN / **N** / NI |
|  | **Risk of bias judgement** | **Low risk of bias:** i) The methods of outcome assessment were comparable across intervention groups AND ii) The outcome measure was unlikely to be influenced by knowledge of the intervention received by study participants AND iii) Any error in measuring the outcome is unrelated to intervention status. | **Low** / Moderate / Serious / Critical / NI |
|  | Optional: What is the predicted direction of bias due to measurement of outcomes? | Unpredictable | Favours experimental / Favours comparator / Towards null /Away from null / **Unpredictable** |

| **Bias in selection of the reported result** | | | |
| --- | --- | --- | --- |
|  | Is the reported effect estimate likely to be selected, on the basis of the results, from... |  |  |
|  | 7.1. ... multiple outcome *measurements* within the outcome domain? | No. The reported effect estimate was not likely to be selected from multiple outcome measurements. | Y / PY / PN / **N** / NI |
|  | 7.2 ... multiple *analyses* of the intervention-outcome relationship? | No. The reported effect estimate was not likely to be selected from multiple analysed of the intervention-outcome relationship. The authors did an adjusted Cox model and presented their complete analyses. | Y / PY / PN / **N** / NI |
|  | 7.3 ... different *subgroups*? | No. The reported effect estimate was not likely to be selected from different subgroups. The authors did subgroups analysis of the primary outcome by oxygen support categories, but presented their complete analyses. | Y / PY / PN / **N** / NI |
|  | **Risk of bias judgement** | **Low risk of bias:** There is clear evidence that all reported results correspond to all intended outcome, analysis, and sub-cohorts. | **Low** / Moderate / Serious / Critical / NI |
|  | Optional: What is the predicted direction of bias due to selection of the reported result? | Unpredictable | Favours experimental / Favours comparator / Towards null /Away from null / **Unpredictable** |

| **Overall bias** | | | |
| --- | --- | --- | --- |
|  | **Risk of bias judgement** | **Moderate risk of bias:** This study was judged to have moderate risk of bias in two out of seven domains. | Low / **Moderate** / Serious / Critical / NI |
|  | Optional: What is the overall predicted direction of bias for this outcome? | Unpredictable | Favours experimental / Favours comparator / Towards null /Away from null / **Unpredictable** |


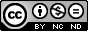


This work is licensed under a [Creative Commons Attribution-NonCommercial-NoDerivatives 4.0 International License](http://creativecommons.org/licenses/by-nc-nd/4.0/).

# ROBINS-I tool (Stage II): For each study

**Martinez-Sanz J, et al. ﻿ Effects of tocilizumab on mortality in hospitalized patients with COVID-19: a multicentre cohort study. ﻿Clinical Microbiology and Infection. 2020. DOI: https://doi.org/10.1016/j.cmi.2020.09.021**

## Specify a target randomized trial specific to the study

| Design | Individually randomized / Cluster randomized / **Matched (e.g. cross-over)** |
| --- | --- |
| Participants | Hospitalized, adult, PCR-confirmed SARS-CoV-2 infection |
| Experimental intervention | Tocilizumab |
| Comparator | Standard of care (HCQ, steroids, azithromycin, lopinavir/ritonavir) |

## Is your aim for this study…?

| **X** | **to assess the effect of *assignment to* intervention** |
| --- | --- |
| □ | to assess the effect of *starting and adhering to* intervention |

## Specify the outcome

Specify which outcome is being assessed for risk of bias (typically from among those earmarked for the Summary of Findings table). Specify whether this is a proposed benefit or harm of intervention.

| Time to in-hospital death for any cause |
| --- |

## Specify the numerical result being assessed

In case of multiple alternative analyses being presented, specify the numeric result (e.g. RR = 1.52 (95% CI 0.83 to 2.77) and/or a reference (e.g. to a table, figure or paragraph) that uniquely defines the result being assessed.

| CRP <150mg/dL: ﻿HR 1.21 (95%CI 0.65-2.23)  CRP >150mg/dL: ﻿HR 0.34 (95% CI 0.17-0.71) |
| --- |

## Preliminary consideration of confounders

Complete a row for each important confounding domain (i) listed in the review protocol; and (ii) relevant to the setting of this particular study, or which the study authors identified as potentially important.

#### “Important” confounding domains are those for which, in the context of this study, adjustment is expected to lead to a clinically important change in the estimated effect of the intervention. “Validity” refers to whether the confounding variable or variables fully measure the domain, while “reliability” refers to the precision of the measurement (more measurement error means less reliability).

| **(i) Confounding domains listed in the review protocol** | | | | |
| --- | --- | --- | --- | --- |
| Confounding domain | Measured variable(s) | Is there evidence that controlling for this variable was unnecessary?* | Is the confounding domain measured validly and reliably by this variable (or these variables)? | OPTIONAL: Is failure to adjust for this variable (alone) expected to favour the experimental intervention or the comparator? |
| There are no confounding domains described in the protocol. |  |  |  |  |

| **(ii) Additional confounding domains relevant to the setting of this particular study, or which the study authors identified as important** | | | | |
| --- | --- | --- | --- | --- |
| Confounding domain | Measured variable(s) | Is there evidence that controlling for this variable was unnecessary?* | Is the confounding domain measured validly and reliably by this variable (or these variables)? | OPTIONAL: Is failure to adjust for this variable (alone) expected to favour the experimental intervention or the comparator? |
| Demographics | Age, gender, comorbidities | There is no evidence controlling was unnecessary | **Yes** / No / No information | Favour experimental / Favour comparator / **No information** |
| Baseline inflammatory markers | Lymphocyte count, neutrophil count, D-dimer, IL-6, LDH, CRP, creatinine, urea | There is no evidence controlling was unnecessary | **Yes** / No / No information | Favour experimental / Favour comparator / **No information** |
| Baseline severity of disease | Vital signs at admission | There is no evidence controlling was unnecessary | **Yes** / No / No information | Favour experimental / Favour comparator / **No information** |

* In the context of a particular study, variables can be demonstrated not to be confounders and so not included in the analysis: (a) if they are not predictive of the outcome; (b) if they are not predictive of intervention; or (c) because adjustment makes no or minimal difference to the estimated effect of the primary parameter. Note that “no statistically significant association” is not the same as “not predictive”.

## Preliminary consideration of co-interventions

Complete a row for each important co-intervention (i) listed in the review protocol; and (ii) relevant to the setting of this particular study, or which the study authors identified as important.

#### “Important” co-interventions are those for which, in the context of this study, adjustment is expected to lead to a clinically important change in the estimated effect of the intervention.

| **(i) Co-interventions listed in the review protocol** | | |
| --- | --- | --- |
| Co-intervention | Is there evidence that controlling for this co-intervention was unnecessary (e.g. because it was not administered)? | Is presence of this co-intervention likely to favour outcomes in the experimental intervention or the comparator |
| There are no co-interventions described in the protocol. |  |  |

| **(ii) Additional co-interventions relevant to the setting of this particular study, or which the study authors identified as important** | | |
| --- | --- | --- |
| Co-intervention | Is there evidence that controlling for this co-intervention was unnecessary (e.g. because it was not administered)? | Is presence of this co-intervention likely to favour outcomes in the experimental intervention or the comparator |
| Steroids | There is no evidence controlling for co-intervention was unnecessary. | Favour experimental / Favour comparator / **No information** |
| Hydroxychloroquine | There is no evidence controlling for co-intervention was unnecessary. | Favour experimental / Favour comparator / **No information** |
| Azithromycin | There is no evidence controlling for co-intervention was unnecessary. | Favour experimental / Favour comparator / **No information** |
| Lopinavir/Ritonavir | There is no evidence controlling for co-intervention was unnecessary. | Favour experimental / Favour comparator / **No information** |

## Risk of bias assessment

Responses underlined in green are potential markers for low risk of bias, and responses in red are potential markers for a risk of bias. Where questions relate only to sign posts to other questions, no formatting is used.

|  | **Signalling questions** | **Description** | **Response options** |
| --- | --- | --- | --- |
| **Bias due to confounding** | | | |
|  | 1.1 Is there potential for confounding of the effect of intervention in this study?  **If N/PN to 1.1:** the study can be considered to be at low risk of bias due to confounding and no further signalling questions need be considered | Yes. There are potential confounders of the effect of intervention. | **Y** / PY / PN / N |
|  | **If Y/PY to 1.1**: determine whether there is a need to assess time-varying confounding: |  |  |
|  | 1.2. Was the analysis based on splitting participants’ follow up time according to intervention received?  **If N/PN**, answer questions relating to baseline confounding (1.4 to 1.6)  **If Y/PY**, go to question 1.3. | No. The intervention received did not change over time and post-baseline prognostic factors could not have affected the effect of intervention. | NA / Y / PY / PN / **N** / NI |
|  | 1.3. Were intervention discontinuations or switches likely to be related to factors that are prognostic for the outcome?  **If N/PN**, answer questions relating to baseline confounding (1.4 to 1.6)  **If Y/PY**, answer questions relating to both baseline and time-varying confounding (1.7 and 1.8) |  | NA / Y / PY / PN / N / NI |

|  | **Questions relating to baseline confounding only** | | |
| --- | --- | --- | --- |
|  | 1.4. Did the authors use an appropriate analysis method that controlled for all the important confounding domains? | Yes. The authors used inverse probability weighting and an adjusted Cox model to control the confounding domains. | NA / **Y** / PY / PN / N / NI |
|  | 1.5. **If Y/PY to 1.4**: Were confounding domains that were controlled for measured validly and reliably by the variables available in this study? | Yes. The authors measured demographic factors, vital signs at admission, baseline laboratory tests, and co-interventions validly and reliably to control confounding domains. | NA / **Y** / PY / PN / N / NI |
|  | 1.6. Did the authors control for any post-intervention variables that could have been affected by the intervention? | No. The authors did not control any post-baseline variables that could have affected the effect of intervention. | NA / Y / PY / PN / **N** / NI |
|  | **Questions relating to baseline and time-varying confounding** | | **Questions relating to baseline and time-varying confounding** |
|  | 1.7. Did the authors use an appropriate analysis method that controlled for all the important confounding domains and for time-varying confounding? | Yes. The authors used inverse probability weighting and an adjusted Cox model to control the confounding domains and time-varying confounding. | NA / **Y** / PY / PN / N / NI |
|  | 1.8. **If Y/PY to 1.7**: Were confounding domains that were controlled for measured validly and reliably by the variables available in this study? | Yes. The authors measured demographic factors, vital signs at admission, baseline laboratory tests, and co-interventions validly and reliably to control confounding domains. | NA / **Y** / PY / PN / N / NI |
|  | **Risk of bias judgement** | **Moderate risk of bias:** i) Confounding expected, all known important confounding domains appropriately measured and controlled for AND ii) Reliability and validity of measurement of important domains were sufficient, such that we do not expect serious residual confounding. | Low / **Moderate** / Serious / Critical / NI |
|  | Optional: What is the predicted direction of bias due to confounding? | Unpredictable | Favours experimental / Favours comparator / **Unpredictable** |

| **Bias in selection of participants into the study** | | | |
| --- | --- | --- | --- |
|  | 2.1. Was selection of participants into the study (or into the analysis) based on participant characteristics observed after the start of intervention?  **If N/PN to 2.1:** go to 2.4 | No. The selection of participants into the study was not based on participants characteristics observed after the start of the intervention. | Y / PY / PN / **N** / NI |
|  | 2.2. **If Y/PY to 2.1**: Were the post-intervention variables that influenced selection likely to be associated with intervention?  2.3 **If Y/PY to 2.2**: Were the post-intervention variables that influenced selection likely to be influenced by the outcome or a cause of the outcome? |  | NA / Y / PY / PN / N / NI  NA / Y / PY / PN / N / NI |
|  | 2.4. Do start of follow-up and start of intervention coincide for most participants? | No. The authors considered study baseline as the first day of hospitalization, but the median time to intervention was 4 (3-5) . | Y / PY / PN / **N** / NI |
|  | 2.5. **If Y/PY to 2.2 and 2.3, or N/PN to 2.4**: Were adjustment techniques used that are likely to correct for the presence of selection biases? | Yes. The authors used inverse probability weighting to correct for the presence of selection biases. | NA / **Y** / PY / PN / N / NI |
|  | **Risk of bias judgement** | **Moderate risk of bias:** ii) Start of the follow-up and start of the intervention do not coincide for all participants AND b) The authors used appropriate methods to adjust for the selection bias. | Low / **Moderate** / Serious / Critical / NI |
|  | Optional: What is the predicted direction of bias due to selection of participants into the study? | Unpredictable | Favours experimental / Favours comparator / Towards null /Away from null / **Unpredictable** |

| **Bias in classification of interventions** | | | |
| --- | --- | --- | --- |
|  | 3.1 Were intervention groups clearly defined? | Yes. The intervention and control groups were clearly defined. | **Y** / PY / PN / N / NI |
|  | 3.2 Was the information used to define intervention groups recorded at the start of the intervention? | Probably yes. The study was conducted at University Hospital Ramon y Cajal. They probably used Electronic Medical Records (EMR) to define the start of the intervention. | Y / **PY** / PN / N / NI |
|  | 3.3 Could classification of intervention status have been affected by knowledge of the outcome or risk of the outcome? | Probably no. Classification of the intervention status was done retrospectively probably according to EMR, so misclassification was not likely. | Y / PY / **PN** / N / NI |
|  | **Risk of bias judgement** | **Moderate risk of bias:** i) Intervention status is well defined AND ii) Some aspects of the assignments of intervention status were done retrospectively. | Low / **Moderate** / Serious / Critical / NI |
|  | Optional: What is the predicted direction of bias due to classification of interventions? | Unpredictable | Favours experimental / Favours comparator / Towards null /Away from null / **Unpredictable** |

| **Bias due to deviations from intended interventions** | | | |
| --- | --- | --- | --- |
|  | **If your aim for this study is to assess the effect of assignment to intervention, answer questions 4.1 and 4.2** | |  |
|  | 4.1. Were there deviations from the intended intervention beyond what would be expected in usual practice? | No. There were no deviations from the intended intervention. | Y / PY / PN / **N** / NI |
|  | 4.2. **If Y/PY to 4.1**: Were these deviations from intended intervention unbalanced between groups *and* likely to have affected the outcome? |  | NA / Y / PY / PN / N / NI |
|  | **If your aim for this study is to assess the effect of starting and adhering to intervention, answer questions 4.3 to 4.6** | | **If your aim for this study is to assess the effect of starting and adhering to intervention, answer questions 4.3 to 4.6** |
|  | 4.3. Were important co-interventions balanced across intervention groups? | No. There are some disbalances across the intervention groups, specifically in steroids and lopinavir/ritonavir | Y / PY / PN / **N** / NI |
|  | 4.4. Was the intervention implemented successfully for most participants? | Yes. The intervention was implemented successfully for all participants. | **Y** / PY / PN / N / NI |
|  | 4.5. Did study participants adhere to the assigned intervention regimen? | Yes. Study participants adhered to the assigned intervention regimen. | **Y** / PY / PN / N / NI |
|  | 4.6. **If N/PN to 4.3, 4.4 or 4.5**: Was an appropriate analysis used to estimate the effect of starting and adhering to the intervention? | Yes. The authors used IPTW analysis to estimate the effect of starting and adhering to the intervention. | NA / **Y** / PY / PN / N / NI |
|  | **Risk of bias judgement** | **Moderate risk of bias:** ii) The important co-interventions were not balanced across the intervention groups. | Low / **Moderate** / Serious / Critical / NI |
|  | Optional: What is the predicted direction of bias due to deviations from the intended interventions? | Unpredictable | Favours experimental / Favours comparator / Towards null /Away from null / **Unpredictable** |

| **Bias due to missing data** | | | |
| --- | --- | --- | --- |
|  | 5.1 Were outcome data available for all, or nearly all, participants? | Yes. Outcome data was available for nearly all participants | **Y** / PY / PN / N / NI |
|  | 5.2 Were participants excluded due to missing data on intervention status? | No. Participants were not excluded due to missing data on the intervention status. | Y / PY / PN / **N** / NI |
|  | 5.3 Were participants excluded due to missing data on other variables needed for the analysis? | Yes. Participants were excluded due to missing data on the other variables needed for the analysis. 64 and 655 participants were excluded, respectively. | **Y** / PY / PN / N / NI |
|  | 5.4 **If PN/N to 5.1, or Y/PY to 5.2 or 5.3**: Are the proportion of participants and reasons for missing data similar across interventions? | No. 64/324 (19.8) and 655/1624 (40) participants were excluded, respectively. The proportions were not similar across interventions. | NA / Y / PY / PN / **N** / NI |
|  | 5.5 **If PN/N to 5.1, or Y/PY to 5.2 or 5.3**: Is there evidence that results were robust to the presence of missing data? | No. The presence of missing data might have influenced the results of the outcomes. | NA / Y / PY / PN / **N** / NI |
|  | **Risk of bias judgement** | **Serious risk of bias:** i) Proportions of missing participants differ substantially across interventions AND ii) The analysis is unlikely to have removed the bias arising from the missing data. | Low / Moderate / **Serious** / Critical / NI |
|  | Optional: What is the predicted direction of bias due to missing data? | Unpredictable | Favours experimental / Favours comparator / Towards null /Away from null / **Unpredictable** |

| **Bias in measurement of outcomes** | | | |
| --- | --- | --- | --- |
|  | 6.1 Could the outcome measure have been influenced by knowledge of the intervention received? | No. The primary outcome was a time to in-hospital death for any cause, which is a hard outcome. So, the outcome measure could not have been influenced by knowledge of the intervention received. | Y / PY / PN / **N** / NI |
|  | 6.2 Were outcome assessors aware of the intervention received by study participants? | Yes. This is a retrospective cohort study; so, the outcome assessors were fully aware of the intervention received by study participants. | **Y** / PY / PN / N / NI |
|  | 6.3 Were the methods of outcome assessment comparable across intervention groups? | Yes. The primary outcome was a time to in-hospital death for any cause, which is a hard outcome. So, the methods of outcome assessment were comparable across intervention groups. | **Y** / PY / PN / N / NI |
|  | 6.4 Were any systematic errors in measurement of the outcome related to intervention received? | No. The primary outcome was a time to in-hospital death for any cause, which is a hard outcome. So, no systematic errors in measurement of the outcome was related to the intervention received. | Y / PY / PN / **N** / NI |
|  | **Risk of bias judgement** | **Low risk of bias:** i) The methods of outcome assessment were comparable across intervention groups AND ii) The outcome measure was unlikely to be influenced by knowledge of the intervention received by study participants AND iii) Any error in measuring the outcome is unrelated to intervention status. | **Low** / Moderate / Serious / Critical / NI |
|  | Optional: What is the predicted direction of bias due to measurement of outcomes? | Unpredictable | Favours experimental / Favours comparator / Towards null /Away from null / **Unpredictable** |

| **Bias in selection of the reported result** | | | |
| --- | --- | --- | --- |
|  | Is the reported effect estimate likely to be selected, on the basis of the results, from... |  |  |
|  | 7.1. ... multiple outcome *measurements* within the outcome domain? | No. The reported effect estimate was not likely to be selected from multiple outcome measurements. | Y / PY / PN / **N** / NI |
|  | 7.2 ... multiple *analyses* of the intervention-outcome relationship? | No. The reported effect estimate was not likely to be selected from multiple analyzed of the intervention-outcome relationship. The authors calculated the crude incidence rates using the Kaplan Meier method, and weighted HR derived from adjusted structural models. | Y / PY / PN / **N** / NI |
|  | 7.3 ... different *subgroups*? | Yes. The reported effect estimate was probably likely to be selected from different subgroups. The authors calculated subgroups analysis of the primary outcome by CRP levels (>150mg/dL or <150mg/dL). | **Y** / PY / PN / N / NI |
|  | **Risk of bias judgement** | **Serious risk of bias:** iii) The cohort or subgroup is selected from a larger study for analysis and appears to be reported on the basis of the results. | Low / Moderate / **Serious** / Critical / NI |
|  | Optional: What is the predicted direction of bias due to selection of the reported result? | Unpredictable | Favours experimental / Favours comparator / Towards null /Away from null / **Unpredictable** |

| **Overall bias** | | | |
| --- | --- | --- | --- |
|  | **Risk of bias judgement** | **Serious risk of bias:** This study was judged to have serious risk of bias in two out of seven domains. | Low / Moderate / **Serious** / Critical / NI |
|  | Optional: What is the overall predicted direction of bias for this outcome? | Unpredictable | Favours experimental / Favours comparator / Towards null /Away from null / **Unpredictable** |


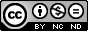


This work is licensed under a [Creative Commons Attribution-NonCommercial-NoDerivatives 4.0 International License](http://creativecommons.org/licenses/by-nc-nd/4.0/)

# ROBINS-I tool (Stage II): For each study

**Roumier M, Paule R, Vallee A, et al. Tocilizumab for severe worsening COVID-19 pneumonia: a propensity score analysis**

## Specify a target randomized trial specific to the study

| Design | Individually randomized / Cluster randomized / **Matched (e.g. cross-over)** |
| --- | --- |
| Participants | Hospitalized, adult patients with severe PCR-confirmed SARS-CoV-2 infection and high inflammatory markers |
| Experimental intervention | Tocilizumab 8mg/kg, up to 800mg IV, and a second dose 24 to 72 hours later if needed |
| Comparator | Standard of care (HCQ, steroids, azithromycin, beta-lactams, lopinavir/ritonavir) |

## Is your aim for this study…?

| **X** | **to assess the effect of *assignment to* intervention** |
| --- | --- |
| □ | to assess the effect of *starting and adhering to* intervention |

## Specify the outcome

Specify which outcome is being assessed for risk of bias (typically from among those earmarked for the Summary of Findings table). Specify whether this is a proposed benefit or harm of intervention.

| Need for ventilatory support (high-flow oxygen nasal oxygen therapy, non-invasive and invasive mechanical ventilation) at day 28 |
| --- |

## Specify the numerical result being assessed

In case of multiple alternative analyses being presented, specify the numeric result (e.g. RR = 1.52 (95% CI 0.83 to 2.77) and/or a reference (e.g. to a table, figure or paragraph) that uniquely defines the result being assessed.

| IPTW 🡪 ﻿HR: 0.39 (95%CI 0.25–0.56) |
| --- |

## Preliminary consideration of confounders

Complete a row for each important confounding domain (i) listed in the review protocol; and (ii) relevant to the setting of this particular study, or which the study authors identified as potentially important.

#### “Important” confounding domains are those for which, in the context of this study, adjustment is expected to lead to a clinically important change in the estimated effect of the intervention. “Validity” refers to whether the confounding variable or variables fully measure the domain, while “reliability” refers to the precision of the measurement (more measurement error means less reliability).

| **(i) Confounding domains listed in the review protocol** | | | | |
| --- | --- | --- | --- | --- |
| Confounding domain | Measured variable(s) | Is there evidence that controlling for this variable was unnecessary?* | Is the confounding domain measured validly and reliably by this variable (or these variables)? | OPTIONAL: Is failure to adjust for this variable (alone) expected to favour the experimental intervention or the comparator? |
| There are no confounding domains described in the protocol. |  |  |  |  |

| **(ii) Additional confounding domains relevant to the setting of this particular study, or which the study authors identified as important** | | | | |
| --- | --- | --- | --- | --- |
| Confounding domain | Measured variable(s) | Is there evidence that controlling for this variable was unnecessary?* | Is the confounding domain measured validly and reliably by this variable (or these variables)? | OPTIONAL: Is failure to adjust for this variable (alone) expected to favour the experimental intervention or the comparator? |
| Demographics | Age, male gender, BMI, hypertension, diabetes, CV disease, pulmonary disease | There is no evidence controlling was unnecessary | **Yes** / No / No information | Favour experimental / Favour comparator / **No information** |
| Baseline inflammatory markers | Mean laboratory values | There is no evidence controlling was unnecessary | **Yes** / No / No information | Favour experimental / Favour comparator / **No information** |
| Baseline severity of disease | Chest CT, clinical inclusion parameters | There is no evidence controlling was unnecessary | **Yes** / No / No information | Favour experimental / Favour comparator / **No information** |

* In the context of a particular study, variables can be demonstrated not to be confounders and so not included in the analysis: (a) if they are not predictive of the outcome; (b) if they are not predictive of intervention; or (c) because adjustment makes no or minimal difference to the estimated effect of the primary parameter. Note that “no statistically significant association” is not the same as “not predictive”.

## Preliminary consideration of co-interventions

Complete a row for each important co-intervention (i) listed in the review protocol; and (ii) relevant to the setting of this particular study, or which the study authors identified as important.

#### “Important” co-interventions are those for which, in the context of this study, adjustment is expected to lead to a clinically important change in the estimated effect of the intervention.

| **(i) Co-interventions listed in the review protocol** | | |
| --- | --- | --- |
| Co-intervention | Is there evidence that controlling for this co-intervention was unnecessary (e.g. because it was not administered)? | Is presence of this co-intervention likely to favour outcomes in the experimental intervention or the comparator |
| There are no co-interventions described in the protocol. |  |  |

| **(ii) Additional co-interventions relevant to the setting of this particular study, or which the study authors identified as important** | | |
| --- | --- | --- |
| Co-intervention | Is there evidence that controlling for this co-intervention was unnecessary (e.g. because it was not administered)? | Is presence of this co-intervention likely to favour outcomes in the experimental intervention or the comparator |
| Hydroxychloroquine | There is no evidence controlling for co-intervention was unnecessary. | Favour experimental / Favour comparator / **No information** |
| Steroids | There is no evidence controlling for co-intervention was unnecessary. | Favour experimental / Favour comparator / **No information** |
| Beta-lactams | There is no evidence controlling for co-intervention was unnecessary. | Favour experimental / Favour comparator / **No information** |
| Lopinavir/ritonavir | There is no evidence controlling for co-intervention was unnecessary. | Favour experimental / Favour comparator / **No information** |
| Azithromycin | There is no evidence controlling for co-intervention was unnecessary. | Favour experimental / Favour comparator / **No information** |

## Risk of bias assessment

Responses underlined in green are potential markers for low risk of bias, and responses in red are potential markers for a risk of bias. Where questions relate only to sign posts to other questions, no formatting is used.

|  | **Signalling questions** | **Description** | **Response options** |
| --- | --- | --- | --- |
| **Bias due to confounding** | | | |
|  | 1.1 Is there potential for confounding of the effect of intervention in this study?  **If N/PN to 1.1:** the study can be considered to be at low risk of bias due to confounding and no further signalling questions need be considered | Yes. There are potential confounders of the effect of intervention. | **Y** / PY / PN / N |
|  | **If Y/PY to 1.1**: determine whether there is a need to assess time-varying confounding: |  |  |
|  | 1.2. Was the analysis based on splitting participants’ follow up time according to intervention received?  **If N/PN**, answer questions relating to baseline confounding (1.4 to 1.6)  **If Y/PY**, go to question 1.3. | No. The intervention received did not change over time and post-baseline prognostic factors could not have affected the effect of intervention. | NA / Y / PY / PN / **N** / NI |
|  | 1.3. Were intervention discontinuations or switches likely to be related to factors that are prognostic for the outcome?  **If N/PN**, answer questions relating to baseline confounding (1.4 to 1.6)  **If Y/PY**, answer questions relating to both baseline and time-varying confounding (1.7 and 1.8) |  | NA / Y / PY / PN / N / NI |

|  | **Questions relating to baseline confounding only** | | |
| --- | --- | --- | --- |
|  | 1.4. Did the authors use an appropriate analysis method that controlled for all the important confounding domains? | Yes. The authors used inverse probability weighting and an adjusted multivariate logistic regression model to adjust for between-group differences. | NA / **Y** / PY / PN / N / NI |
|  | 1.5. **If Y/PY to 1.4**: Were confounding domains that were controlled for measured validly and reliably by the variables available in this study? | Yes. The authors measured demographic factors, clinical inclusion parameters, chest CT categories, and co-interventions validly and reliably to control confounding domains. | NA / **Y** / PY / PN / N / NI |
|  | 1.6. Did the authors control for any post-intervention variables that could have been affected by the intervention? | No. The authors did not control any post-baseline variables that could have affected the effect of intervention. | NA / Y / PY / PN / **N** / NI |
|  | **Questions relating to baseline and time-varying confounding** | | **Questions relating to baseline and time-varying confounding** |
|  | 1.7. Did the authors use an appropriate analysis method that controlled for all the important confounding domains and for time-varying confounding? | Yes. The authors used inverse probability weighting and an adjusted multivariate logistic regression model to adjust for between-group differences. | NA / **Y** / PY / PN / N / NI |
|  | 1.8. **If Y/PY to 1.7**: Were confounding domains that were controlled for measured validly and reliably by the variables available in this study? | Yes. The authors measured demographic factors, clinical inclusion parameters, chest CT categories, and co-interventions validly and reliably to control confounding domains | NA / **Y** / PY / PN / N / NI |
|  | **Risk of bias judgement** | **Moderate risk of bias:** i) Confounding expected, all known important confounding domains appropriately measured and controlled for AND ii) Reliability and validity of measurement of important domains were sufficient, such that we do not expect serious residual confounding. | Low / **Moderate** / Serious / Critical / NI |
|  | Optional: What is the predicted direction of bias due to confounding? | Unpredictable | Favours experimental / Favours comparator / **Unpredictable** |

| **Bias in selection of participants into the study** | | | |
| --- | --- | --- | --- |
|  | 2.1. Was selection of participants into the study (or into the analysis) based on participant characteristics observed after the start of intervention?  **If N/PN to 2.1:** go to 2.4 | Probably yes. Patients in the intervention group required a higher O2 flow at baseline. | Y / **PY** / PN / N / NI |
|  | 2.2. **If Y/PY to 2.1**: Were the post-intervention variables that influenced selection likely to be associated with intervention?  2.3 **If Y/PY to 2.2**: Were the post-intervention variables that influenced selection likely to be influenced by the outcome or a cause of the outcome? | Probably yes. The higher oxygen requirement at baseline of participants in the intervention group could have influenced in their selection.  Probably yes. The higher oxygen requirement at baseline of participants in the intervention group could have influenced in their outcomes, since it was time to need of ventilatory support at 28 days. | NA / Y / **PY** / PN / N / NI  NA / Y / **PY** / PN / N / NI |
|  | 2.4. Do start of follow-up and start of intervention coincide for most participants? | Probably no. The median time of duration of symptoms before study inclusion was 9 days. | Y / PY / **PN** / N / NI |
|  | 2.5. **If Y/PY to 2.2 and 2.3, or N/PN to 2.4**: Were adjustment techniques used that are likely to correct for the presence of selection biases? | Yes. The authors used inverse probability weighting to correct for the presence of selection biases. | NA / **Y** / PY / PN / N / NI |
|  | **Risk of bias judgement** | **Serious risk of bias:** i) Selection into the study was related to intervention and outcome AND This could not be adjusted for in analyses. | Low / Moderate / **Serious** / Critical / NI |
|  | Optional: What is the predicted direction of bias due to selection of participants into the study? | Unpredictable | Favours experimental / Favours comparator / Towards null /Away from null / **Unpredictable** |

| **Bias in classification of interventions** | | | |
| --- | --- | --- | --- |
|  | 3.1 Were intervention groups clearly defined? | Yes. The intervention and control groups were clearly defined. | **Y** / PY / PN / N / NI |
|  | 3.2 Was the information used to define intervention groups recorded at the start of the intervention? | Probably yes. The study was conducted in France. They probably used Electronic Medical Records (EMR) to define the start of the intervention. | Y / **PY** / PN / N / NI |
|  | 3.3 Could classification of intervention status have been affected by knowledge of the outcome or risk of the outcome? | Probably no. Classification of the intervention status was done retrospectively, but EMR probably contributed to reduce misclassification | Y / PY / **PN** / N / NI |
|  | **Risk of bias judgement** | **Moderate risk of bias:** i) Intervention status is well defined AND ii) Some aspects of the assignments of intervention status were done retrospectively. | Low / **Moderate** / Serious / Critical / NI |
|  | Optional: What is the predicted direction of bias due to classification of interventions? | Unpredictable | Favours experimental / Favours comparator / Towards null /Away from null / **Unpredictable** |

| **Bias due to deviations from intended interventions** | | | |
| --- | --- | --- | --- |
|  | **If your aim for this study is to assess the effect of assignment to intervention, answer questions 4.1 and 4.2** | |  |
|  | 4.1. Were there deviations from the intended intervention beyond what would be expected in usual practice? | No. There were no deviations from the intended intervention. | Y / PY / PN / **N** / NI |
|  | 4.2. **If Y/PY to 4.1**: Were these deviations from intended intervention unbalanced between groups *and* likely to have affected the outcome? |  | NA / Y / PY / PN / N / NI |
|  | **If your aim for this study is to assess the effect of starting and adhering to intervention, answer questions 4.3 to 4.6** | | **If your aim for this study is to assess the effect of starting and adhering to intervention, answer questions 4.3 to 4.6** |
|  | 4.3. Were important co-interventions balanced across intervention groups? | No. There are some disbalances across the intervention groups, specifically in lopinavir/ritonavir and azithromycin | Y / PY / PN / **N** / NI |
|  | 4.4. Was the intervention implemented successfully for most participants? | Yes. The intervention was implemented successfully for all participants. | **Y** / PY / PN / N / NI |
|  | 4.5. Did study participants adhere to the assigned intervention regimen? | Yes. Study participants adhered to the assigned intervention regimen. | **Y** / PY / PN / N / NI |
|  | 4.6. **If N/PN to 4.3, 4.4 or 4.5**: Was an appropriate analysis used to estimate the effect of starting and adhering to the intervention? | Yes. The authors used IPTW analysis to estimate the effect of starting and adhering to the intervention. | NA / **Y** / PY / PN / N / NI |
|  | **Risk of bias judgement** | **Moderate risk of bias:** ii) The important co-interventions were not balanced across the intervention groups. | Low / **Moderate** / Serious / Critical / NI |
|  | Optional: What is the predicted direction of bias due to deviations from the intended interventions? | Unpredictable | Favours experimental / Favours comparator / Towards null /Away from null / **Unpredictable** |

| **Bias due to missing data** | | | |
| --- | --- | --- | --- |
|  | 5.1 Were outcome data available for all, or nearly all, participants? | Yes. Outcome data was available for nearly all participants | **Y** / PY / PN / N / NI |
|  | 5.2 Were participants excluded due to missing data on intervention status? | No. Participants were not excluded due to missing data on the intervention status. | Y / PY / PN / **N** / NI |
|  | 5.3 Were participants excluded due to missing data on other variables needed for the analysis? | No. Participants were not excluded due to missing data on other variables needed for the analysis. | Y / PY / PN / **N** / NI |
|  | 5.4 **If PN/N to 5.1, or Y/PY to 5.2 or 5.3**: Are the proportion of participants and reasons for missing data similar across interventions? |  | NA / Y / PY / PN / N / NI |
|  | 5.5 **If PN/N to 5.1, or Y/PY to 5.2 or 5.3**: Is there evidence that results were robust to the presence of missing data? |  | NA / Y / PY / PN / N / NI |
|  | **Risk of bias judgement** | **Low risk of bias:** i) Data were reasonable complete. | **Low** / Moderate / Serious / Critical / NI |
|  | Optional: What is the predicted direction of bias due to missing data? | Unpredictable | Favours experimental / Favours comparator / Towards null /Away from null / **Unpredictable** |

| **Bias in measurement of outcomes** | | | |
| --- | --- | --- | --- |
|  | 6.1 Could the outcome measure have been influenced by knowledge of the intervention received? | No. The primary outcome was a need for oxygen support, which is a hard outcome. So, the outcome measure could not have been influenced by knowledge of the intervention received. | Y / PY / PN / **N** / NI |
|  | 6.2 Were outcome assessors aware of the intervention received by study participants? | Yes. This is a retrospective cohort study; so, the outcome assessors were fully aware of the intervention received by study participants. | **Y** / PY / PN / N / NI |
|  | 6.3 Were the methods of outcome assessment comparable across intervention groups? | Yes. The primary outcome was a need for oxygen support, which is a hard outcome. So, the methods of outcome assessment were comparable across intervention groups. | **Y** / PY / PN / N / NI |
|  | 6.4 Were any systematic errors in measurement of the outcome related to intervention received? | No. The primary outcome was a need for oxygen support, which is a hard outcome. So, no systematic errors in measurement of the outcome was related to the intervention received. | Y / PY / PN / **N** / NI |
|  | **Risk of bias judgement** | **Low risk of bias:** i) The methods of outcome assessment were comparable across intervention groups AND ii) The outcome measure was unlikely to be influenced by knowledge of the intervention received by study participants AND iii) Any error in measuring the outcome is unrelated to intervention status. | **Low** / Moderate / Serious / Critical / NI |
|  | Optional: What is the predicted direction of bias due to measurement of outcomes? | Unpredictable | Favours experimental / Favours comparator / Towards null /Away from null / **Unpredictable** |

| **Bias in selection of the reported result** | | | |
| --- | --- | --- | --- |
|  | Is the reported effect estimate likely to be selected, on the basis of the results, from... |  |  |
|  | 7.1. ... multiple outcome *measurements* within the outcome domain? | No. The reported effect estimate was not likely to be selected from multiple outcome measurements. | Y / PY / PN / **N** / NI |
|  | 7.2 ... multiple *analyses* of the intervention-outcome relationship? | No. The reported effect estimate was not likely to be selected from multiple analyzed of the intervention-outcome relationship. The authors used inverse probability weighting and an adjusted multivariate logistic regression model, and calculated the unadjusted and weighted two-sided log-rank test and two-sided HR based on a Cox proportional hazard-model and Kaplan-Meier survival curves. | Y / PY / PN / **N** / NI |
|  | 7.3 ... different *subgroups*? | No. The reported effect estimate was not likely to be selected from different subgroups. | Y / PY / PN / **N** / NI |
|  | **Risk of bias judgement** | **Low risk of bias:** There is clear evidence that all reported results correspond to all intended outcome, analysis, and sub-cohorts | **Low** / Moderate / Serious / Critical / NI |
|  | Optional: What is the predicted direction of bias due to selection of the reported result? | Unpredictable | Favours experimental / Favours comparator / Towards null /Away from null / **Unpredictable** |

| **Overall bias** | | | |
| --- | --- | --- | --- |
|  | **Risk of bias judgement** | **Serious risk of bias:** This study was judged to have serious risk of bias in one out of seven domains. | Low / Moderate / **Serious** / Critical / NI |
|  | Optional: What is the overall predicted direction of bias for this outcome? | Unpredictable | Favours experimental / Favours comparator / Towards null /Away from null / **Unpredictable** |

# ROBINS-I tool (Stage II): For each study

**Gupta S, et al. ﻿ Association between early treatment with Tocilizumab and Mortality among critically ill patients with COVID-19. ﻿Jama Internal Medicine. 2020. doi:10.1001/jamainternmed.2020.6252**

## Specify a target randomized trial specific to the study

| Design | Individually randomized / Cluster randomized / **Matched (e.g. cross-over)** |
| --- | --- |
| Participants | Hospitalized, adult, severe PCR-confirmed SARS-CoV-2 infection admitted to the ICU |
| Experimental intervention | Tocilizumab |
| Comparator | Standard of care (HCQ, steroids, azithromycin, anticoagulation, neuromuscular blockade, prone positioning) |

## Is your aim for this study…?

| **X** | **to assess the effect of *assignment to* intervention** |
| --- | --- |
| □ | to assess the effect of *starting and adhering to* intervention |

## Specify the outcome

Specify which outcome is being assessed for risk of bias (typically from among those earmarked for the Summary of Findings table). Specify whether this is a proposed benefit or harm of intervention.

| In-hospital death |
| --- |

## Specify the numerical result being assessed

In case of multiple alternative analyses being presented, specify the numeric result (e.g. RR = 1.52 (95% CI 0.83 to 2.77) and/or a reference (e.g. to a table, figure or paragraph) that uniquely defines the result being assessed.

| HR 0.71 (95%CI 0.56-0.92) |
| --- |

## Preliminary consideration of confounders

Complete a row for each important confounding domain (i) listed in the review protocol; and (ii) relevant to the setting of this particular study, or which the study authors identified as potentially important.

#### “Important” confounding domains are those for which, in the context of this study, adjustment is expected to lead to a clinically important change in the estimated effect of the intervention. “Validity” refers to whether the confounding variable or variables fully measure the domain, while “reliability” refers to the precision of the measurement (more measurement error means less reliability).

| **(i) Confounding domains listed in the review protocol** | | | | |
| --- | --- | --- | --- | --- |
| Confounding domain | Measured variable(s) | Is there evidence that controlling for this variable was unnecessary?* | Is the confounding domain measured validly and reliably by this variable (or these variables)? | OPTIONAL: Is failure to adjust for this variable (alone) expected to favour the experimental intervention or the comparator? |
| There are no confounding domains described in the protocol. |  |  |  |  |

| **(ii) Additional confounding domains relevant to the setting of this particular study, or which the study authors identified as important** | | | | |
| --- | --- | --- | --- | --- |
| Confounding domain | Measured variable(s) | Is there evidence that controlling for this variable was unnecessary?* | Is the confounding domain measured validly and reliably by this variable (or these variables)? | OPTIONAL: Is failure to adjust for this variable (alone) expected to favour the experimental intervention or the comparator? |
| Demographics | Age, sex, race, ethnicity, comorbidities, home medications | There is no evidence controlling was unnecessary | **Yes** / No / No information | Favour experimental / Favour comparator / **No information** |
| Baseline inflammatory markers | Fever, renal SOFA, liver SOFA, PaFiO2 | There is no evidence controlling was unnecessary | **Yes** / No / No information | Favour experimental / Favour comparator / **No information** |
| Baseline severity of disease | Time from symptom onset to ICU admission | There is no evidence controlling was unnecessary | **Yes** / No / No information | Favour experimental / Favour comparator / **No information** |

* In the context of a particular study, variables can be demonstrated not to be confounders and so not included in the analysis: (a) if they are not predictive of the outcome; (b) if they are not predictive of intervention; or (c) because adjustment makes no or minimal difference to the estimated effect of the primary parameter. Note that “no statistically significant association” is not the same as “not predictive”.

## Preliminary consideration of co-interventions

Complete a row for each important co-intervention (i) listed in the review protocol; and (ii) relevant to the setting of this particular study, or which the study authors identified as important.

#### “Important” co-interventions are those for which, in the context of this study, adjustment is expected to lead to a clinically important change in the estimated effect of the intervention.

| **(i) Co-interventions listed in the review protocol** | | |
| --- | --- | --- |
| Co-intervention | Is there evidence that controlling for this co-intervention was unnecessary (e.g. because it was not administered)? | Is presence of this co-intervention likely to favour outcomes in the experimental intervention or the comparator |
| There are no co-interventions described in the protocol. |  |  |

| **(ii) Additional co-interventions relevant to the setting of this particular study, or which the study authors identified as important** | | |
| --- | --- | --- |
| Co-intervention | Is there evidence that controlling for this co-intervention was unnecessary (e.g. because it was not administered)? | Is presence of this co-intervention likely to favour outcomes in the experimental intervention or the comparator |
| Steroids | There is no evidence controlling for co-intervention was unnecessary. | Favour experimental / Favour comparator / **No information** |
| Hydroxychloroquine | There is no evidence controlling for co-intervention was unnecessary. | Favour experimental / Favour comparator / **No information** |
| Azithromycin | There is no evidence controlling for co-intervention was unnecessary. | Favour experimental / Favour comparator / **No information** |
| Anticoagulation | There is no evidence controlling for co-intervention was unnecessary. | Favour experimental / Favour comparator / **No information** |
| Neuromuscular blockade | There is no evidence controlling for co-intervention was unnecessary. | Favour experimental / Favour comparator / **No information** |

## Risk of bias assessment

Responses underlined in green are potential markers for low risk of bias, and responses in red are potential markers for a risk of bias. Where questions relate only to sign posts to other questions, no formatting is used.

|  | **Signalling questions** | **Description** | **Response options** |
| --- | --- | --- | --- |
| **Bias due to confounding** | | | |
|  | 1.1 Is there potential for confounding of the effect of intervention in this study?  **If N/PN to 1.1:** the study can be considered to be at low risk of bias due to confounding and no further signalling questions need be considered | Yes. There are potential confounders of the effect of intervention. | **Y** / PY / PN / N |
|  | **If Y/PY to 1.1**: determine whether there is a need to assess time-varying confounding: |  |  |
|  | 1.2. Was the analysis based on splitting participants’ follow up time according to intervention received?  **If N/PN**, answer questions relating to baseline confounding (1.4 to 1.6)  **If Y/PY**, go to question 1.3. | No. The intervention received did not change over time and post-baseline prognostic factors could not have affected the effect of intervention. | NA / Y / PY / PN / **N** / NI |
|  | 1.3. Were intervention discontinuations or switches likely to be related to factors that are prognostic for the outcome?  **If N/PN**, answer questions relating to baseline confounding (1.4 to 1.6)  **If Y/PY**, answer questions relating to both baseline and time-varying confounding (1.7 and 1.8) |  | NA / Y / PY / PN / N / NI |

|  | **Questions relating to baseline confounding only** | | |
| --- | --- | --- | --- |
|  | 1.4. Did the authors use an appropriate analysis method that controlled for all the important confounding domains? | Yes. The authors used inverse probability weighting and a Cox regression model. | NA / **Y** / PY / PN / N / NI |
|  | 1.5. **If Y/PY to 1.4**: Were confounding domains that were controlled for measured validly and reliably by the variables available in this study? | Yes. The authors measured demographic factors, baseline markers and baseline severity of disease. | NA / **Y** / PY / PN / N / NI |
|  | 1.6. Did the authors control for any post-intervention variables that could have been affected by the intervention? | No. The authors did not control any post-baseline variables that could have affected the effect of intervention. | NA / Y / PY / PN / **N** / NI |
|  | **Questions relating to baseline and time-varying confounding** | | **Questions relating to baseline and time-varying confounding** |
|  | 1.7. Did the authors use an appropriate analysis method that controlled for all the important confounding domains and for time-varying confounding? | Yes. The authors used inverse probability weighting and an adjusted multivariate logistic regression model to adjust for between-group differences. | NA / **Y** / PY / PN / N / NI |
|  | 1.8. **If Y/PY to 1.7**: Were confounding domains that were controlled for measured validly and reliably by the variables available in this study? | Yes. The authors measured demographic factors, baseline markers and baseline severity of disease, and co-interventions validly and reliably to control confounding domains | NA / **Y** / PY / PN / N / NI |
|  | **Risk of bias judgement** | **Moderate risk of bias:** i) Confounding expected, all known important confounding domains appropriately measured and controlled for AND ii) Reliability and validity of measurement of important domains were sufficient, such that we do not expect serious residual confounding. | Low / **Moderate** / Serious / Critical / NI |
|  | Optional: What is the predicted direction of bias due to confounding? | Unpredictable | Favours experimental / Favours comparator / **Unpredictable** |

| **Bias in selection of participants into the study** | | | |
| --- | --- | --- | --- |
|  | 2.1. Was selection of participants into the study (or into the analysis) based on participant characteristics observed after the start of intervention?  **If N/PN to 2.1:** go to 2.4 | No. There was no selection of participants into the study based on participants characteristics observed after the start of the intervention. | Y / **PY** / PN / **N** / NI |
|  | 2.2. **If Y/PY to 2.1**: Were the post-intervention variables that influenced selection likely to be associated with intervention?  2.3 **If Y/PY to 2.2**: Were the post-intervention variables that influenced selection likely to be influenced by the outcome or a cause of the outcome? |  | NA / Y / PY / PN / N / NI  NA / Y / PY / PN / N / NI |
|  | 2.4. Do start of follow-up and start of intervention coincide for most participants? | No. Per Table 1, most of the participants in both groups were admitted to the ICU after 3 days of symptoms onset. | Y / PY / PN / **N** / NI |
|  | 2.5. **If Y/PY to 2.2 and 2.3, or N/PN to 2.4**: Were adjustment techniques used that are likely to correct for the presence of selection biases? | Yes. The authors used inverse probability weighting to correct for the presence of selection biases. | NA / **Y** / PY / PN / N / NI |
|  | **Risk of bias judgement** | **Moderate risk of bias:** i) Start of follow up of intervention do not coincide for all participants AND b) The authors used appropriate methods to adjust for the selection bias. | Low / Moderate / **Serious** / Critical / NI |
|  | Optional: What is the predicted direction of bias due to selection of participants into the study? | Unpredictable | Favours experimental / Favours comparator / Towards null /Away from null / **Unpredictable** |

| **Bias in classification of interventions** | | | |
| --- | --- | --- | --- |
|  | 3.1 Were intervention groups clearly defined? | Yes. The intervention and control groups were clearly defined. | **Y** / PY / PN / N / NI |
|  | 3.2 Was the information used to define intervention groups recorded at the start of the intervention? | Yes. The data was from STOP-COVID, a multicenter cohort study of ICU of 68 hospitals in the US. They probably used Electronic Medical Records (EMR) to define the start of the intervention. | **Y** / PY / PN / N / NI |
|  | 3.3 Could classification of intervention status have been affected by knowledge of the outcome or risk of the outcome? | Probably no. Classification of the intervention status was done retrospectively, but EMR contributed to reduce misclassification. | Y / PY / **PN** / N / NI |
|  | **Risk of bias judgement** | **Moderate risk of bias:** i) Intervention status is well defined AND ii) Some aspects of the assignments of intervention status were done retrospectively. | Low / **Moderate** / Serious / Critical / NI |
|  | Optional: What is the predicted direction of bias due to classification of interventions? | Unpredictable | Favours experimental / Favours comparator / Towards null /Away from null / **Unpredictable** |

| **Bias due to deviations from intended interventions** | | | |
| --- | --- | --- | --- |
|  | **If your aim for this study is to assess the effect of assignment to intervention, answer questions 4.1 and 4.2** | |  |
|  | 4.1. Were there deviations from the intended intervention beyond what would be expected in usual practice? | No. There were no deviations from the intended intervention. | Y / PY / PN / **N** / NI |
|  | 4.2. **If Y/PY to 4.1**: Were these deviations from intended intervention unbalanced between groups *and* likely to have affected the outcome? |  | NA / Y / PY / PN / N / NI |
|  | **If your aim for this study is to assess the effect of starting and adhering to intervention, answer questions 4.3 to 4.6** | | **If your aim for this study is to assess the effect of starting and adhering to intervention, answer questions 4.3 to 4.6** |
|  | 4.3. Were important co-interventions balanced across intervention groups? | No. There are some disbalances across the intervention groups. | Y / PY / PN / **N** / NI |
|  | 4.4. Was the intervention implemented successfully for most participants? | Yes. The intervention was implemented successfully for all participants. | **Y** / PY / PN / N / NI |
|  | 4.5. Did study participants adhere to the assigned intervention regimen? | Yes. Study participants adhered to the assigned intervention regimen. | **Y** / PY / PN / N / NI |
|  | 4.6. **If N/PN to 4.3, 4.4 or 4.5**: Was an appropriate analysis used to estimate the effect of starting and adhering to the intervention? | Yes. The authors used IPTW analysis to estimate the effect of starting and adhering to the intervention. | NA / **Y** / PY / PN / N / NI |
|  | **Risk of bias judgement** | **Moderate risk of bias:** ii) The important co-interventions were not balanced across the intervention groups. | Low / **Moderate** / Serious / Critical / NI |
|  | Optional: What is the predicted direction of bias due to deviations from the intended interventions? | Unpredictable | Favours experimental / Favours comparator / Towards null /Away from null / **Unpredictable** |

| **Bias due to missing data** | | | |
| --- | --- | --- | --- |
|  | 5.1 Were outcome data available for all, or nearly all, participants? | Yes. Outcome data was available for all participants. | **Y** / PY / PN / N / NI |
|  | 5.2 Were participants excluded due to missing data on intervention status? | No. Participants were not excluded due to missing data on the intervention status. | Y / PY / PN / **N** / NI |
|  | 5.3 Were participants excluded due to missing data on other variables needed for the analysis? | No. Participants were not excluded due to missing data on other variables needed for the analysis. | Y / PY / PN / **N** / NI |
|  | 5.4 **If PN/N to 5.1, or Y/PY to 5.2 or 5.3**: Are the proportion of participants and reasons for missing data similar across interventions? |  | NA / Y / PY / PN / N / NI |
|  | 5.5 **If PN/N to 5.1, or Y/PY to 5.2 or 5.3**: Is there evidence that results were robust to the presence of missing data? |  | NA / Y / PY / PN / N / NI |
|  | **Risk of bias judgement** | **Low risk of bias:** i) Data were reasonable complete. | **Low** / Moderate / Serious / Critical / NI |
|  | Optional: What is the predicted direction of bias due to missing data? | Unpredictable | Favours experimental / Favours comparator / Towards null /Away from null / **Unpredictable** |

| **Bias in measurement of outcomes** | | | |
| --- | --- | --- | --- |
|  | 6.1 Could the outcome measure have been influenced by knowledge of the intervention received? | No. The primary outcome was a in-hospital death, which is a hard outcome. So, the outcome measure could not have been influenced by knowledge of the intervention received. | Y / PY / PN / **N** / NI |
|  | 6.2 Were outcome assessors aware of the intervention received by study participants? | Yes. This is a retrospective cohort study; so, the outcome assessors were fully aware of the intervention received by study participants. | **Y** / PY / PN / N / NI |
|  | 6.3 Were the methods of outcome assessment comparable across intervention groups? | Yes. The primary outcome was a in-hospital death, which is a hard outcome. So, the methods of outcome assessment were comparable across intervention groups. | **Y** / PY / PN / N / NI |
|  | 6.4 Were any systematic errors in measurement of the outcome related to intervention received? | No. The primary outcome was a in-hospital death, which is a hard outcome. So, no systematic errors in measurement of the outcome was related to the intervention received. | Y / PY / PN / **N** / NI |
|  | **Risk of bias judgement** | **Low risk of bias:** i) The methods of outcome assessment were comparable across intervention groups AND ii) The outcome measure was unlikely to be influenced by knowledge of the intervention received by study participants AND iii) Any error in measuring the outcome is unrelated to intervention status. | **Low** / Moderate / Serious / Critical / NI |
|  | Optional: What is the predicted direction of bias due to measurement of outcomes? | Unpredictable | Favours experimental / Favours comparator / Towards null /Away from null / **Unpredictable** |

| **Bias in selection of the reported result** | | | |
| --- | --- | --- | --- |
|  | Is the reported effect estimate likely to be selected, on the basis of the results, from... |  |  |
|  | 7.1. ... multiple outcome *measurements* within the outcome domain? | No. The reported effect estimate was not likely to be selected from multiple outcome measurements. | Y / PY / PN / **N** / NI |
|  | 7.2 ... multiple *analyses* of the intervention-outcome relationship? | No. The reported effect estimate was not likely to be selected from multiple analyzed of the intervention-outcome relationship.  The authors used inverse probability weighting and a Cox regression model. They performed multiple analyses per Figure 3, but presented them all. | Y / PY / PN / **N** / NI |
|  | 7.3 ... different *subgroups*? | No. The reported effect estimate was not likely to be selected from different subgroups.  The authors performed multiple analyses per Figure 3, but presented them all. | Y / PY / PN / **N** / NI |
|  | **Risk of bias judgement** | **Low risk of bias:** There is clear evidence that all reported results correspond to all intended outcome, analysis, and sub-cohorts. | **Low** / Moderate / Serious / Critical / NI |
|  | Optional: What is the predicted direction of bias due to selection of the reported result? | Unpredictable | Favours experimental / Favours comparator / Towards null /Away from null / **Unpredictable** |

| **Overall bias** | | | |
| --- | --- | --- | --- |
|  | **Risk of bias judgement** | **Moderate risk of bias:** This study was judged to have serious risk of bias in one out of seven domains. | Low / **Moderate** / Serious / Critical / NI |
|  | Optional: What is the overall predicted direction of bias for this outcome? | Unpredictable | Favours experimental / Favours comparator / Towards null /Away from null / **Unpredictable** |

# ROBINS-I tool (Stage II): For each study

**Biran et al. Tocilizumab among patients COVID-19 in the intensive care unit: a multicentre observational study. Lancet Rheumatol 2020;2;e603-12**

## Specify a target randomized trial specific to the study

| Design | Individually randomized / Cluster randomized / Matched (e.g. cross-over) |
| --- | --- |
| Participants | Adult Patients with SARS COV-2 by RT PCR who received ICU support |
| Experimental intervention | Tocilizumab (400mg IV 1 dose, 2^nd^ dose if clinical worsening) |
| Comparator | Standard of care |

## Is your aim for this study…?

| 🞩 | to assess the effect of *assignment to* intervention |
| --- | --- |
| □ | to assess the effect of *starting and adhering to* intervention |

## Specify the outcome

Specify which outcome is being assessed for risk of bias (typically from among those earmarked for the Summary of Findings table). Specify whether this is a proposed benefit or harm of intervention.

| Hospital-related mortality |
| --- |

## Specify the numerical result being assessed

In case of multiple alternative analyses being presented, specify the numeric result (e.g. RR = 1.52 (95% CI 0.83 to 2.77) and/or a reference (e.g. to a table, figure or paragraph) that uniquely defines the result being assessed.

| 102/210 vs 256/420, RR= 0.80 (95% CI 0.68-0.93) |
| --- |

## Preliminary consideration of confounders

Complete a row for each important confounding domain (i) listed in the review protocol; and (ii) relevant to the setting of this particular study, or which the study authors identified as potentially important.

#### “Important” confounding domains are those for which, in the context of this study, adjustment is expected to lead to a clinically important change in the estimated effect of the intervention. “Validity” refers to whether the confounding variable or variables fully measure the domain, while “reliability” refers to the precision of the measurement (more measurement error means less reliability).

| **(i) Confounding domains listed in the review protocol** | | | | |
| --- | --- | --- | --- | --- |
| Confounding domain | Measured variable(s) | Is there evidence that controlling for this variable was unnecessary?* | Is the confounding domain measured validly and reliably by this variable (or these variables)? | OPTIONAL: Is failure to adjust for this variable (alone) expected to favour the experimental intervention or the comparator? |
| There are no confounding domains described in the protocol. |  |  | Yes / No / No information | Favour experimental / Favour comparator / No information |

| **(ii) Additional confounding domains relevant to the setting of this particular study, or which the study authors identified as important** | | | | |
| --- | --- | --- | --- | --- |
| Confounding domain | Measured variable(s) | Is there evidence that controlling for this variable was unnecessary?* | Is the confounding domain measured validly and reliably by this variable (or these variables)? | OPTIONAL: Is failure to adjust for this variable (alone) expected to favour the experimental intervention or the comparator? |
| Demographics | Age, male gender, race, BMI, obesity hypertension, diabetes, CV disease, pulmonary disease, smoking status | There is no evidence controlling was unnecessary | **Yes** / No / No information | Favour experimental / Favour comparator / **No information** |
| Baseline inflammatory markers | CRP, D-Dimer, IL-6, ferritin | There is no evidence controlling was unnecessary | **Yes** / No / No information | Favour experimental / Favour comparator / **No information** |
| Baseline severity of disease | PaFiO2, PEEP, Intubation or ventilator, Vasopressor | There is no evidence controlling was unnecessary | **Yes** / No / No information | Favour experimental / Favour comparator / **No information** |

* In the context of a particular study, variables can be demonstrated not to be confounders and so not included in the analysis: (a) if they are not predictive of the outcome; (b) if they are not predictive of intervention; or (c) because adjustment makes no or minimal difference to the estimated effect of the primary parameter. Note that “no statistically significant association” is not the same as “not predictive”.

## Preliminary consideration of co-interventions

Complete a row for each important co-intervention (i) listed in the review protocol; and (ii) relevant to the setting of this particular study, or which the study authors identified as important.

#### “Important” co-interventions are those for which, in the context of this study, adjustment is expected to lead to a clinically important change in the estimated effect of the intervention.

| **(i) Co-interventions listed in the review protocol** | | |
| --- | --- | --- |
| Co-intervention | Is there evidence that controlling for this co-intervention was unnecessary (e.g. because it was not administered)? | Is presence of this co-intervention likely to favour outcomes in the experimental intervention or the comparator |
| There are no co-interventions described in the protocol. |  | Favour experimental / Favour comparator / No information |
|  |  | Favour experimental / Favour comparator / No information |

| **(ii) Additional co-interventions relevant to the setting of this particular study, or which the study authors identified as important** | | |
| --- | --- | --- |
| Co-intervention | Is there evidence that controlling for this co-intervention was unnecessary (e.g. because it was not administered)? | Is presence of this co-intervention likely to favour outcomes in the experimental intervention or the comparator |
| Steroids | There is no evidence controlling for co-intervention was unnecessary. | Favour experimental / Favour comparator / **No information** |
| Hydroxychloroquine | There is no evidence controlling for co-intervention was unnecessary. | Favour experimental / Favour comparator / **No information** |
| Antibiotics | There is no evidence controlling for co-intervention was unnecessary. | Favour experimental / Favour comparator / **No information** |

## Risk of bias assessment

Responses underlined in green are potential markers for low risk of bias, and responses in red are potential markers for a risk of bias. Where questions relate only to sign posts to other questions, no formatting is used.

|  | **Signalling questions** | **Description** | **Response options** |
| --- | --- | --- | --- |
| **Bias due to confounding** | | | |
|  | 1.1 Is there potential for confounding of the effect of intervention in this study?  **If N/PN to 1.1:** the study can be considered to be at low risk of bias due to confounding and no further signalling questions need be considered | Yes. There are potential confounders of the effect of intervention. | **Y** / PY / PN / N |
|  | **If Y/PY to 1.1**: determine whether there is a need to assess time-varying confounding: |  |  |
|  | 1.2. Was the analysis based on splitting participants’ follow up time according to intervention received?  **If N/PN**, answer questions relating to baseline confounding (1.4 to 1.6)  **If Y/PY**, go to question 1.3. | No. The intervention received did not change over time and post-baseline prognostic factors could not have affected the effect of intervention. | NA / Y / PY / PN / **N** / NI |
|  | 1.3. Were intervention discontinuations or switches likely to be related to factors that are prognostic for the outcome?  **If N/PN**, answer questions relating to baseline confounding (1.4 to 1.6)  **If Y/PY**, answer questions relating to both baseline and time-varying confounding (1.7 and 1.8) |  | NA / Y / PY / PN / N / NI |

|  | **Questions relating to baseline confounding only** | | |
| --- | --- | --- | --- |
|  | 1.4. Did the authors use an appropriate analysis method that controlled for all the important confounding domains? | Yes. The authors used propensity score matching and calculated it using multivariable logistic regression with the confounders age, gender, diabetes, COPD, HT, cancer, renal failure, obesity, O2 <94%, qSOPA score, use of steroids, CPR>15, intubation. Goodness of fit of the multivariable logistic model was examined using the Hosmer Lemeshow test. They used a matched cohort in a 1:2 ratio to pair a patient with tocilizumab treatment to two patients who did not receive tocilizumab, using the MatchIt package in R. | NA / **Y** / PY / PN / N / NI |
|  | 1.5. **If Y/PY to 1.4**: Were confounding domains that were controlled for measured validly and reliably by the variables available in this study? | No. The authors measured demographic factors, clinical factors, laboratory tests, radiological tests, and medications validly and reliably to control confounding domains; but they did not measured clinical status at baseline to control for baseline severity of disease. | NA / Y / PY / PN / **N** / NI |
|  | 1.6. Did the authors control for any post-intervention variables that could have been affected by the intervention? | No. The authors did not control any post-baseline variables that could have affected the effect of intervention. | NA / Y / PY / PN / **N** / NI |
|  | **Questions relating to baseline and time-varying confounding** | |  |
|  | 1.7. Did the authors use an appropriate analysis method that controlled for all the important confounding domains and for time-varying confounding? | Yes. The authors used propensity score matching, adjusted Cox modelling to control the confounding domains and time-varying confounding. | NA / **Y** / PY / PN / N / NI |
|  | 1.8. **If Y/PY to 1.7**: Were confounding domains that were controlled for measured validly and reliably by the variables available in this study? | Yes. The authors measured demographic factors, clinical factors, laboratory tests and medications validly and reliably to control confounding domains; but they did not measured clinical status at baseline to control for baseline severity of disease. | NA / **Y** / PY / PN / N / NI |
|  | **Risk of bias judgement** | **Serious risk of bias:** i) At least one known important domain was not appropriately measured, or not controlled for. | Low / Moderate / **Serious** / Critical / NI |
|  | Optional: What is the predicted direction of bias due to confounding? | Unpredictable | Favours experimental / Favours comparator / **Unpredictable** |

| **Bias in selection of participants into the study** | | | |
| --- | --- | --- | --- |
|  | 2.1. Was selection of participants into the study (or into the analysis) based on participant characteristics observed after the start of intervention?  **If N/PN to 2.1:** go to 2.4 | No. The selection of participants into the study was not based on participants characteristics observed after the start of the intervention. | Y / PY / PN / **N** / NI |
|  | 2.2. **If Y/PY to 2.1**: Were the post-intervention variables that influenced selection likely to be associated with intervention?  2.3 **If Y/PY to 2.2**: Were the post-intervention variables that influenced selection likely to be influenced by the outcome or a cause of the outcome? |  | NA / Y / PY / PN / N / NI  NA / Y / PY / PN / N / NI |
|  | 2.4. Do start of follow-up and start of intervention coincide for most participants? | No. There are specific variables on Table 1 measuring the delay between the first symptoms and hospital admission, and study inclusion. | Y / PY / PN / **N** / NI |
|  | 2.5. **If Y/PY to 2.2 and 2.3, or N/PN to 2.4**: Were adjustment techniques used that are likely to correct for the presence of selection biases? | Yes. The authors used inverse probability weighting to correct for the presence of selection biases. | NA / **Y** / PY / PN / N / NI |
|  | **Risk of bias judgement** | **Moderate risk of bias:** ii) Start of the follow-up and start of the intervention do not coincide for all participants AND a) The authors used appropriate methods to adjust for the selection bias. | Low / **Moderate** / Serious / Critical / NI |
|  | Optional: What is the predicted direction of bias due to selection of participants into the study? | Unpredictable | Favours experimental / Favours comparator / Towards null /Away from null / **Unpredictable** |

| **Bias in classification of interventions** | | | |
| --- | --- | --- | --- |
|  | 3.1 Were intervention groups clearly defined? | Yes. The intervention and control groups were clearly defined. | **Y** / PY / PN / N / NI |
|  | 3.2 Was the information used to define intervention groups recorded at the start of the intervention? | Yes. The study was conducted in the US and they used Electronic Medical Records (EMR) to define the start of the intervention. | **Y** / PY / PN / N / NI |
|  | 3.3 Could classification of intervention status have been affected by knowledge of the outcome or risk of the outcome? | Probably no. Classification of the intervention status was done retrospectively according to EMR, so misclassification was not likely. | Y / PY / **PN** / N / NI |
|  | **Risk of bias judgement** | **Moderate risk of bias:** i) Intervention status is well defined AND ii) Some aspects of the assignments of intervention status were done retrospectively. | Low / **Moderate** / Serious / Critical / NI |
|  | Optional: What is the predicted direction of bias due to classification of interventions? | Unpredictable | Favours experimental / Favours comparator / Towards null /Away from null / **Unpredictable** |

| **Bias due to deviations from intended interventions** | | | |
| --- | --- | --- | --- |
|  | **If your aim for this study is to assess the effect of assignment to intervention, answer questions 4.1 and 4.2** | |  |
|  | 4.1. Were there deviations from the intended intervention beyond what would be expected in usual practice? | No. There were no deviations from the intended intervention. | Y / PY / PN / **N** / NI |
|  | 4.2. **If Y/PY to 4.1**: Were these deviations from intended intervention unbalanced between groups *and* likely to have affected the outcome? |  | NA / Y / PY / PN / N / NI |
|  | **If your aim for this study is to assess the effect of starting and adhering to intervention, answer questions 4.3 to 4.6** | |  |
|  | 4.3. Were important co-interventions balanced across intervention groups? | No. There are some disbalances across the intervention groups, specifically in macrolides, hydroxychloroquine. | Y / PY / PN / **N** / NI |
|  | 4.4. Was the intervention implemented successfully for most participants? | Yes. The intervention was implemented successfully for all participants. | **Y** / PY / PN / N / NI |
|  | 4.5. Did study participants adhere to the assigned intervention regimen? | Yes. Study participants adhered to the assigned intervention regimen. | **Y** / PY / PN / N / NI |
|  | 4.6. **If N/PN to 4.3, 4.4 or 4.5**: Was an appropriate analysis used to estimate the effect of starting and adhering to the intervention? | Yes. The authors used IPTW analysis to estimate the effect of starting and adhering to the intervention. | NA / **Y** / PY / PN / N / NI |
|  | **Risk of bias judgement** | **Moderate risk of bias:** ii) The important co-interventions were not balanced across the intervention groups. | Low / **Moderate** / Serious / Critical / NI |
|  | Optional: What is the predicted direction of bias due to deviations from the intended interventions? | Unpredictable | Favours experimental / Favours comparator / Towards null /Away from null / **Unpredictable** |

| **Bias due to missing data** | | | |
| --- | --- | --- | --- |
|  | 5.1 Were outcome data available for all, or nearly all, participants? | Yes. Outcome data was available for nearly all participants. On Figure 2, they show outcome data for 416 out of 420 patients that received the intervention vs 205 out of 210 of patients who did not receive the intervention. Missing data for a total of 9 patients. | **Y** / PY / PN / N / NI |
|  | 5.2 Were participants excluded due to missing data on intervention status? | No. Participants were not excluded due to missing data. | Y / PY / PN / **N** / NI |
|  | 5.3 Were participants excluded due to missing data on other variables needed for the analysis? | No. Participants were not excluded since there was no missing data on the other variables needed for the analysis. | Y / PY / PN / **N** / NI |
|  | 5.4 **If PN/N to 5.1, or Y/PY to 5.2 or 5.3**: Are the proportion of participants and reasons for missing data similar across interventions? |  | NA / Y / PY / PN / N / NI |
|  | 5.5 **If PN/N to 5.1, or Y/PY to 5.2 or 5.3**: Is there evidence that results were robust to the presence of missing data? |  | NA / Y / PY / PN / N / NI |
|  | **Risk of bias judgement** | **Low risk of bias:** i) Data were reasonably complete. | **Low** / Moderate / Serious / Critical / NI |
|  | Optional: What is the predicted direction of bias due to missing data? | Unpredictable | Favours experimental / Favours comparator / Towards null /Away from null / **Unpredictable** |

| **Bias in measurement of outcomes** | | | |
| --- | --- | --- | --- |
|  | 6.1 Could the outcome measure have been influenced by knowledge of the intervention received? | No. The primary outcome was hospital related, which is a hard outcome; so, no influence by knowledge of the intervention received could have been done. | Y / PY / PN / **N** / NI |
|  | 6.2 Were outcome assessors aware of the intervention received by study participants? | Yes. Since it is a retrospective cohort study, the outcome assessors were fully aware of the intervention received by study participants. | **Y** / PY / PN / N / NI |
|  | 6.3 Were the methods of outcome assessment comparable across intervention groups? | Yes. The primary outcome was hospital related mortality, which is a hard outcome; so, methods of outcome assessment were the same for both. | **Y** / PY / PN / N / NI |
|  | 6.4 Were any systematic errors in measurement of the outcome related to intervention received? | No. There were no systematic errors in measurement of the outcome, since it was a hard outcome. | Y / PY / PN / ***N*** / NI |
|  | **Risk of bias judgement** | **Low risk of bias:** i) The methods of outcome assessment were comparable across intervention groups AND ii) The outcome measure was unlikely to be influenced by knowledge of the intervention received by study participants AND iii) Any error in measuring the outcome is unrelated to intervention status. | **Low** / Moderate / Serious / Critical / NI |
|  | Optional: What is the predicted direction of bias due to measurement of outcomes? | Unpredictable | Favours experimental / Favours comparator / Towards null /Away from null / **Unpredictable** |

| **Bias in selection of the reported result** | | | |
| --- | --- | --- | --- |
|  | Is the reported effect estimate likely to be selected, on the basis of the results, from... |  |  |
|  | 7.1. ... multiple outcome *measurements* within the outcome domain? | No. The reported effect estimate was not likely to be selected from multiple outcome measurements. | Y / PY / PN / **N** / NI |
|  | 7.2 ... multiple *analyses* of the intervention-outcome relationship? | No. The reported effect estimate was not likely to be selected from multiple analyzed of the intervention-outcome relationship. Although, the authors did a Cox multivariable analysis and a propensity-score analysis; they presented all their complete analyses. | Y / PY / PN / **N** / NI |
|  | 7.3 ... different *subgroups*? | No. The reported effect estimate was not likely to be selected from different subgroups. | Y / PY / PN / **N** / NI |
|  | **Risk of bias judgement** | **Low risk of bias:** There is clear evidence that all reported results correspond to all intended outcome, analysis, and sub-cohorts. | **Low** / Moderate / Serious / Critical / NI |
|  | Optional: What is the predicted direction of bias due to selection of the reported result? | Unpredictable | Favours experimental / Favours comparator / Towards null /Away from null / **Unpredictable** |

| **Overall bias** | | | |
| --- | --- | --- | --- |
|  | **Risk of bias judgement** | **Serious risk of bias:** This study was judged to have serious risk of bias in one out of seven domains. | Low / Moderate / **Serious** / Critical / NI |
|  | Optional: What is the overall predicted direction of bias for this outcome? | Unpredictable | Favours experimental / Favours comparator / Towards null /Away from null / **Unpredictable** |


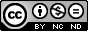


This work is licensed under a [Creative Commons Attribution-NonCommercial-NoDerivatives 4.0 International License](http://creativecommons.org/licenses/by-nc-nd/4.0/).

# ROBINS-I tool (Stage II): For each study

**Somers et al. Tocilizumab for treatment of MV patients with COVID-19. Clin Infec Diseases. 2020**

## Specify a target randomized trial specific to the study

| Design | Individually randomized / Cluster randomized / Matched (e.g. cross-over) |
| --- | --- |
| Participants | Adult Patients with severe SARS COV-2 pneumonia by RT PCR and required invasive mechanical ventilation. |
| Experimental intervention | Tocilizumab (1 dose 8mg/kg, only 2^nd^ dose – 4 patients. |
| Comparator | Standard of care |

## Is your aim for this study…?

| 🞩 | to assess the effect of *assignment to* intervention |
| --- | --- |
| □ | to assess the effect of *starting and adhering to* intervention |

## Specify the outcome

Specify which outcome is being assessed for risk of bias (typically from among those earmarked for the Summary of Findings table). Specify whether this is a proposed benefit or harm of intervention.

| Survival probability after intubation |
| --- |

## Specify the numerical result being assessed

In case of multiple alternative analyses being presented, specify the numeric result (e.g. RR = 1.52 (95% CI 0.83 to 2.77) and/or a reference (e.g. to a table, figure or paragraph) that uniquely defines the result being assessed.

| Model A: HR 0.54 (95%CI 0.29-1.0)  Model B IPTW with complete data: 0.55 (95%CI 0.33-0.90)  IPTW-MI adjusted HR 0.54 (95%CI 0.35-0.84) |
| --- |

## Preliminary consideration of confounders

Complete a row for each important confounding domain (i) listed in the review protocol; and (ii) relevant to the setting of this particular study, or which the study authors identified as potentially important.

#### “Important” confounding domains are those for which, in the context of this study, adjustment is expected to lead to a clinically important change in the estimated effect of the intervention. “Validity” refers to whether the confounding variable or variables fully measure the domain, while “reliability” refers to the precision of the measurement (more measurement error means less reliability).

| **(i) Confounding domains listed in the review protocol** | | | | |
| --- | --- | --- | --- | --- |
| Confounding domain | Measured variable(s) | Is there evidence that controlling for this variable was unnecessary?* | Is the confounding domain measured validly and reliably by this variable (or these variables)? | OPTIONAL: Is failure to adjust for this variable (alone) expected to favour the experimental intervention or the comparator? |
| There are no confounding domains described in the protocol. |  |  | Yes / No / No information | Favour experimental / Favour comparator / No information |

| **(ii) Additional confounding domains relevant to the setting of this particular study, or which the study authors identified as important** | | | | |
| --- | --- | --- | --- | --- |
| Confounding domain | Measured variable(s) | Is there evidence that controlling for this variable was unnecessary?* | Is the confounding domain measured validly and reliably by this variable (or these variables)? | OPTIONAL: Is failure to adjust for this variable (alone) expected to favour the experimental intervention or the comparator? |
| Demographics | Age, gender, race, BMI, obesity hypertension, diabetes, CV disease, pulmonary disease, smoking status | There is no evidence controlling was unnecessary | **Yes** / No / No information | Favour experimental / Favour comparator / **No information** |
| Baseline inflammatory markers | CRP, D-Dimer, LDH, ferritin | There is no evidence controlling was unnecessary | **Yes** / No / No information | Favour experimental / Favour comparator / **No information** |
| Baseline severity of disease | Timing of MV, Timing of TZM | There is no evidence controlling was unnecessary | **Yes** / No / No information | Favour experimental / Favour comparator / **No information** |

* In the context of a particular study, variables can be demonstrated not to be confounders and so not included in the analysis: (a) if they are not predictive of the outcome; (b) if they are not predictive of intervention; or (c) because adjustment makes no or minimal difference to the estimated effect of the primary parameter. Note that “no statistically significant association” is not the same as “not predictive”.

## Preliminary consideration of co-interventions

Complete a row for each important co-intervention (i) listed in the review protocol; and (ii) relevant to the setting of this particular study, or which the study authors identified as important.

#### “Important” co-interventions are those for which, in the context of this study, adjustment is expected to lead to a clinically important change in the estimated effect of the intervention.

| **(i) Co-interventions listed in the review protocol** | | |
| --- | --- | --- |
| Co-intervention | Is there evidence that controlling for this co-intervention was unnecessary (e.g. because it was not administered)? | Is presence of this co-intervention likely to favour outcomes in the experimental intervention or the comparator |
| There are no co-interventions described in the protocol. |  | Favour experimental / Favour comparator / No information |

| **(ii) Additional co-interventions relevant to the setting of this particular study, or which the study authors identified as important** | | |
| --- | --- | --- |
| Co-intervention | Is there evidence that controlling for this co-intervention was unnecessary (e.g. because it was not administered)? | Is presence of this co-intervention likely to favour outcomes in the experimental intervention or the comparator |
| Steroids | There is no evidence controlling for co-intervention was unnecessary. | Favour experimental / Favour comparator / **No information** |
| Hydroxychloroquine | There is no evidence controlling for co-intervention was unnecessary. | Favour experimental / Favour comparator / **No information** |
| Remdesivir | There is no evidence controlling for co-intervention was unnecessary. | Favour experimental / Favour comparator / **No information** |
| NSAIDS, ACE Inhibitors, Vasopressors, ECMO | There is no evidence controlling for co-intervention was unnecessary. | Favour experimental / Favour comparator / **No information** |

## Risk of bias assessment

Responses underlined in green are potential markers for low risk of bias, and responses in red are potential markers for a risk of bias. Where questions relate only to sign posts to other questions, no formatting is used.

|  | **Signalling questions** | **Description** | **Response options** |
| --- | --- | --- | --- |
| **Bias due to confounding** | | | |
|  | 1.1 Is there potential for confounding of the effect of intervention in this study?  **If N/PN to 1.1:** the study can be considered to be at low risk of bias due to confounding and no further signalling questions need be considered | Yes. There are potential confounders of the effect of intervention. | **Y** / PY / PN / N |
|  | **If Y/PY to 1.1**: determine whether there is a need to assess time-varying confounding: |  |  |
|  | 1.2. Was the analysis based on splitting participants’ follow up time according to intervention received?  **If N/PN**, answer questions relating to baseline confounding (1.4 to 1.6)  **If Y/PY**, go to question 1.3. | No. The intervention received did not change over time and post-baseline prognostic factors could not have affected the effect of intervention. | NA / Y / PY / PN / **N** / NI |
|  | 1.3. Were intervention discontinuations or switches likely to be related to factors that are prognostic for the outcome?  **If N/PN**, answer questions relating to baseline confounding (1.4 to 1.6)  **If Y/PY**, answer questions relating to both baseline and time-varying confounding (1.7 and 1.8) |  | NA / Y / PY / PN / N / NI |

|  | **Questions relating to baseline confounding only** | | |
| --- | --- | --- | --- |
|  | 1.4. Did the authors use an appropriate analysis method that controlled for all the important confounding domains? | Yes. The authors calculated propensity scores by multivariable logistic regression with potential confounders. They applied IPTW to create a pseudo study cohort. | NA / **Y** / PY / PN / N / NI |
|  | 1.5. **If Y/PY to 1.4**: Were confounding domains that were controlled for measured validly and reliably by the variables available in this study? | No. The authors measured demographic factors, clinical factors, laboratory tests, radiological tests, and medications validly and reliably to control confounding domains; but they did not measure clinical status at baseline to control for baseline severity of disease. | NA / Y / PY / PN / **N** / NI |
|  | 1.6. Did the authors control for any post-intervention variables that could have been affected by the intervention? | No. The authors did not control any post-baseline variables that could have affected the effect of intervention. | NA / Y / PY / PN / **N** / NI |
|  | **Questions relating to baseline and time-varying confounding** | |  |
|  | 1.7. Did the authors use an appropriate analysis method that controlled for all the important confounding domains and for time-varying confounding? | Yes. The authors used propensity score matching, adjusted Cox modelling to control the confounding domains and time-varying confounding. | NA / **Y** / PY / PN / N / NI |
|  | 1.8. **If Y/PY to 1.7**: Were confounding domains that were controlled for measured validly and reliably by the variables available in this study? | Yes. The authors measured demographic factors, clinical factors, laboratory tests and medications validly and reliably to control confounding domains; but they did not measure clinical status at baseline to control for baseline severity of disease. | NA / **Y** / PY / PN / N / NI |
|  | **Risk of bias judgement** | **Serious risk of bias:** i) At least one known important domain was not appropriately measured, or not controlled for. | Low / Moderate / **Serious** / Critical / NI |
|  | Optional: What is the predicted direction of bias due to confounding? | Unpredictable | Favours experimental / Favours comparator / **Unpredictable** |

| **Bias in selection of participants into the study** | | | |
| --- | --- | --- | --- |
|  | 2.1. Was selection of participants into the study (or into the analysis) based on participant characteristics observed after the start of intervention?  **If N/PN to 2.1:** go to 2.4 | No. The selection of participants into the study was not based on participants characteristics observed after the start of the intervention. | Y / PY / PN / **N** / NI |
|  | 2.2. **If Y/PY to 2.1**: Were the post-intervention variables that influenced selection likely to be associated with intervention?  2.3 **If Y/PY to 2.2**: Were the post-intervention variables that influenced selection likely to be influenced by the outcome or a cause of the outcome? |  | NA / Y / PY / PN / N / NI  NA / Y / PY / PN / N / NI |
|  | 2.4. Do start of follow-up and start of intervention coincide for most participants? | No. There are specific variables on Table 1 measuring the delay between the first symptoms and hospital admission, and study inclusion. | Y / PY / PN / **N** / NI |
|  | 2.5. **If Y/PY to 2.2 and 2.3, or N/PN to 2.4**: Were adjustment techniques used that are likely to correct for the presence of selection biases? | Yes. The authors used inverse probability weighting to correct for the presence of selection biases. | NA / **Y** / PY / PN / N / NI |
|  | **Risk of bias judgement** | **Moderate risk of bias:** ii) Start of the follow-up and start of the intervention do not coincide for all participants AND a) The authors used appropriate methods to adjust for the selection bias. | Low / **Moderate** / Serious / Critical / NI |
|  | Optional: What is the predicted direction of bias due to selection of participants into the study? | Unpredictable | Favours experimental / Favours comparator / Towards null /Away from null / **Unpredictable** |

| **Bias in classification of interventions** | | | |
| --- | --- | --- | --- |
|  | 3.1 Were intervention groups clearly defined? | Yes. The intervention and control groups were clearly defined. | **Y** / PY / PN / N / NI |
|  | 3.2 Was the information used to define intervention groups recorded at the start of the intervention? | Yes. The study was conducted in the US and they used Electronic Medical Records (EMR) and manual abstraction to define the start of the intervention. | **Y** / PY / PN / N / NI |
|  | 3.3 Could classification of intervention status have been affected by knowledge of the outcome or risk of the outcome? | Probably no. Classification of the intervention status was done retrospectively according to EMR, so misclassification was not likely. | Y / PY / **PN** / N / NI |
|  | **Risk of bias judgement** | **Moderate risk of bias:** i) Intervention status is well defined AND ii) Some aspects of the assignments of intervention status were done retrospectively. | Low / **Moderate** / Serious / Critical / NI |
|  | Optional: What is the predicted direction of bias due to classification of interventions? | Unpredictable | Favours experimental / Favours comparator / Towards null /Away from null / **Unpredictable** |

| **Bias due to deviations from intended interventions** | | | |
| --- | --- | --- | --- |
|  | **If your aim for this study is to assess the effect of assignment to intervention, answer questions 4.1 and 4.2** | |  |
|  | 4.1. Were there deviations from the intended intervention beyond what would be expected in usual practice? | No. There were no deviations from the intended intervention. | Y / PY / PN / **N** / NI |
|  | 4.2. **If Y/PY to 4.1**: Were these deviations from intended intervention unbalanced between groups *and* likely to have affected the outcome? |  | NA / Y / PY / PN / N / NI |
|  | **If your aim for this study is to assess the effect of starting and adhering to intervention, answer questions 4.3 to 4.6** | |  |
|  | 4.3. Were important co-interventions balanced across intervention groups? | No. There are some disbalances across the intervention groups, specifically in prone position, therapeutic anticoagulation. | Y / PY / PN / **N** / NI |
|  | 4.4. Was the intervention implemented successfully for most participants? | Yes. The intervention was implemented successfully for all participants. | **Y** / PY / PN / N / NI |
|  | 4.5. Did study participants adhere to the assigned intervention regimen? | Yes. Study participants adhered to the assigned intervention regimen. | **Y** / PY / PN / N / NI |
|  | 4.6. **If N/PN to 4.3, 4.4 or 4.5**: Was an appropriate analysis used to estimate the effect of starting and adhering to the intervention? | Yes. The authors used IPTW analysis to estimate the effect of starting and adhering to the intervention. | NA / **Y** / PY / PN / N / NI |
|  | **Risk of bias judgement** | **Moderate risk of bias:** ii) The important co-interventions were not balanced across the intervention groups. | Low / **Moderate** / Serious / Critical / NI |
|  | Optional: What is the predicted direction of bias due to deviations from the intended interventions? | Unpredictable | Favours experimental / Favours comparator / Towards null /Away from null / **Unpredictable** |

| **Bias due to missing data** | | | |
| --- | --- | --- | --- |
|  | 5.1 Were outcome data available for all, or nearly all, participants? | Yes. Outcome data was available for nearly all participants. IPTW subset with complete data 49 vs 67 and IPTW with imputed data for missing labs 78 vs 76. | **Y** / PY / PN / N / NI |
|  | 5.2 Were participants excluded due to missing data on intervention status? | No. Participants were not excluded due to missing data. Data was imputed. | Y / PY / PN / **N** / NI |
|  | 5.3 Were participants excluded due to missing data on other variables needed for the analysis? | No. Participants were not excluded since there was no missing data on the other variables needed for the analysis. | Y / PY / PN / **N** / NI |
|  | 5.4 **If PN/N to 5.1, or Y/PY to 5.2 or 5.3**: Are the proportion of participants and reasons for missing data similar across interventions? |  | NA / Y / PY / PN / N / NI |
|  | 5.5 **If PN/N to 5.1, or Y/PY to 5.2 or 5.3**: Is there evidence that results were robust to the presence of missing data? |  | NA / Y / PY / PN / N / NI |
|  | **Risk of bias judgement** | **Low risk of bias:** i) Data were reasonably complete. | **Low** / Moderate / Serious / Critical / NI |
|  | Optional: What is the predicted direction of bias due to missing data? | Unpredictable | Favours experimental / Favours comparator / Towards null /Away from null / **Unpredictable** |

| **Bias in measurement of outcomes** | | | |
| --- | --- | --- | --- |
|  | 6.1 Could the outcome measure have been influenced by knowledge of the intervention received? | No. The primary outcome was survival post intubation, which is a hard outcome; so, no influence by knowledge of the intervention received could have been done. | Y / PY / PN / **N** / NI |
|  | 6.2 Were outcome assessors aware of the intervention received by study participants? | Yes. Since it is a retrospective cohort study, the outcome assessors were fully aware of the intervention received by study participants. | **Y** / PY / PN / N / NI |
|  | 6.3 Were the methods of outcome assessment comparable across intervention groups? | Yes. The primary outcome was survival post intubation, which is a hard outcome; so, methods of outcome assessment were the same for both. | **Y** / PY / PN / N / NI |
|  | 6.4 Were any systematic errors in measurement of the outcome related to intervention received? | No. There were no systematic errors in measurement of the outcome, since it was a hard outcome. | Y / PY / PN / ***N*** / NI |
|  | **Risk of bias judgement** | **Low risk of bias:** i) The methods of outcome assessment were comparable across intervention groups AND ii) The outcome measure was unlikely to be influenced by knowledge of the intervention received by study participants AND iii) Any error in measuring the outcome is unrelated to intervention status. | **Low** / Moderate / Serious / Critical / NI |
|  | Optional: What is the predicted direction of bias due to measurement of outcomes? | Unpredictable | Favours experimental / Favours comparator / Towards null /Away from null / **Unpredictable** |

| **Bias in selection of the reported result** | | | |
| --- | --- | --- | --- |
|  | Is the reported effect estimate likely to be selected, on the basis of the results, from... |  |  |
|  | 7.1. ... multiple outcome *measurements* within the outcome domain? | No. The reported effect estimate was not likely to be selected from multiple outcome measurements. | Y / PY / PN / **N** / NI |
|  | 7.2 ... multiple *analyses* of the intervention-outcome relationship? | No. The reported effect estimate was not likely to be selected from multiple analyzed of the intervention-outcome relationship. Although, the authors did a Cox multivariable analysis and a propensity-score analysis; they presented all their complete analyses. | Y / PY / PN / **N** / NI |
|  | 7.3 ... different *subgroups*? | No. The reported effect estimate was not likely to be selected from different subgroups. | Y / PY / PN / **N** / NI |
|  | **Risk of bias judgement** | **Low risk of bias:** There is clear evidence that all reported results correspond to all intended outcome, analysis, and sub-cohorts. | **Low** / Moderate / Serious / Critical / NI |
|  | Optional: What is the predicted direction of bias due to selection of the reported result? | Unpredictable | Favours experimental / Favours comparator / Towards null /Away from null / **Unpredictable** |

| **Overall bias** | | | |
| --- | --- | --- | --- |
|  | **Risk of bias judgement** | **Serious risk of bias:** This study was judged to have serious risk of bias in one out of seven domains. | Low / Moderate / **Serious** / Critical / NI |
|  | Optional: What is the overall predicted direction of bias for this outcome? | Unpredictable | Favours experimental / Favours comparator / Towards null /Away from null / **Unpredictable** |


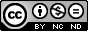


This work is licensed under a [Creative Commons Attribution-NonCommercial-NoDerivatives 4.0 International License](http://creativecommons.org/licenses/by-nc-nd/4.0/).

# ROBINS-I tool (Stage II): For each study

**Rodriguez-Baño et al. Treatment with Tocilizumab or corticosteroids for COVID-19 patients with hyperinflammatory state: a multicentre cohort study (SAM COVID-19). Clin Microb and Infec. 2020. 00:1-7**

## Specify a target randomized trial specific to the study

| Design | Individually randomized / Cluster randomized / **Matched (e.g. cross-over)** |
| --- | --- |
| Participants | Hospitalized, adult patients with PCR-confirmed SARS-CoV-2 infection and inflammatory response |
| Experimental intervention | Tocilizumab |
| Comparator | No treatment, Corticosteroids Intermediate-high dose, corticosteroids pulse dose, combination treatment. |

## Is your aim for this study…?

| **X** | **to assess the effect of *assignment to* intervention** |
| --- | --- |
| □ | to assess the effect of *starting and adhering to* intervention |

## Specify the outcome

Specify which outcome is being assessed for risk of bias (typically from among those earmarked for the Summary of Findings table). Specify whether this is a proposed benefit or harm of intervention.

| Intubation or Death, follow-up 21 days |
| --- |

## Specify the numerical result being assessed

In case of multiple alternative analyses being presented, specify the numeric result (e.g. RR = 1.52 (95% CI 0.83 to 2.77) and/or a reference (e.g. to a table, figure or paragraph) that uniquely defines the result being assessed.

| 10/88 vs 69/344  IPTW 🡪 HR: 0.32 (95%CI 0.22-0.47)  No IPTW 🡪 HR: 0.52 (95%CI 0.27-1.01) |
| --- |

## Preliminary consideration of confounders

Complete a row for each important confounding domain (i) listed in the review protocol; and (ii) relevant to the setting of this particular study, or which the study authors identified as potentially important.

#### “Important” confounding domains are those for which, in the context of this study, adjustment is expected to lead to a clinically important change in the estimated effect of the intervention. “Validity” refers to whether the confounding variable or variables fully measure the domain, while “reliability” refers to the precision of the measurement (more measurement error means less reliability).

| **(i) Confounding domains listed in the review protocol** | | | | |
| --- | --- | --- | --- | --- |
| Confounding domain | Measured variable(s) | Is there evidence that controlling for this variable was unnecessary?* | Is the confounding domain measured validly and reliably by this variable (or these variables)? | OPTIONAL: Is failure to adjust for this variable (alone) expected to favour the experimental intervention or the comparator? |
| There are no confounding domains described in the protocol. |  |  |  |  |

| **(ii) Additional confounding domains relevant to the setting of this particular study, or which the study authors identified as important** | | | | |
| --- | --- | --- | --- | --- |
| Confounding domain | Measured variable(s) | Is there evidence that controlling for this variable was unnecessary?* | Is the confounding domain measured validly and reliably by this variable (or these variables)? | OPTIONAL: Is failure to adjust for this variable (alone) expected to favour the experimental intervention or the comparator? |
| Demographics | Age, gender, hypertension, diabetes, obesity, CV disease, pulmonary disease, | There is no evidence controlling was unnecessary | **Yes** / No / No information | Favour experimental / Favour comparator / **No information** |
| Baseline inflammatory markers | CRP, D-Dimer, IL-6, ferritin, LDH | There is no evidence controlling was unnecessary | **Yes** / No / No information | Favour experimental / Favour comparator / **No information** |
| Baseline severity of disease | Oxygen support | There is no evidence controlling was unnecessary | Yes / **No** / No information | Favour experimental / Favour comparator / **No information** |

* In the context of a particular study, variables can be demonstrated not to be confounders and so not included in the analysis: (a) if they are not predictive of the outcome; (b) if they are not predictive of intervention; or (c) because adjustment makes no or minimal difference to the estimated effect of the primary parameter. Note that “no statistically significant association” is not the same as “not predictive”.

## Preliminary consideration of co-interventions

Complete a row for each important co-intervention (i) listed in the review protocol; and (ii) relevant to the setting of this particular study, or which the study authors identified as important.

#### “Important” co-interventions are those for which, in the context of this study, adjustment is expected to lead to a clinically important change in the estimated effect of the intervention.

| **(i) Co-interventions listed in the review protocol** | | |
| --- | --- | --- |
| Co-intervention | Is there evidence that controlling for this co-intervention was unnecessary (e.g. because it was not administered)? | Is presence of this co-intervention likely to favour outcomes in the experimental intervention or the comparator |
| There are no co-interventions described in the protocol. |  |  |

| **(ii) Additional co-interventions relevant to the setting of this particular study, or which the study authors identified as important** | | |
| --- | --- | --- |
| Co-intervention | Is there evidence that controlling for this co-intervention was unnecessary (e.g. because it was not administered)? | Is presence of this co-intervention likely to favour outcomes in the experimental intervention or the comparator |
| Antiretrovirals | There is no evidence controlling for co-intervention was unnecessary. | Favour experimental / Favour comparator / **No information** |
| Antimicrobials | There is no evidence controlling for co-intervention was unnecessary. | Favour experimental / Favour comparator / **No information** |
| Steroids | There is no evidence controlling for co-intervention was unnecessary. | Favour experimental / Favour comparator / **No information** |
| Anticoagulants | There is no evidence controlling for co-intervention was unnecessary. | Favour experimental / Favour comparator / **No information** |

## Risk of bias assessment

Responses underlined in green are potential markers for low risk of bias, and responses in red are potential markers for a risk of bias. Where questions relate only to sign posts to other questions, no formatting is used.

|  | **Signalling questions** | **Description** | **Response options** |
| --- | --- | --- | --- |
| **Bias due to confounding** | | | |
|  | 1.1 Is there potential for confounding of the effect of intervention in this study?  **If N/PN to 1.1:** the study can be considered to be at low risk of bias due to confounding and no further signalling questions need be considered | Yes. There are potential confounders of the effect of intervention. | **Y** / PY / PN / N |
|  | **If Y/PY to 1.1**: determine whether there is a need to assess time-varying confounding: |  |  |
|  | 1.2. Was the analysis based on splitting participants’ follow up time according to intervention received?  **If N/PN**, answer questions relating to baseline confounding (1.4 to 1.6)  **If Y/PY**, go to question 1.3. | No. The intervention received did not change over time and post-baseline prognostic factors could not have affected the effect of intervention. | NA / Y / PY / PN / **N** / NI |
|  | 1.3. Were intervention discontinuations or switches likely to be related to factors that are prognostic for the outcome?  **If N/PN**, answer questions relating to baseline confounding (1.4 to 1.6)  **If Y/PY**, answer questions relating to both baseline and time-varying confounding (1.7 and 1.8) |  | NA / Y / PY / PN / N / NI |

|  | **Questions relating to baseline confounding only** | | |
| --- | --- | --- | --- |
|  | 1.4. Did the authors use an appropriate analysis method that controlled for all the important confounding domains? | Yes. The authors used propensity scores by performing multivariate logistic regression. They calculated IPTW in Cox analysis to control the confounding domains. | NA / **Y** / PY / PN / N / NI |
|  | 1.5. **If Y/PY to 1.4**: Were confounding domains that were controlled for measured validly and reliably by the variables available in this study? | No. The authors measured demographic factors, clinical factors, laboratory tests and medications validly and reliably to control confounding domains; they measured clinical status at baseline to control for baseline severity of disease. They did not control for BMI or chest CT baseline parameters. | NA / Y / PY / PN / **N** / NI |
|  | 1.6. Did the authors control for any post-intervention variables that could have been affected by the intervention? | No. The authors did not control any post-baseline variables that could have affected the effect of intervention. | NA / Y / PY / PN / **N** / NI |
|  | **Questions relating to baseline and time-varying confounding** | |  |
|  | 1.7. Did the authors use an appropriate analysis method that controlled for all the important confounding domains and for time-varying confounding? | Yes. The authors used inverse probability weighting and Cox regression models to control the confounding domains and time-varying confounding. | NA / **Y** / PY / PN / N / NI |
|  | 1.8. **If Y/PY to 1.7**: Were confounding domains that were controlled for measured validly and reliably by the variables available in this study? | Yes. The authors measured demographic factors, clinical factors, laboratory tests, radiological tests, and medications validly and reliably to control confounding domains; but they did not measure BMI or radiographic signs of disease. | NA / **Y** / PY / PN / N / NI |
|  | **Risk of bias judgement** | **Serious risk of bias:** i) At least one known important domain was not appropriately measured, or not controlled for. | Low / Moderate / **Serious** / Critical / NI |
|  | Optional: What is the predicted direction of bias due to confounding? | Unpredictable | Favours experimental / Favours comparator / **Unpredictable** |

| **Bias in selection of participants into the study** | | | |
| --- | --- | --- | --- |
|  | 2.1. Was selection of participants into the study (or into the analysis) based on participant characteristics observed after the start of intervention?  **If N/PN to 2.1:** go to 2.4 | No. The participants selected had to be from before March 31 to assure 21 days of follow up. | Y / PY / PN / **N** / NI |
|  | 2.2. **If Y/PY to 2.1**: Were the post-intervention variables that influenced selection likely to be associated with intervention?  2.3 **If Y/PY to 2.2**: Were the post-intervention variables that influenced selection likely to be influenced by the outcome or a cause of the outcome? |  | NA / Y / PY / PN / N / NI  NA / Y / PY / PN / N / NI |
|  | 2.4. Do start of follow-up and start of intervention coincide for most participants? | No. The start of intervention was defined as administration of the intervention at <=2days from day 0. | Y / PY / PN / **N** / NI |
|  | 2.5. **If Y/PY to 2.2 and 2.3, or N/PN to 2.4**: Were adjustment techniques used that are likely to correct for the presence of selection biases? | Yes. The authors used inverse probability weighting to correct for the presence of selection biases. | NA / **Y** / PY / PN / N / NI |
|  | **Risk of bias judgement** | **Moderate risk of bias:** ii) Start of the follow-up and start of the intervention do not coincide for all participants AND a) The authors used appropriate methods to adjust for the selection bias. | Low / **Moderate** / Serious / Critical / NI |
|  | Optional: What is the predicted direction of bias due to selection of participants into the study? | Unpredictable | Favours experimental / Favours comparator / Towards null /Away from null / **Unpredictable** |

| **Bias in classification of interventions** | | | |
| --- | --- | --- | --- |
|  | 3.1 Were intervention groups clearly defined? | Yes. The intervention and control groups were clearly defined. | **Y** / PY / PN / N / NI |
|  | 3.2 Was the information used to define intervention groups recorded at the start of the intervention? | Yes. The study was conducted in Spain. They use Electronic Medical Records (EMR) to record the start of the intervention. | **Y** / PY / PN / N / NI |
|  | 3.3 Could classification of intervention status have been affected by knowledge of the outcome or risk of the outcome? | Probably no. Classification of the intervention status was done retrospectively according to EMR, so misclassification was not likely. | Y / PY / **PN** / N / NI |
|  | **Risk of bias judgement** | **Moderate risk of bias:** i) Intervention status is well defined AND ii) Some aspects of the assignments of intervention status were done retrospectively. | Low / **Moderate** / Serious / Critical / NI |
|  | Optional: What is the predicted direction of bias due to classification of interventions? | Unpredictable | Favours experimental / Favours comparator / Towards null /Away from null / **Unpredictable** |

| **Bias due to deviations from intended interventions** | | | |
| --- | --- | --- | --- |
|  | **If your aim for this study is to assess the effect of assignment to intervention, answer questions 4.1 and 4.2** | |  |
|  | 4.1. Were there deviations from the intended intervention beyond what would be expected in usual practice? | No. There were no deviations from the intended intervention. | Y / PY / PN / **N** / NI |
|  | 4.2. **If Y/PY to 4.1**: Were these deviations from intended intervention unbalanced between groups *and* likely to have affected the outcome? |  | NA / Y / PY / PN / N / NI |
|  | **If your aim for this study is to assess the effect of starting and adhering to intervention, answer questions 4.3 to 4.6** | |  |
|  | 4.3. Were important co-interventions balanced across intervention groups? | Yes. Important co-interventions were balanced across the intervention and control group. | **Y** / PY / PN / N / NI |
|  | 4.4. Was the intervention implemented successfully for most participants? | Yes. The intervention was implemented successfully for all participants. | **Y** / PY / PN / N / NI |
|  | 4.5. Did study participants adhere to the assigned intervention regimen? | Yes. Study participants adhered to the assigned intervention regimen. | **Y** / PY / PN / N / NI |
|  | 4.6. **If N/PN to 4.3, 4.4 or 4.5**: Was an appropriate analysis used to estimate the effect of starting and adhering to the intervention? |  | NA / Y / PY / PN / N / NI |
|  | **Risk of bias judgement** | **Low risk of bias:** i) Any deviation from the intended intervention reflected usual practice. | **Low** / Moderate / Serious / Critical / NI |
|  | Optional: What is the predicted direction of bias due to deviations from the intended interventions? | Unpredictable | Favours experimental / Favours comparator / Towards null /Away from null / **Unpredictable** |

| **Bias due to missing data** | | | |
| --- | --- | --- | --- |
|  | 5.1 Were outcome data available for all, or nearly all, participants? | Yes. Outcome data was available for all participants. | **Y** / PY / PN / N / NI |
|  | 5.2 Were participants excluded due to missing data on intervention status? | No. Participants were not excluded since there was no missing data on the intervention status. | Y / PY / PN / **N** / NI |
|  | 5.3 Were participants excluded due to missing data on other variables needed for the analysis? | No. Participants were not excluded since there was no missing data on the other variables needed for the analysis. | Y / PY / PN / **N** / NI |
|  | 5.4 **If PN/N to 5.1, or Y/PY to 5.2 or 5.3**: Are the proportion of participants and reasons for missing data similar across interventions? |  | NA / Y / PY / PN / N / NI |
|  | 5.5 **If PN/N to 5.1, or Y/PY to 5.2 or 5.3**: Is there evidence that results were robust to the presence of missing data? |  | NA / Y / PY / PN / N / NI |
|  | **Risk of bias judgement** | **Low risk of bias:** i) Data were reasonably complete. | **Low** / Moderate / Serious / Critical / NI |
|  | Optional: What is the predicted direction of bias due to missing data? | Unpredictable | Favours experimental / Favours comparator / Towards null /Away from null / **Unpredictable** |

| **Bias in measurement of outcomes** | | | |
| --- | --- | --- | --- |
|  | 6.1 Could the outcome measure have been influenced by knowledge of the intervention received? | No. The primary outcome was intubation or death, which is a hard outcome; so, no influence by knowledge of the intervention received could have been done. | Y / PY / PN / **N** / NI |
|  | 6.2 Were outcome assessors aware of the intervention received by study participants? | Yes. Since it is a retrospective cohort study, the outcome assessors were fully aware of the intervention received by study participants. | **Y** / PY / PN / N / NI |
|  | 6.3 Were the methods of outcome assessment comparable across intervention groups? | Yes. The primary outcome was intubation or death, which is a hard outcome; so, methods of outcome assessment were the same for both. | **Y** / PY / PN / N / NI |
|  | 6.4 Were any systematic errors in measurement of the outcome related to intervention received? | No. There were no systematic errors in measurement of the outcome, since it was a hard outcome. | Y / PY / PN / **N** / NI |
|  | **Risk of bias judgement** | **Low risk of bias:** i) The methods of outcome assessment were comparable across intervention groups AND ii) The outcome measure was unlikely to be influenced by knowledge of the intervention received by study participants AND iii) Any error in measuring the outcome is unrelated to intervention status. | **Low** / Moderate / Serious / Critical / NI |
|  | Optional: What is the predicted direction of bias due to measurement of outcomes? | Unpredictable | Favours experimental / Favours comparator / Towards null /Away from null / **Unpredictable** |

| **Bias in selection of the reported result** | | | |
| --- | --- | --- | --- |
|  | Is the reported effect estimate likely to be selected, on the basis of the results, from... |  |  |
|  | 7.1. ... multiple outcome *measurements* within the outcome domain? | No. The reported effect estimate was not likely to be selected from multiple outcome measurements. | Y / PY / PN / **N** / NI |
|  | 7.2 ... multiple *analyses* of the intervention-outcome relationship? | No. The reported effect estimate was not likely to be selected from multiple analysed of the intervention-outcome relationship. Although, the authors did an unadjusted multivariate analysis and propensity-score analysis (one including inverse probability weighting); they presented all their complete analyses. | Y / PY / PN / **N** / NI |
|  | 7.3 ... different *subgroups*? | No. The reported effect estimate was not likely to be selected from different subgroups. | Y / PY / PN / **N** / NI |
|  | **Risk of bias judgement** | **Low risk of bias:** There is clear evidence that all reported results correspond to all intended outcome, analysis, and sub-cohorts. | **Low** / Moderate / Serious / Critical / NI |
|  | Optional: What is the predicted direction of bias due to selection of the reported result? | Unpredictable | Favours experimental / Favours comparator / Towards null /Away from null / **Unpredictable** |

| **Overall bias** | | | |
| --- | --- | --- | --- |
|  | **Risk of bias judgement** | **Serious risk of bias:** This study was judged to have serious risk of bias in one out of seven domains. | Low / Moderate / **Serious** / Critical / NI |
|  | Optional: What is the overall predicted direction of bias for this outcome? | Unpredictable | Favours experimental / Favours comparator / Towards null /Away from null / **Unpredictable** |


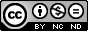


This work is licensed under a [Creative Commons Attribution-NonCommercial-NoDerivatives 4.0 International License](http://creativecommons.org/licenses/by-nc-nd/4.0/).
